# Universal and Interpretable Descriptor to Design Dual‐atom Catalysts for Multi‐Type C–C Coupling with Ultrahigh C_2+_ Yield

**DOI:** 10.1002/advs.202512614

**Published:** 2025-09-16

**Authors:** Yuming Gao, Chenyi Guo, Juncheng Hong, Haoyu Yan, Jinbiao Zhang, Zhe Wang, Dawei Tang, Bo Jiang

**Affiliations:** ^1^ School of Energy and Power Engineering Key Laboratory of Ocean Energy Utilization and Energy Conservation of Ministry of Education Dalian University of Technology Dalian 116024 China

**Keywords:** C─C coupling, Descriptor, Dual‐atom catalysts (DACs), High‐throughput screening, Machine learning (ML)

## Abstract

Dual‐atom catalysts (DACs) have demonstrated superior performance in the C─C coupling processes to generate multi‐carbon (C_2+_) products due to the multiple metal sites of DACs. However, the rational design of DACs that exhibit high activity and specific selectivity for the C─C coupling process in the CO_2_ reaction remains a significant challenge due to the intricate inter‐metal interaction. Herein, a universal and interpretable descriptor is proposed for the emerging metal‐nitrogen‐carbon (M_1_–M_2_–N_4_–C) dual‐atom catalysts in the CH^*^─CH^*^ coupling process, effectively evaluating the activity and selectivity for various C─C coupling (i.e., CH^*^─CH^*^, CO^*^─CO^*^, CHO^*^─CO^*^, CH^*^─CH_2_^*^). This defined descriptor, which consists of inherent atomic properties (electronegativity, d electron number), proves to facilitate high‐throughput screening of more than 400 graphene‐based DACs. Through this screening process, potential catalysts such as FeZr@NC are predicted to have superior activity and selectivity for C─C coupling with a C_2+_ yield of 22.6%, outperforming the state‐of‐the‐art catalysts by a factor of 3 to 30. Importantly, this descriptor can also be extended to evaluate the activity and selectivity of DACs for other C─C coupling processes (i.e., CO^*^─CO^*^, CHO^*^─CO^*^, and CH^*^─CH_2_^*^), with very small discrepancies between the prediction and computational results. By systematically integrating machine learning (ML) with physical insights, this work offers practical guidelines for the construction of universal descriptors for DACs based on inherent atomic properties and the logical design of advanced catalysts in C─C coupling.

## Graphical abstract

CO_2_ capture and conversion, along with green hydrogen, offer considerable potential for achieving carbon neutrality and yielding high‐value multi‐carbon (C_2+_) products. Nevertheless, CO_2_‐to‐C_2+_ conversion is challenging, primarily due to the extreme inertness of CO_2_, the high C─C coupling barrier, and many competing reactions yielding C_1_ products. Dual‐atom catalysts (DACs) exhibit outstanding catalytic performance with flexible active sites, shedding light on the exploration of CO_2_‐to‐C_2+_ conversion. A universal and interpretable descriptor is proposed to directly predict C_2+_ yield, a unique capability that has not yet been achieved by other descriptors. This descriptor, which consists of inherent atomic properties such as electronegativity and d electron number, enables inexpensive, rapid, and high‐throughput catalyst screening.

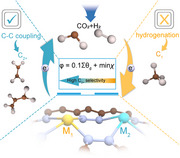

## 1 Introduction

The utilization of CO_2_ resources into energy‐dense fuels and high‐value‐added chemicals, which is a crucial strategy in achieving the pivotal goal of peaking CO_2_ emissions and attaining carbon neutrality, holds the potential to mitigate CO_2_ emissions and enable large‐scale renewable energy storage.^[^ ^1^ ^]^ Extensive scientific research has focused on the process of CO_2_ hydrogenation, a reaction that predominantly generates C_1_ compounds such as CO and CH_4_, achieving notable progress in this realm.^[^ ^2^, ^3^ ^]^ Furthermore, in comparison to C_1_ products, the multi‐carbon (C_2+_) hydrocarbons not only possess high energy density but also provide the benefits of easy transportation and storage, rendering them significant in CO_2_ reduction.^[^ ^4^ ^]^ Nonetheless, the activity and selectivity of CO_2_ hydrogenation catalysts employed for C_2+_ products reported are far from satisfactory because of the greater reaction energy barrier for the C─C coupling than those of C─H or C─O bond formation.^[^ ^5^ ^]^ Consequently, the key to the efficient generation of C_2+_ products lies in the rational construction of active sites that are able to strikingly mitigate the energy barrier of the C─C coupling process.^[^ ^6^ ^]^ Recently, dual‐atom catalysts (DACs) have emerged as a paradigm in the field on account of their superior properties, such as extensive specific surface area, commendable thermal stability, and elevated atomic utilization.^[^ ^7^, ^8^ ^]^ More crucially, the presence of dual sites in DACs facilitates the concurrent absorption of multiple C_1_ intermediates, thereby offering substantial convenience for C─C coupling. Furthermore, the asymmetric charge distribution within the C_1_ intermediates significantly attenuates their electrostatic repulsion, thus enabling the C─C coupling reactions to proceed with enhanced efficiency.^[^ ^9^, ^10^ ^]^ Nonetheless, the complexity of the interactions between metals and supports presents a formidable challenge in the rational design and development of superior DACs for C─C coupling that exhibit both high activity and selectivity.

Fortunately, the advent of artificial intelligence has ushered in a transformative era, significantly expediting the DACs discovery process through enhanced algorithms and advancements in data science.^[^ ^11^ ^]^ Machine learning (ML), an efficient and pragmatic artificial intelligence framework based on computer and statistical science, is used to develop algorithms to learn from density functional theory (DFT) computational data and data from existing databases to predict specific results without explicit programming, mitigating computational expenses and accelerating high‐throughput screening.^[^ ^12^ ^]^ Despite much progress, the ML algorithm or the process of producing an output is entirely opaque.^[^ ^13^ ^]^ Therefore, it is almost impossible to reasonably explain the relationship between input features and results from a mechanistic point of view, posing a significant challenge for revealing the structure‐performance relationship.^[^ ^14^ ^]^ In stark contrast, descriptors can effectively address this issue by associating simple catalyst parameters with complex catalytic performance (e.g., limiting potential, adsorption energy, activation barriers, or turnover frequency) from the in‐depth explanation of catalyst characteristics and reaction mechanism.^[^ ^15^, ^16^ ^]^ Nonetheless, when it comes to descriptors, the extensive array of features that govern catalytic performance presents a significant challenge in devising a rational structure purely based on theoretical insights and experiential knowledge. A diverse range of ML analytical methodologies has proven their efficacy in reducing the dimensionality of features and selecting pertinent components of descriptors.^[^ ^17^, ^18^ ^]^ Previous studies have established several activity descriptors for several reactions, including the orbital filling of transition metals, the distance between the active metals, the density of active metal centers for transition‐metal oxides, and the distance between the d/p band centers.^[^ ^19^, ^20^, ^21^, ^22^ ^]^ For DACs, a descriptor has been developed based on DFT and topologically information‐based ML to accelerate the rational design of DACs for oxygen evolution (OER) and oxygen reduction (ORR) reactions, thereby revealing 511 and 855 possible candidates for OER and ORR.^[^ ^23^ ^]^ Nevertheless, these properties cannot be directly obtained from databases, rendering it impractical to optimize catalytic performance or design promising catalysts based solely on these descriptors.^[^ ^24^ ^]^ Meanwhile, several descriptors are not interpretable; accordingly, it is possibly unable to comprehensively explain the structure‐performance relationship from a theoretical point of view.^[^ ^25^ ^]^ For CO_2_ hydrogenation reactions, a multitude of activity descriptors have been logically devised and demonstrated to be efficacious for the C_1_ products.^[^ ^26^, ^27^ ^]^ For example, an inherent descriptor has been formulated to characterize the activity of CO_2_ reduction to CH_4_ for single‐atom catalysts.^[^ ^28^ ^]^ Nevertheless, given the prevalence of numerous side reactions in C─C coupling, the descriptor necessitates the consideration of both selectivity and activity. So, an ideal low‐cost descriptor, composed of intrinsic atomic properties, that is capable of concurrently predicting activity and selectivity, *i.e*., directly predicting product yield, is of paramount importance and a remarkable challenge, especially when considering its practical utility, reliability, and universal applicability.^[^ ^25^, ^29^, ^30^, ^31^, ^32^, ^33^, ^34^ ^]^

**Figure 1:**
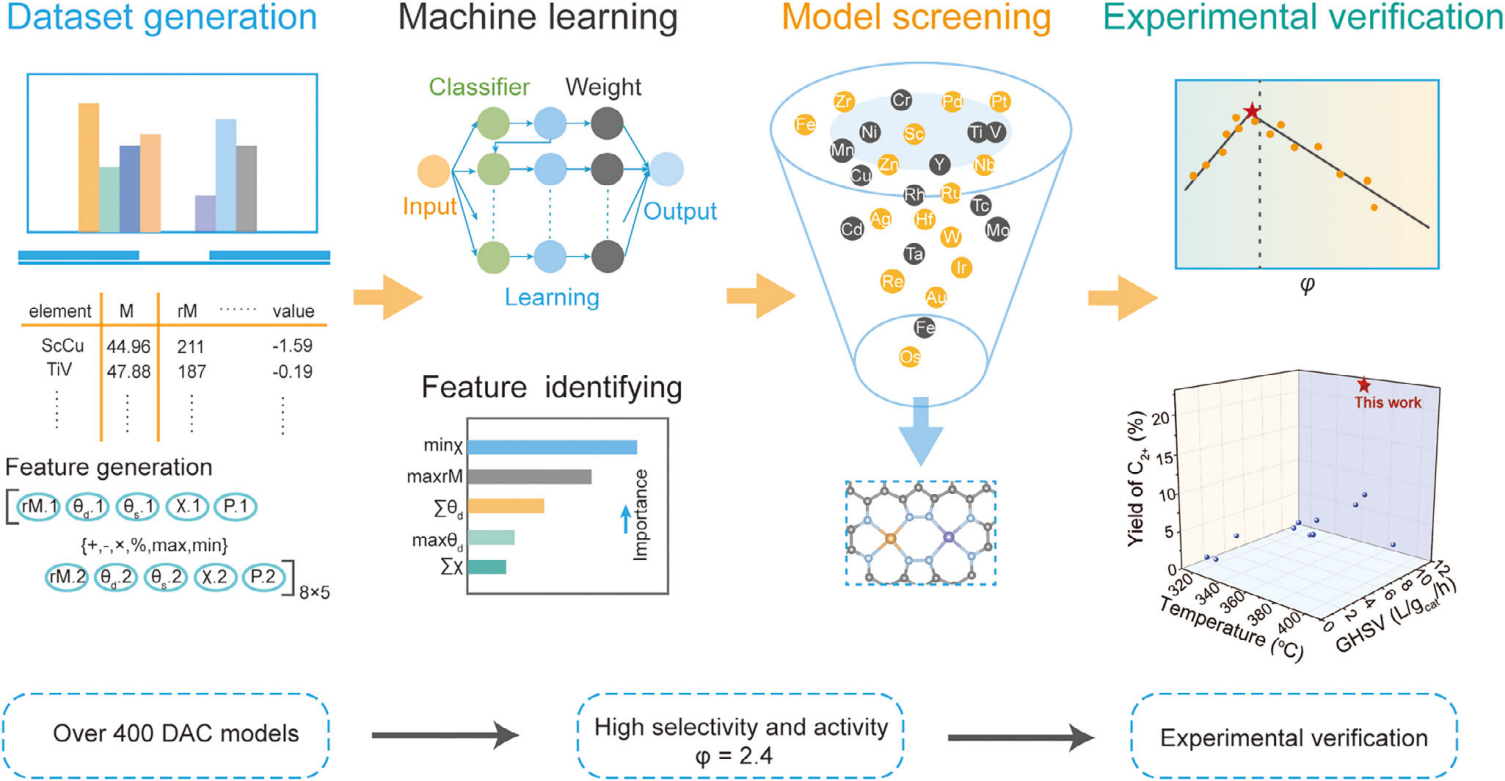
Workflow diagram from ML model construction, high‐throughput screening, descriptor construction, and experimental validation.

Herein, by integrating DFT calculations, feature refining through the ML method, and in‐depth elucidation of catalytic mechanisms, we develop a universal and interpretable descriptor (*φ* = 0.1Σ*_d_* + min*χ*) to assess the catalytic performance of M_1_–M_2_–N_4_–C DACs for several different C─C coupling processes. This intrinsic descriptor is composed solely of inherent atomic properties (d electron number (*θ_d_*) and electronegativity (*χ*)) that can be directly obtained from the database. Concurrently, this interpretable descriptor provides a comprehensive elucidation of the structure‐performance relationship at the molecular orbital level. Building on this foundation, by reasonably measuring the complex inter‐metal interaction, this descriptor can effectively expedite high‐throughput screening and identify the activity and selectivity trends for the CH^*^─CH^*^ coupling process over a variety of DACs and predict catalysts with excellent activity and selectivity. For instance, FeZr@NC achieved remarkable performance in C─C coupling, with a C_2+_ yield of 22.6%, outperforming the state‐of‐the‐art catalysts by a factor ranging from 3 to 30. The workflow diagram of this ML‐assisted tactic is shown in **Figure** **1**, which consists of four parts: pre‐generation of data and models, ML, descriptor construction with interpretation, and experimental validation. In addition, this descriptor can be used to further measure the activity and selectivity of other C─C coupling processes, i.e., CO^*^─CO^*^, CHO^*^─CO^*^, and CH^*^─CH_2_^*^, where the error between the prediction and computational results is considerably small. Our design principles for developing descriptors may provide a lot of inspiration for the high‐throughput screening of advanced catalysts and be extendable to other reactions and other materials.

## 2 Results and Discussion

### 2.1 Model Construction and Calculations

In the contemporary scientific landscape, a plethora of DACs with diverse substrates, such as Nitrogen‐doped Carbon (NC), MXenes, and Metal‐Organic Frameworks (MOFs), have been identified.^[^ ^35^, ^36^, ^37^ ^]^ Among these, the nascent M_1_–M_2_–N_4_–C DACs have garnered significant attention due to their extensive applications in various photocatalytic and electrocatalytic processes.^[^ ^38^, ^39^, ^40^ ^]^ The metal constituents of these catalysts are predominantly transition metals, whose incomplete d orbitals facilitate the possibility of electron exchange. The varying number of d electrons can effectively modulate the adsorption energy of intermediates, thereby providing rational control over the progression of the reaction. Ultimately, this regulation enables a wide array of chemical reactions to occur. To design and screen efficient catalysts for C─C coupling reactions, we have initially constructed models of M_1_–M_2_–N_4_–C DACs composed of 3d, 4d, and 5d transition metals (excluding Hg), and the structural representation of these models is depicted in Figure S1 (Supporting Information). For C─C coupling reactions, there are various possible pathways, with the primary C─C coupling processes being CH^*^─CH^*^, CO^*^─CO^*^, CHO^*^─CO^*^, and CH^*^─CH_2_^*^ (Figure S2, Supporting Information). We selected the CH^*^─CH^*^ coupling as a representative case for subsequent discussion. The performance evaluation of the DACs was conducted from the perspectives of activity and selectivity to maximize both simultaneously. The Gibbs free energy (Δ*G*) of the CH^*^─CH^*^ coupling step was used as the criterion for assessing the reaction activity (Δ*G*_activity_ = – Δ*G*_coupling_), given that the C─C coupling step is the rate‐determining step of the coupling reaction,^[^ ^41^ ^]^ and there is a robust linear correlation between the C─C coupling activation barrier (kinetic barriers) and the corresponding thermodynamic energy (Δ*G*).^[^ ^42^ ^]^ For selectivity assessment, the difference in Δ*G* between the CH^*^─CH^*^ coupling step and the hydrogenation step was used as the criterion for evaluating the reaction selectivity (ΔΔ*G*_selectivity_ = Δ*G*_coupling_ – Δ*G*_hydrogenation_). The different intermediate adsorption models are provided in Figures S3 and S4 (Supporting Information), establishing the foundational models for subsequent analysis. Subsequently, we developed a strategy combining ML and descriptors to efficiently design and screen DACs for C─C coupling reactions with high activity and selectivity.

**Figure 2:**
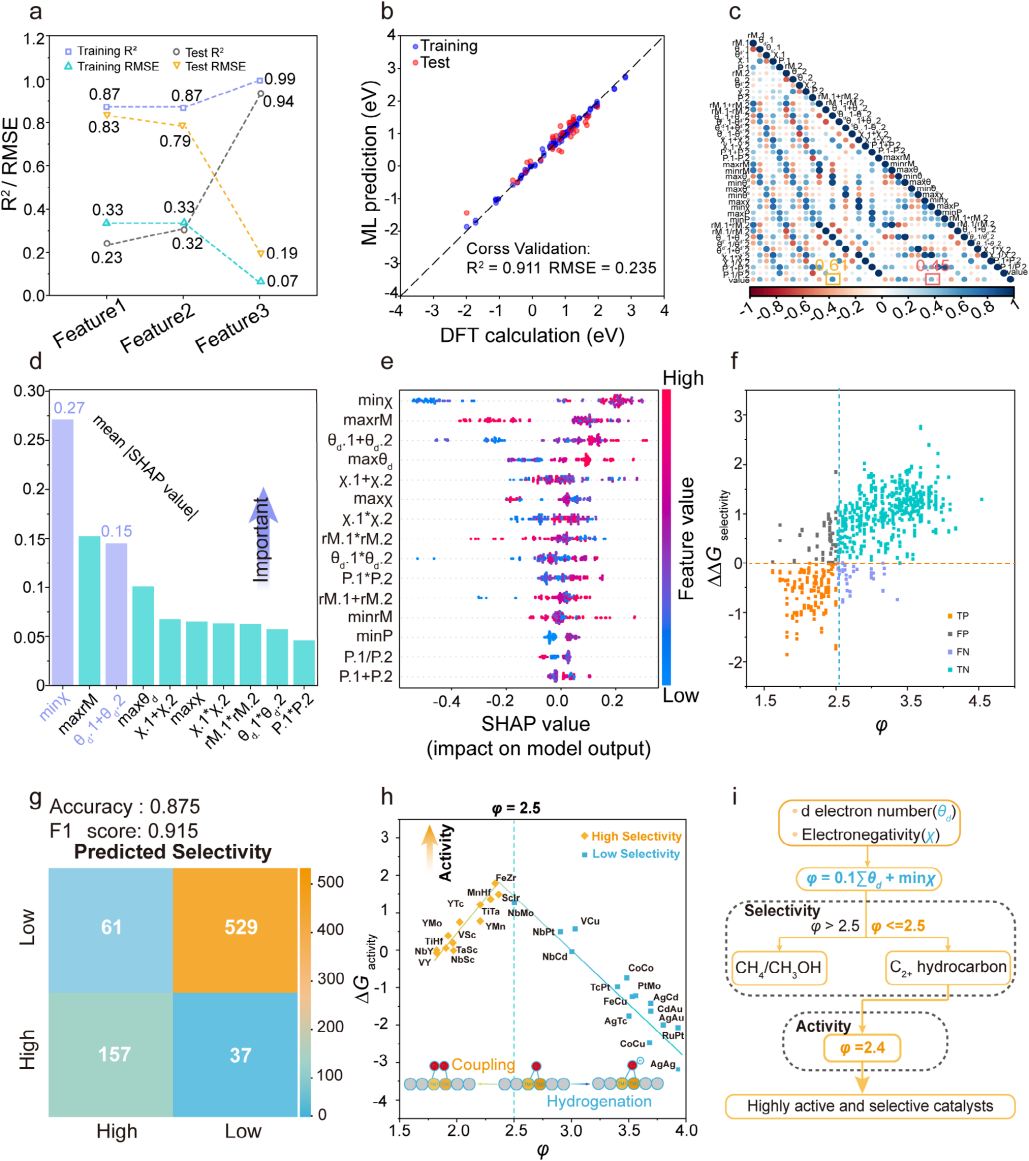
a) Input feature screening and expansion process based on different input feature sets. b) Comparison of DFT computed ΔΔ*G*_selectivity_ values with those predicted by the GBR model. c) The heatmap of the Pearson correlation coefficient matrix among the selected features in the input feature sets, Feature 3, and the output value (ΔΔ*G*_selectivity_). The subscript 1 represents the M_1_ atom of DACs, while those with subscript 2 represent the M_2_ atom of DACs. d) The significance of the top ten features, as determined by SHAP analysis, is highlighted, while features of lesser importance are omitted from consideration. e) SHAP beeswarm plot for ΔΔ*G*_selectivity_ prediction. (f) ΔΔ*G*_selectivity_ predicted by ML toward descriptor *φ*. g) Confusion matrix for actual selectivity and predicted selectivity by the descriptor *φ*. h) Volcano plot for activity versus *φ* of different DACs. i) Design or the screening procedure for the high activity of DACs with high selectivity for C─C, guided by the proposed descriptor *φ*.

In the realm of ML model construction, identifying suitable and comprehensible input features is an essential step. Consequently, the extraction of rational input features from an extensive array of available material features, which are pertinent to catalytic selectivity and activity, is a cardinal step in this investigation. Following the input Feature screening principles (Supplementary Note 1), we have selected 12 inherent features of two transition metals (M_1_, M_2_) that can be directly obtained from the database (*M*, *rM*, *θ_d_*, *θ_s_*, *N_e_*, *N_atom_*, *χ*, *EA*, *I*, *N_men_*, *N_group_*, *P*), detailed information listed in Table S1 (Supporting Information), as the initial input feature set (Feature 1). The quality of ML models depends critically on the input features, necessitating careful screening and expansion of input features. Initially, to enhance training efficiency and reduce prediction error, we employ the Pearson correlation coefficient to assess the correlation between different input features (Figure S5, Supporting Information). Features with a correlation coefficient greater than 0.75 are considered highly correlated, implying that one of them can, in principle, replace others to reduce the feature set without losing key information. As illustrated in Figure S5a (Supporting Information), *M* and *P*, *N_atom_*, *θ_d_*, and *I*, *N_men_*, *χ*, and *EA* exhibit a high correlation. Consequently, we have appropriately screened the input features, eliminating those with excessively high correlation, and the remaining features constitute our input feature set, Feature 2. Afterward, considering the intricate interaction between the two metal atoms of DACs, we incorporate the mathematical relationship (+, ‐, ×, ÷, max, min) that corresponds to its input features in Feature 2, creating an additional dimension in our input feature set, which we designate as Feature 3. (Except for *θ_s_*.1/*θ_s_*.2, since the number of s electrons in transition metals may be zero). To validate the effectiveness of the above feature selection and expansion, we establish an ML model based on the GBR algorithm, obtaining the R^2^ and RMSE of the training set and test set of various input feature sets, as illustrated in **Figure** **2**a. Among them, the input feature set Feature 3 showed the highest R^2^ (0.995 and 0.911) and the lowest RMSE (0.065 and 0.235) for the training set and test set, respectively, proving the rationality and effectiveness of the above process with improved accuracy and reduced errors. Ultimately, Feature 3 was identified as the input feature for the subsequent ML process. The screening flowchart is outlined in Figure S6, and Tables S2 and S3 (Supporting Information) list the specific feature values and the features associated with these input feature sets, respectively.

### 2.2 Machine Learning Performing

Upon completing the input feature selection, we randomly selected 68 samples from more than 400 candidadtes (Since both M_1_ and M_2_ were selected from the 28 transition metals, excluding Hg, given that the two active sites of DACs, M_1_–M_2_–N_4_–C and M_2_–M_1_–N_4_–C, are equivalent, all 406 permutations of M_1_ and M_2_ combinations structures were generated) using our random generator for DFT calculation to evaluate their ΔΔ*G*_selectivity_, and the specific values of which are listed in Table S4 (Supporting Information). The selected input feature set (Feature 3) from the above process and ΔΔ*G*_selectivity_, which served as the prediction target, were used as the original input data. Ten different ML algorithms, including Decision Tree Regression (DTR), Extra Tree Regression (ETR), Gradient Boost Regression (GBR), K‐Neighbor Regression (KNR), Kernel Ridge Regression (KRR), Logistic Regression (LR), Random Forest (RF), eXtreme Gradient Boosting (XGBR), Generalized Boosted Regression Models (GBM), and Support Vector Regression (SVR), were implemented to train the ML models. We utilize the coefficient of determination (R^2^) alongside root mean squared error (RMSE) as metrics for the training and evaluation, where a high R^2^ and a low RMSE indicate an effective model. Ten‐fold cross‐validation was used to test the model on the pre‐split validation set to ensure that no severe overfitting occurs. The GBR model exhibited exceptional performance, with an RMSE of only 0.065 eV in the training set and an R^2^ score of 0.991 (Figure S7 and Note S2, Supporting Information), and the performance of the GBR model across the entire dataset, including both training and validation sets, is clearly illustrated in Figures 2b and Figure S8 (Supporting Information). Hence, we used the GBR model to predict the performance of DACs that have not undergone calculations. Given that the two active sites of DACs, M_1_–M_2_–N_4_–C and M_2_–M_1_–N_4_–C, are equivalent, the symmetrical nature of the heatmap along the *y* = *x* diagonal provided insight into the precision of the model's predictions (Figure S10, Supporting Information). Even though the comprehensive strategy of integrating DFT and ML has accurately estimated the selectivity ΔΔ*G*_selectivity_ and reduced the computational cost, it is still challenging to provide a deep explanation for the origin of performance from a theoretical perspective and clarify the specific relationship between inherent features and selectivity. Here, by combining ML analytical methods and physical insights, we proposed a simple and interpretable descriptor *φ*, which rationally and clearly explains the structure‐performance relationship between inherent features and selectivity from the perspective of molecular orbitals, to achieve a deep explanation for the structural origin of selectivity. To comprehensively clarify the complex relationship between input features and output values in the GBR model, we used SHapley Additive exPlanations (SHAP) to clarify the impact of these features on the model.^[^ ^43^ ^]^ As shown in Figure 2d, the minimum electronegativity (min*χ*) of M_1_ and M_2_, the maximum radius (max*rM*) of M_1_ and M_2_, and the sum of the d electron numbers of M_1_ and M_2_ (*θ_d_*.1+*θ_d_*.2) play a crucial role in determining the output value. The influence of different features on the output values, including their specific magnitude and direction, is depicted in Figure 2e, which evaluates the SHAP values for the 15 input features employed. According to Figure 2e and Supplementary Note 2, Σ*θ_d_* and min*χ* were considered as potential foundational elements of the descriptor, offering crucial insights for future descriptor development. Analogously, the Pearson correlation analysis sheds light on the development of descriptors, as depicted in Figure 2c, where Σ*θ_d_* and min*χ* demonstrate strong correlations with the output values of 0.61 and 0.45, respectively, proving that they are indelibly tied to the output value. Moreover, the feature importance assessment from the GBR model exhibited a similar trend, highlighting Σ*θ_d_* and min*χ* as the two most essential features. Hence, we decided to select Σ*θ_d_* and min*χ* as potential components of the descriptor.

### 2.3 Descriptor Construction

To develop an effective descriptor for predicting selectivity, we introduced adjustable weight parameters, a and b, reflecting the relative importance of the key attributes, expressed as *φ_i_* = a × Σ*θ_d_* + b × min*χ*. Afterward, we optimized the values of a and b to maximize the accuracy of the descriptor, involving systematically testing a range of varying weight combinations during our initial evaluation, and concurrently, we treated the ΔΔ*G*_selectivity_ suggested by the GBR algorithm as the true measurement. As depicted in Figure 2f and Figure S12 (Supporting Information), these descriptors exhibit a weak linear relationship with the true value, and the same *φ* is associated with a considerable disparity in ΔΔ*G*_selectivity_ values, prompting us to use a suboptimal evaluation method to gauge the accuracy of the descriptor. Accordingly, we introduced the concept of the confusion matrix in the ML classification algorithm. The confusion matrix is a specific table layout used to visualize the performance of supervised learning algorithms, especially classification algorithms.^[^ ^44^ ^]^ Commonly, the evaluation of classification performance via the confusion matrix relies on accuracy or the F1 score. In cases of sample imbalance, the F1 score tends to provide an accurate reflection of the model's effectiveness compared with accuracy. Hence, the objective function was designed to maximize the F1 score, which in turn enhanced the accuracy of the descriptor *φ*. The associations between these descriptors, characterized by differing weights and selectivity, are depicted in Figures 2f and Figure S12 (Supporting Information). These figures were all composed of four parts, each of which was associated with one of the four cells in each confusion matrix (Figures 2g; Figure S13, Supporting Information), and a high ratio of true positive (TP) and true negative (TN) samples within the overall dataset enhanced the reliability of the model, reflecting the definition of accuracy. By utilizing a simple Python program, we were able to find the specific values for the relative weights a and b. Ultimately, we constructed a simple descriptor denoted as *φ* = 0.1Σ*θ_d_* + min*χ*. As depicted in Figure 2g, this descriptor, with its unique weight arrangement, records an accuracy of 0.875 and an F1 score of 0.915, both of which exceed the performance of the other descriptors that utilize varying weights, emphasizing its high accuracy and providing strong evidence for its selectivity effectiveness. Σ*θ_d_* represents the theoretical d electron number of the transition metal, and min*χ* represents the impact of charge rearrangement due to electronegativity differences. The combination of these two elements influences the total count of d electrons, quantified through our proposed local electron aggregation intensity (LEAI) descriptor. When the value of *φ* was less than or equal to 2.5, the selectivity of the catalyst exhibited high performance. Conversely, when *φ* exceeded 2.5, the catalytic selectivity was expected to deteriorate (Figure 2h).

To further screen out suitable catalysts that exhibit both excellent selectivity and superior activity, we continued to analyze and present the effectiveness of the descriptor in terms of activity. We assessed the Δ*G*_activity_ of 15 DACs that represent both the highest and lowest selectivity to gain a deep understanding of how DACs behave at various selectivity levels, as detailed in Table S5 (Supporting Information). Fortunately, we found that the descriptor *φ* displayed a strong volcano‐shape relationship with activity (Figure 2h), and its accuracy in fitting was significantly superior to the descriptors utilizing other weight numbers (Figure S14, Supporting Information). Moreover, through the application of a linear fit for the volcano plot, we discovered that the peak occurred at *φ* = 2.4, which was employed for subsequent evaluations of DACs. Furthermore, to verify the above statement, we calculated the C─C coupling kinetic barriers (ΔE_a_), utilizing CI‐NEB methods, by randomly selecting nine samples in a *φ* range from 1.5 to 4.0 (Figure S15, Supporting Information). We found that the kinetic barriers of these DACs, indeed, align with the thermodynamic energetics in this work, showcasing a volcano‐shaped activity plot (Figure S16, Supporting Information). In summary, both previous research and our calculations suggest that it is reasonable to predict activity using Δ*G*. Utilizing the strategy outlined above, we obtained DACs with high activity and selectivity in C─C coupling processes (Figure 2i), such as FeZr@NC.

### 2.4 Mechanism Explanation and Universality of the Descriptor

**Figure 3:**
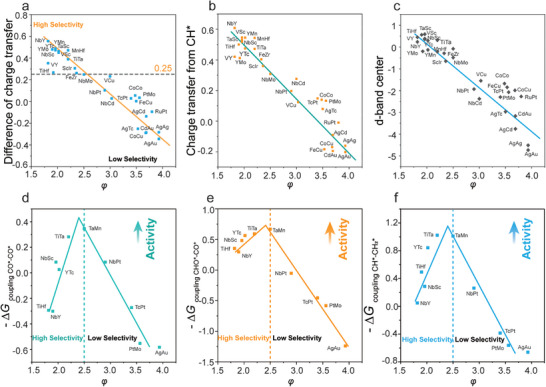
a) Difference of charge transfer and *φ*. b) charge transfer from CH^*^ and *φ*. c) Calculated d‐band center and *φ*. relationship between the C─C coupling activity for d) – Δ*G*_coupling CO*─CO*_ for CO^*^─CO^*^ coupling, e) – Δ*G*_coupling CHO*─CO*_ for CHO^*^─CO^*^ coupling, f) – Δ*G*_coupling CH*─CH2*_ for CH^*^–CH_2_^*^ coupling for different DACs and the descriptor *φ*. The dotted line is the dividing line between high and low selectivity.

We proposed the descriptor *φ* = 0.1Σ*θ_d_* + min*χ*, where Σ*θ_d_* represents the theoretical number of d electrons in transition metals, and min*χ* represents the effect of electron rearrangement due to electronegativity differences between metals and nitrogen/carbon supports. The combination of these two items affects the degree of actual electron aggregation at the active sites of the catalyst, which is quantified through our proposed local electron aggregation intensity (LEAI) descriptor. Then, we elaborated the physical‐chemical significance of our descriptor in view of selectivity and activity. As for selectivity, Chen *et al.* ^[^ ^45^ ^]^ demonstrated that an electron‐deficient state at the active site favors C─C coupling instead of hydrogenation, consistent with our Bader analysis (Figure S17 and Table S7, Supporting Information). Small descriptor values correspond to a diminished LEAI, i.e., the active site in an electron‐deficient state, making it prone to C─C coupling. Conversely, when descriptor values are large, the active site is in an electron‐rich state and is prone to hydrogenation (Figures S17 and S18, Supporting Information). Specifically, when the LEAI (*φ*) of the active site is low, the attraction of electrons is strong, making it easier to obtain electrons from CH^*^ and therefore facilitating the C─C coupling process. **Figure** **3**a quantifies the difference in electron transfer during the C─C coupling and hydrogenation processes. For activity, as the *φ* value (LEAI) increased, the d band center of the active metal sites tended to shift toward more negative values (Figure 3c; Table S8, Supporting Information), thereby weakening the adsorption of reaction intermediates such as CH^*^. Accordingly, the adsorption and desorption strengths of intermediates can be determined by the LEAI of the active sites. Moreover, based on the Sabatier principle, the excessive adsorption strength of intermediate CH^*^ results in the reaction persisting in its intermediate state, hindering the desorption of C_2+_ products and leading to catalyst poisoning by intermediates; conversely, if the adsorption is too weak, intermediates will quickly release, thereby inhibiting C─C coupling. Thus, we established a volcano‐shaped relationship between *φ* and activity. In addition, the relationship between charge transfer from CH^*^ and activity also exhibited a volcano‐shaped relationship (Figure S19, Supporting Information). FeZr@NC catalysts with moderate CH^*^ binding energies and low reaction energies of C─C coupling were predicted to be the most efficient candidates to produce the C_2+_ products when compared with other DACs.

As the descriptor *φ* was associated with the essential properties of DACs, it is reasonable to believe that *φ* is capable of not only screening out effective catalysts for CH^*^─CH^*^ but also possesses a high degree of universality for other C─C coupling reactions, such as CO^*^─CO^*^, CHO^*^─CO^*^, and CH^*^–CH_2_^*^. Consequently, to assess its universality regarding different C─C coupling reactions, additional C─C coupling processes were also evaluated for consideration. As illustrated in Figure 3d–f, the relationship between activity and *φ* shows a consistent volcano‐shaped relationship, regardless of whether it involves CO^*^─CO^*^, CHO^*^─CO^*^, or CH^*^─CH_2_^*^. Similarly, a *φ* value that was less than or equal to 2.5 was linked to superior selectivity (Table S9, Supporting Information). After linear coupling of the discrete points, it was observed that, regardless of the differences in C─C coupling methods, the vertices of their volcano plots consistently align at ≈*φ* = 2.5, confirming the universal applicability of the perfect catalyst acquired through the above screening strategy for various C─C coupling processes. As for the descriptor we proposed that can predict the activity of different coupling reactions, we make a further justification. In different coupling reactions, the key step is the coupling between various carbon‐containing intermediates, which is influenced by the adsorption strength of the intermediates. Moreover, Nørskov and co‐workers found that the adsorption energies of CH*_x_* (*x* = 1, 2, 3) and CHO, as well as CO adsorption energies, exhibit strong linear scaling relations (LSRs) over various transition‐metal surfaces.^[^ ^46^ ^]^ These results indicate that if a DAC exhibits strong adsorption for CH*_x_* (*x* = 1, 2, 3), it also exhibits strong adsorption for CO and CHO, and vice versa. Consequently, this descriptor has excellent predictive ability for CH─CH coupling reactions and also provides good predictions for the reactivity of other coupling reactions involving various carbon‐containing intermediates, such as CO^*^─CO^*^, CHO^*^─CO^*^, and CH^*^─CH_2_^*^.

In addition, despite the focus being on DACs@NC for assessing C─C coupling activity and selectivity, the methodology is adapted for discovering descriptors applicable to different kinds of supported catalysts. The impact of substrates outside of graphene on the microenvironment was not considered in this research. It is crucial to recognize that, despite the ability of the LEAI descriptor to reveal structure‐activity relationships in diverse C─C coupling reactions, future high‐throughput screening of DACs on alternative substrates should involve further refinement of this descriptor, tailored to the specific traits of each substrate, to improve predictive accuracy. In brief, our research results affirm that combining ML and mechanism explanation enables the proposed universally interpretable descriptor to clearly and comprehensively clarify the structure‐performance relationship that is often absent in high‐throughput screening for C─C coupling reactions and provide reasonable predictions about catalysts with superior performance.

### 2.5 Experimental verification

**Figure 4:**
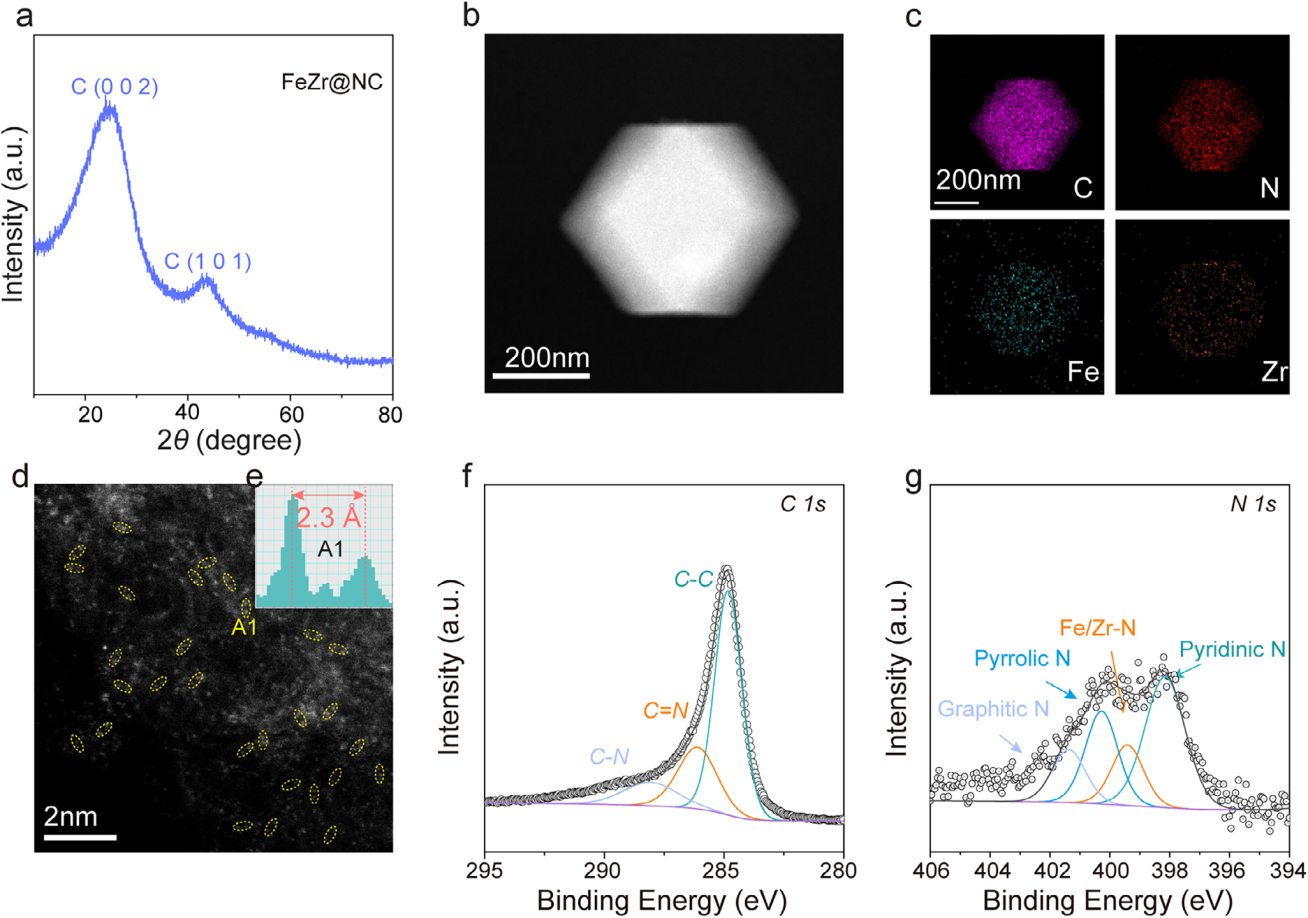
a) XRD patterns of FeZr@NC. b,c) TEM images and EDS mapping of FeZr@NC. d,e) AC‐HAADF‐STEM images of FeZr@NC and the intensity distributions corresponding to areas A1. f) C 1s and g) N 1s XPS spectra of FeZr@NC.

**Figure 5:**
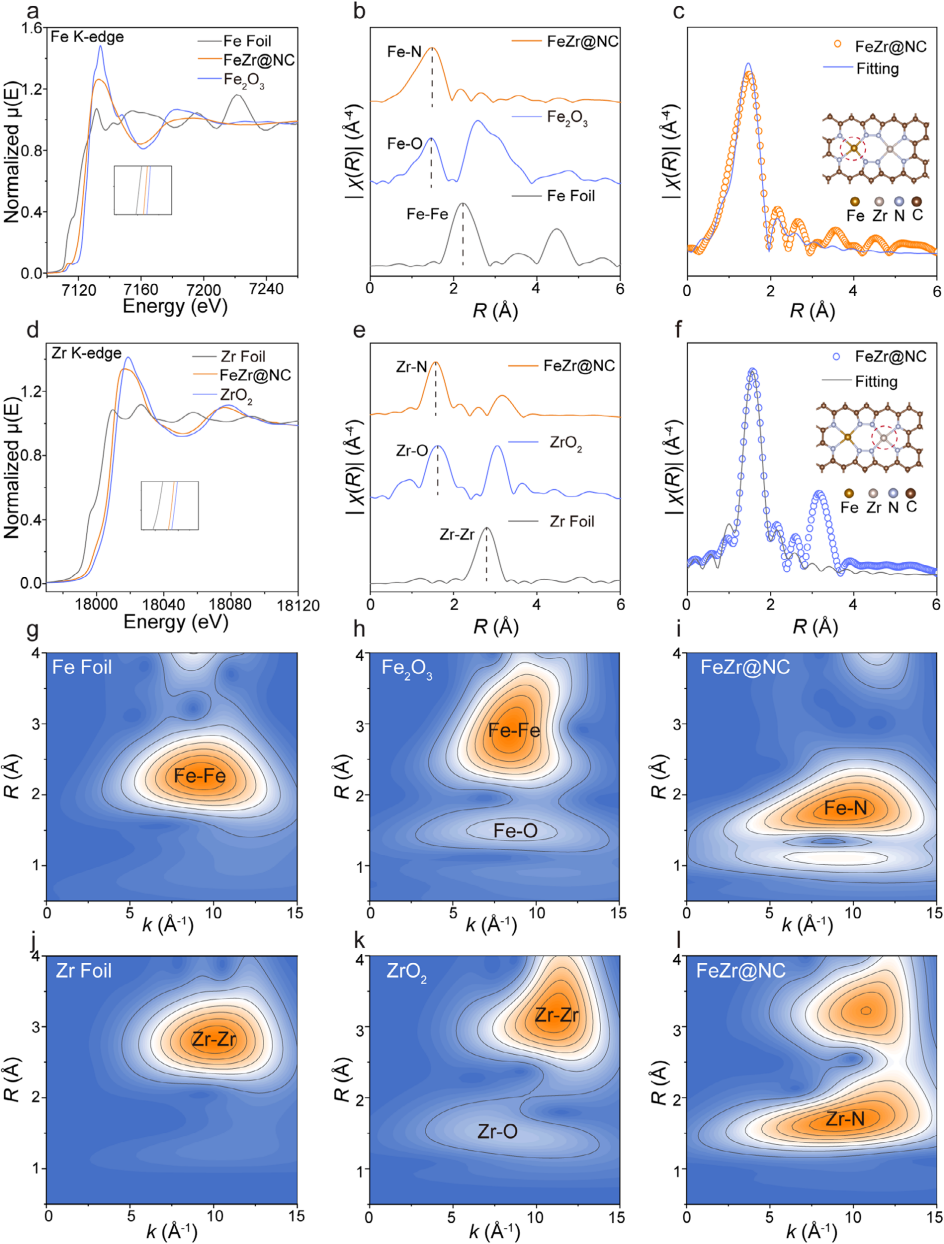
a) Fe K‐edge XANES spectra of FeZr@NC and reference samples. b) FT‐EXAFS spectra of FeZr@NC, Fe_2_O_3_, and Fe foil. c) The corresponding FT‐EXAFS fitting curves of FeZr@NC. d) Zr K‐edge XANES spectra of FeZr@NC and reference samples. e) FT‐EXAFS spectra of FeZr@NC, ZrO_2_, and Zr foil. f) The corresponding FT‐EXAFS fitting curves of FeZr@NC. WT‐EXAFS spectra of Fe in g) Fe foil, h) Fe_2_O_3_, and i) FeZr@NC. WT‐EXAFS spectra of Zr in j) Zr foil, k) ZrO_2_, and l) FeZr@NC.

To confirm the accuracy of the screening results, based on the predicted volcano plot (Figure 2h), we randomly synthesized a series of DACs with different descriptor values using the impregnation method for C─C coupling experiments. FeZr@NC was projected to possess superior activity and selectivity according to the descriptor *φ*. The X‐ray diffraction (XRD) pattern of FeZr@NC showed solely two peaks related to graphitic carbon peaks at roughly 25° and 44° (**Figure** **4**a), and there were no detectable peaks for metal particles. According to the elemental mappings from energy‐dispersive X‐ray spectroscopy (EDS), Fe and Zr were consistently distributed on the carbon matrix (Figure 4b,c). Through the use of spherical aberration‐corrected high‐angle annular dark‐field STEM (HAADF‐STEM), Fe─Zr diatomic pairs are directly visualized as bright spots in Figure 4d, with a preference for being located at the edges of nanopores. The intensity graphs depicted in Figure 4e (region A1) reveal an interatomic distance of ≈2.3 Å between Fe and Zr. Significantly, the two neighboring atoms in the intensity profiles exhibited different contrasts, confirming that the atomic pair consists of two different elements. The inductively coupled plasma optical emission spectroscopy (ICP‐OES) analysis confirmed that the Fe and Zr loadings were 0.59 wt% and 1.32 wt%, respectively, indicating that the molar ratio of Fe to Zr is ≈1:1. Utilizing X‐ray photoelectron spectroscopy (XPS), the elemental characteristics and chemical states of the surface material are assessed, as shown in Figures 4f,g and Figure S21 (Supporting Information). In the high‐resolution C 1s XPS spectra of FeZr@NC, the presence of C─C (284.8 eV), C═N (286.1 eV), and C─N (288.0 eV) peaks indicates that metal atoms do not chemically bond with carbon atoms (Figure 4f; Table S12, Supporting Information). Moreover, the XPS analysis of the FeZr@NC samples revealed a peak corresponding to Fe/Zr─N at a binding energy of 399.4 eV in the N 1s spectrum (Figure 4g), confirming that metal atoms are successfully loaded on the carbon matrix. The oxidation states of Zr and Fe were identified through the analysis of the Zr 3d and Fe 2p XPS spectra (Figure S21, Supporting Information). To shed light on the electronic structure and local coordination environment of the FeZr@NC catalyst, further analyses were conducted using X‐ray absorption near‐edge structure (XANES) and extended X‐ray absorption fine structure (EXAFS) spectroscopy. According to the Fe K‐edge XANES spectra, the absorption edge for FeZr@NC is found to be between the Fe foil and Fe_2_O_3_, as illustrated in **Figure** **5**a. In the case of the Zr K‐edge XANES, the absorption edge was located between the Zr foil and ZrO_2_ (Figure 5d). These XANES spectra demonstrate that both Fe and Zr exist in an oxidized state, aligning with the XPS result. Figure 5b,e illustrates that the Fourier‐transformed (FT) *k* ^3^‐weighted EXAFS spectra of FeZr@NC samples exhibit notable peaks ≈1.47 and 1.57 Å, corresponding to the first shell coordination of metal‐N. The coordination framework of the metal centers was analyzed using least‐squares fitting on the EXAFS data for Fe and Zr. As illustrated in Figure 5c, the Fe K‐edge EXAFS spectrum aligns well with the Fe─N and Fe─Zr scattering paths, exhibiting coordination numbers of 4.5 and 1.0 (Table S13, Supporting Information). Simultaneously, the *R*‐space curve of Zr was also fitted with the Zr─N and Zr─Fe paths, showing the coordination numbers of 4.2 and 1.2 (Figure 5f; Table S14, Supporting Information), respectively, as represented in the theoretical model (inset, Figure 5f). Moreover, to rule out other possible coordination configurations at the Fe and Zr sites within the FeZr@NC catalysts, we intended to fit the EXAFS curves with various scattering models (M_2_N_6_ or M_2_N_4_). The Δ*E_o_* and *R*‐factor of Models 2, 3, and 4 considerably exceeded the selection criteria (Tables S13 and Figure S14, Supporting Information),^[^ ^47^, ^48^ ^]^ thereby strongly indicating that the sample does not possess the M_2_N_6_ or M_2_N_4_ structure. To further explore the coordination environments at the atomic scale, Wavelet‐transform (WT) analysis was also carried out. As represented in Figure 5i,l, the maximum intensity values for Fe and Zr in FeZr@NC (9.55 Å^−1^ and 9.45 Å^−1^ in *k*‐space) differ from those of the Fe foil (9.25 Å^−1^) and Zr foil (10.05 Å^−1^), effectively confirming that there is no metal clustering and affirming the formation of heteronuclear Fe─Zr diatomic pairs, in agreement with the aforementioned outcomes of HAADF‐STEM and XRD. These thorough analyses validate that stabilized FeZr dual‐site structures are effectively embedded in N‐doped carbon frameworks.^[^ ^49^ ^]^

**Figure 6:**
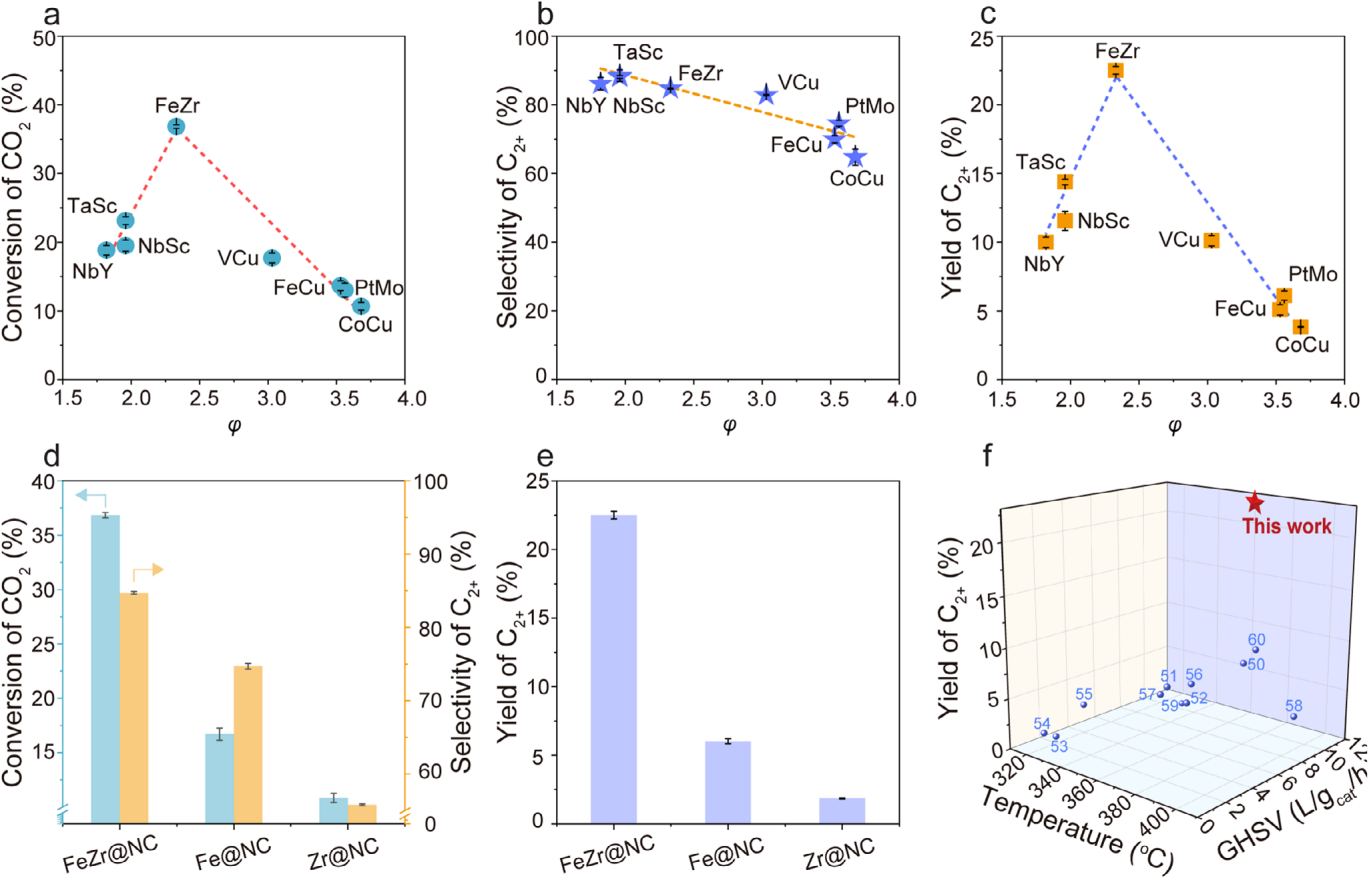
a) The experimental C─C coupling activity as a volcano‐like function of *φ*. b) The experimental C─C selectivity as a linear relationship of *φ*. c) The experiment for the yield of C_2+_ as a volcano‐like function of *φ*. d) CO_2_ conversion and C_2+_ selectivity of FeZr@NC, Fe@NC, and Zr@NC. e) The yield of C_2+_ of FeZr@NC, Fe@NC, and Zr@NC. f) Comparison of the yield of C_2+_ hydrocarbons between FeZr@NC and other typical catalysts used in CO_2_ into C_2+_.^[^ ^50^, ^51^, ^52^, ^53^, ^54^, ^55^, ^56^, ^57^, ^58^, ^59^, ^60^ ^]^ 50: Ref,^[^ ^50^ ^]^ 51: Ref,^[^ ^51^ ^]^ 52: Ref,^[^ ^52^ ^]^ 53: Ref,^[^ ^53^ ^]^ 54: Ref,^[^ ^54^ ^]^ 55: Ref,^[^ ^55^ ^]^ 56: Ref,^[^ ^56^ ^]^ 57: Ref,^[^ ^57^ ^]^ 58: Ref,^[^ ^58^ ^]^ 59: Ref,^[^ ^59^ ^]^ 60: ref. [60] Error bars correspond to the standard deviation of three independent measurements.

The C─C coupling performance of the designed DACs was evaluated, and all prepared DACs showed activity for C─C coupling, yet they exhibited noteworthy distinctions in the conversion of reactants and the distributions of products. The experimental activity of CO_2_ conversion over these catalysts, indeed, aligned with the theoretical predictions, showcasing a volcano‐shaped isolation‐activity plot with FeZr@NC recognized as the optimal catalyst (**Figure** **6**a), and the selectivity for C_2+_ observed in these catalysts corresponded well with the theoretical expectation, showing a linear relationship (Figure 6b) that highlights the reliability of activity predictions. Further analyses of the products confirmed the outstanding performance of the FeZr@NC catalyst, exhibiting a low C_1_ yield. The yield of C_2+_ was elevated, suggesting that the catalyst is promising for the generation of highly value‐added C_2+_ products (Figure 6c). Significantly, this finding validated theoretical expectations that the FeZr@NC catalyst showed exceptional catalytic performance in C─C coupling, even exceeding that of DACs with precious metals. As shown in Figure 6d,e, FeZr@NC achieves a higher CO_2_ conversion, superior selectivity for C_2+_, and C_2+_ yield than Fe@NC, and it is considerably greater than Zr@NC at 360 °C. This enhanced performance by FeZr@NC with asymmetric dual sites induces localized charge density redistribution, which results in the creation of appropriate local electron aggregation intensity, stabilizing C_1_ intermediates and reducing the C─C coupling barrier, ultimately achieving unprecedented C_2+_ yield. Our best‐performing catalyst, FeZr@NC, displayed superior C_2+_ yield up to 22.6% with a CO_2_ conversion of 36.8%, and the C_2+_ yield was 3–30 times higher than previously reported state‐of‐the‐art catalysts under similar reaction conditions (Figure 6f; Table S15, Supporting Information).^[^ ^50^, ^51^, ^52^, ^53^, ^54^, ^55^, ^56^, ^57^, ^58^, ^59^, ^60^ ^]^ In addition to the high activity toward CO_2_ conversion and high selectivity to C_2+_, FeZr@NC also exhibited a good catalytic stability, as evidenced by consistent conversion, selectivity, and C_2+_ yield over a 30 h stability test (Figure S24, Supporting Information). The stability was further confirmed by the XRD and XPS analyses of the spent FeZr@NC catalyst (Figures S25 and S26, Supporting Information). In addition, there was no obvious aggregation of the used catalyst, as demonstrated by TEM images and EDS mapping of the spent FeZr@NC (Figure S27, Supporting Information). It is concluded that the ML‐based large data set is practically advantageous for catalyst exploration, emphasizing the reliability of the predicted activities.

## 3 Conclusion

In conclusion, we devised a universally interpretable descriptor by amalgamating ML analytical techniques and profound elucidation of the catalytic mechanism. Significantly, this descriptor, composed exclusively of inherent features, is capable of accurately and concurrently predicting DACs with superior selectivity and activity for C─C coupling, thus eliminating the need for further computational analysis to screen the optimal catalyst with ultra‐high C_2+_ yields. Moreover, it has the ability to elucidate the basis of the catalytic action and selectivity of DACs in C─C coupling reactions by engaging in a comprehensive exploration of charge transfer during the reaction process. Based on the descriptor, we predicted DACs for C─C coupling reactions that exhibit both excellent activity and selectivity, such as FeZr@NC, which showed exceptional catalytic performance in C─C coupling with a C_2+_ yield of 22.6%, outperforming the state‐of‐the‐art catalysts by a factor of 3 to 30 times. Notably, the proposed descriptor has the potential to act as a universal descriptor for assessing selectivity and activity in multi‐type C─C coupling reactions, such as CH^*^─CH^*^, CO^*^─CO^*^, CHO^*^─CO^*^, and CH^*^–CH_2_^*^. The apexes of the catalytic activity volcano plots for these diverse C─C coupling reactions are located at nearly identical positions (*φ* = 2.4), implying that the predicted catalysts possess commendable performance for various C─C coupling reactions.

## Conflict of Interest

The authors declare no conflict of interest

## Supporting information



Supporting Information

## Data Availability

The data that support the findings of this study are available from the corresponding author upon reasonable request.

## References

[1] J. Y. Y. Loh , N. P. Kherani , G. A. Ozin , Nat. Sustain. 2021, 4, 466.

[2] Z. K. Xin , M. Y. Huang , Y. Wang , et al., Angew Chem Int Ed Engl 2022, 61, 202207222.

[3] Q. Liu , H. Cheng , T. Chen , T. W. B. Lo , Z. Xiang , F. Wang , Energy Environ. Sci. 2022, 15, 225.

[4] D. Gao , R. M. Arán‐Ais , H. S. Jeon , B. R Cuenya , Nat. Catal. 2019, 2, 198.

[5] J. Albero , Y. Peng , H. García , ACS Catal. 2020, 10, 5734.

[6] J. Zhu , W. Shao , X. Li , X. Jiao , J. Zhu , Y. Sun , Yi Xie , J. Am. Chem. Soc. 2021, 143, 18233. 34677975 10.1021/jacs.1c08033

[7] X. Hai , Y. Zheng , Qi Yu , Na Guo , S. Xi , X. Zhao , S. Mitchell , X. Luo , V. Tulus , Mu Wang , X. Sheng , L. Ren , X. Long , J. Li , P. He , H. Lin , Y. Cui , X. Peng , J. Shi , J. Wu , C. Zhang , R. Zou , G. Guillén‐Gosálbez , J. Pérez‐Ramírez , M. J. Koh , Ye Zhu , J. Li , J. Lu , Nature 2023, 622, 754. 37730999 10.1038/s41586-023-06529-z

[8] L. Wang , H. Wang , J. Lu , Chem Catalysis 2023, 3, 100492.

[9] G. Wang , Z. Chen , T. Wang , D. Wang , J. Mao , Angew. Chem., Int. Ed. 2022, 61, 202210789. 10.1002/anie.202210789 35969480

[10] J. M. Wang , Q. Y. Zhu , J. H. Lee , et al., Nat. Commun. 2023, 14, 3808. 37369676 10.1038/s41467-023-39580-5 PMC10300110

[11] M. Sun , H. H. Wong , T. Wu , A. W. Dougherty , B. Huang , Adv. Energy Mater. 2021, 11, 2101404.

[12] M. Zhong , K. Tran , Y. Min , C. Wang , Z. Wang , C. T. Dinh , P. De Luna , Z. Yu , A. S. Rasouli , P. Brodersen , S. Sun , O. Voznyy , C. S. Tan , M. Askerka , F. Che , M. Liu , A. Seifitokaldani , Y. Pang , S. C. Lo , A. Ip , Z. Ulissi , E. H. Sargent , Nature 2020, 581, 178. 32405017 10.1038/s41586-020-2242-8

[13] A. J. London , Hastings Cent Rep 2019, 49, 15. 10.1002/hast.973 30790315

[14] C. W. Schmidt , Environ. Health Perspect. 2020, 128, 22001. 32101024 10.1289/EHP5878 PMC7064317

[15] L. Wang , C. Zhu , M. Xu , C. Zhao , J. Gu , L. Cao , X. Zhang , Z. Sun , S. Wei , Wu Zhou , W. X. Li , J. Lu , J. Am. Chem. Soc. 2021, 143, 18854. 34730347 10.1021/jacs.1c09498

[16] X. Liu , Y. Jiao , Y. Zheng , M. Jaroniec , S. Z. Qiao , J. Am. Chem. Soc. 2019, 141, 9664. 31145607 10.1021/jacs.9b03811

[17] M. Zafari , D. Kumar , M. Umer , K. S. Kim , J. Mater. Chem. A 2020, 8, 5209.

[18] M. Zafari , A. S. Nissimagoudar , M. Umer , G. Lee , K. S. Kim , J. Mater. Chem. A 2021, 9, 9203.

[19] H. B. Tao , L. Fang , J. Chen , H. B. Yang , J. Gao , J. Miao , S. Chen , B. Liu , J. Am. Chem. Soc. 2016, 138, 9978. 27441842 10.1021/jacs.6b05398

[20] X. Chang , Z. J. Zhao , Z. Lu , S. Chen , R. Luo , S. Zha , L. Li , G. Sun , C. Pei , J. Gong , Nat. Nanotechnol. 2023, 18, 611. 36973396 10.1038/s41565-023-01344-z

[21] J. Suntivich , H. A. Gasteiger , N. Yabuuchi , H. Nakanishi , J. B. Goodenough , Y. Shao‐Horn , Nat. Chem. 2011, 3, 546. 21697876 10.1038/nchem.1069

[22] Y. Sun , H. Liao , J. Wang , Bo Chen , S. Sun , S. J. H. Ong , S. Xi , C. Diao , Y. Du , J‐Ou Wang , M. B. H. Breese , S. Li , H. Zhang , Z. J. Xu , Nat. Catal. 2020, 3, 554.

[23] L. Wu , T. Guo , T. Li , Adv. Funct. Mater. 2022, 32, 2203439.

[24] C. Jia , Q. Wang , J. Yang , Ke Ye , X. Li , W. Zhong , H. Shen , E. Sharman , Yi Luo , J. Jiang , ACS Catal. 2022, 12, 3420.

[25] Z. K. Han , D. Sarker , R. Ouyang , A. Mazheika , Y. Gao , S. V. Levchenko , Nat. Commun. 2021, 12, 1833. 33758170 10.1038/s41467-021-22048-9 PMC7988173

[26] C. Ren , S. Lu , Y. Wu , Y. Ouyang , Y. Zhang , Q. Li , C. Ling , J. Wang , J. Am. Chem. Soc. 2022, 144, 12874. 35700099 10.1021/jacs.2c04540

[27] Qi Zhang , P‐Ao Hu , Z. Y. Xu , B. B. Tang , H‐Ru Zhang , Yu‐H Xiao , Yu‐C Wu , Nanoscale 2023, 15, 4991. 36786677 10.1039/d2nr07301c

[28] J. Wang , M. Zheng , X. Zhao , W. Fan , ACS Catal. 2022, 12, 5441.

[29] C. Fang , J. Zhou , L. Zhang , W. Wan , Y. Ding , X. Sun , Nat. Commun. 2023, 14, 4449. 37488102 10.1038/s41467-023-40177-1 PMC10366111

[30] S. K. Kaiser , E. Fako , I. Surin , F. Krumeich , V. A. Kondratenko , E. V. Kondratenko , A. H. Clark , N. López , J. Pérez‐Ramírez , Nat. Nanotechnol. 2022, 17, 606. 35484211 10.1038/s41565-022-01105-4

[31] D. Li , H. Xu , J. Zhu , D. Cao , J. Mater. Chem. A 2022, 10, 1451.

[32] H. Yuan , Z. Li , X. C. Zeng , J. Yang , J. Phys. Chem. Lett. 2020, 11, 3481. 32298119 10.1021/acs.jpclett.0c00676

[33] X. Chang , Z. J. Zhao , Z. Lu , S. Chen , R. Luo , S. Zha , L. Li , G. Sun , C. Pei , J. Gong , Nat. Nanotechnol. 2023, 18, 611. 36973396 10.1038/s41565-023-01344-z

[34] X. Chang , Z. Lu , R. Luo , et al., Chem 2024, 11, 102294.

[35] L. S. Panchakarla , K. S. Subrahmanyam , S. K. Saha , A. Govindaraj , H. R. Krishnamurthy , U. V. Waghmare , C. N. R. Rao , Adv. Mater. 2009, 21, 4726.

[36] A. VahidMohammadi , J. Rosen , Y. Gogotsi , Science 2021, 372, abf1581. 10.1126/science.abf1581 34112665

[37] H. C. Zhou , S. Kitagawa , Chem. Soc. Rev. 2014, 43, 5415. 25011480 10.1039/c4cs90059f

[38] W. Zhang , Y. Chao , W. Zhang , J. Zhou , F. Lv , K. Wang , F. Lin , H. Luo , J. Li , M. Tong , E. Wang , S. Guo , Adv. Mater. 2021, 33, 2102576. 10.1002/adma.202102576 34296795

[39] J. Wang , C. X. Zhao , J. N. Liu , Y. W. Song , J. Q. Huang , B. Q. Li , Nano Energy 2022, 104, 107927.

[40] T. Pu , J. Ding , F. Zhang , et al., Angew. Chem., Int. Ed. 2023, 62, 202305964. 10.1002/anie.202305964 37277990

[41] S. Ning , H. Ou , Y. Li , C. Lv , S. Wang , D. Wang , J. Ye , Angew. Chem., Int. Ed. 2023, 62, 202302253. 10.1002/anie.202302253 37012479

[42] H. Li , X. Li , P. Wang , Z. Zhang , K. Davey , J. Q. Shi , S. Z. Qiao , J. Am. Chem. Soc. 2024, 146, 22850. 39096280 10.1021/jacs.4c09079

[43] J. Jiménez‐Luna , F. Grisoni , G. Schneider , Nature Machine Intelligence 2020, 2, 573.

[44] J. T. Townsend , Perception & Psychophysics 1971, 9, 40.

[45] H. Chen , L. Wang , D. Long , Y. Zeng , S. Jiang , W. Chen , C. Zhao , C. Cheng , Y. Chen , M. Lu , S. Li , X. Chen , Applied Catalysis B: Environment and Energy 2024, 357, 124260.

[46] F. Abild‐Pedersen , J. Greeley , F. Studt , J. Rossmeisl , T. R. Munter , P. G. Moses , E. Skúlason , T. Bligaard , J. K. Nørskov , Phys. Rev. Lett. 2007, 99, 016105. 17678168 10.1103/PhysRevLett.99.016105

[47] Z. Luo , Li Li , V. T. Nguyen , U. Kanbur , Y. Li , J. Zhang , R. Nie , A. Biswas , S. L. Bud'ko , J. Oh , L. Zhou , W. Huang , A. D. Sadow , B. Wang , S. L. Scott , L. Qi , J. Am. Chem. Soc. 2024, 146, 8618. 38471106 10.1021/jacs.4c00741

[48] M. Newville , Rev. Mineral. Geochem. 2014, 78, 33.

[49] M. Liu , Y. Li , L. Yang , et al., Angew. Chem., Int. Ed. 2025, 64, 202505268. 10.1002/anie.202505268 40326345

[50] P. Tian , G. Zhan , J. Tian , et al., Appl. Catal., B 2022, 315.

[51] X. Liu , M. Wang , H. Yin , J. Hu , K. Cheng , J. Kang , Q. Zhang , Ye Wang , ACS Catal. 2020, 10, 8303.

[52] Z. Li , J. Wang , Y. Qu , H. Liu , C. Tang , S. Miao , Z. Feng , H. An , C. Li , ACS Catal. 2017, 7, 8544.

[53] Y. Ni , Z. Chen , Y. Fu , Y. Liu , W. Zhu , Z. Liu , Nat. Commun. 2018, 9, 3457. 30150779 10.1038/s41467-018-05880-4 PMC6110867

[54] Z. Li , Y. Qu , J. Wang , H. Liu , M. Li , S. Miao , C. Li , Joule 2019, 3, 570.

[55] J. Wei , Q. Ge , R. Yao , et al., Nat. Commun. 2017, 8, 15174. 28462925 10.1038/ncomms15174 PMC5418575

[56] S. Wang , Li Zhang , W. Zhang , P. Wang , Z. Qin , W. Yan , M. Dong , J. Li , J. Wang , L. He , U. Olsbye , W. Fan , Chem 2020, 6, 3344.

[57] S. Chen , J. Wang , Z. Feng , Y. Jiang , H. Hu , Y. Qu , S. Tang , Z. Li , J. Liu , J. Wang , C. Li , Angew. Chem., Int. Ed. 2024, 63, 202316874. 10.1002/anie.202316874 38179842

[58] P. Gao , S. Dang , S. Li , X. Bu , Z. Liu , M. Qiu , C. Yang , H. Wang , L. Zhong , Y. Han , Q. Liu , W. Wei , Y. Sun , ACS Catal. 2017, 8, 571.

[59] P. Gao , S. Li , X. Bu , S. Dang , Z. Liu , H. Wang , L. Zhong , M. Qiu , C. Yang , J. Cai , W. Wei , Y. Sun , Nat. Chem. 2017, 9, 1019. 28937667 10.1038/nchem.2794

[60] S. Dang , P. Gao , Z. Liu , X. Chen , C. Yang , H. Wang , L. Zhong , S. Li , Y. Sun , J. Catal. 2018, 364, 382.

