# Supplementary material for: Universal and Interpretable Descriptor to Design Dual‐atom Catalysts for Multi‐Type C–C Coupling with Ultrahigh C2+ Yield
Source: Adv Sci (Weinh). 2025 Sep 16;12(45):e12614. doi: 10.1002/advs.202512614 (PMC12677638; doi:10.1002/advs.202512614)
Supplement: Supplementary file 1 — Supporting Information [file ADVS-12-e12614-s001.docx]

**Supporting Information**

**Universal and Interpretable Descriptor to Design Dual-atom Catalysts for Multi-Type C–C** **Coupling with Ultrahigh C_2+_ Yield**

Yuming Gao, Chenyi Guo, Juncheng Hong, Haoyu Yan, JinbiaoZhang, Zhe Wang, Dawei Tang, and Bo Jiang*

School of Energy and Power Engineering

Key Laboratory of Ocean Energy Utilization and Energy Conservation of Ministry of Education, Dalian University of Technology

116024, Dalian China

E-mail: bjiang@dlut.edu.cn

# Table of Contents

Table of Contents 2

Computational Methods 5

Density Functional Theory (DFT) Calculation 5

Experimental Procedures 6

Synthesis of ZIF-8 and NC 6

Synthesis of dual-atom catalysts 6

Catalyst Testing 6

Materials Characterization 7

Supplementary Note 1. Input Feature Screening 8

Supplementary Note 2. Machine learning 8

Supplementary Note 3. The limitations of the model in terms of scalability or transferability 10

Supplementary Note 4. Reproducibility of catalyst synthesis 11

Figure S1. Optimized structure of DACs through (a) top view, and (b) side view. The PtMo@DAC is a representative. Blue, Pt; orange, Mo; silver, N; brown, C; white, H. 13

Figure S2. Reaction pathways for several C–C coupling processes in this work. 14

Figure S3. Diverse adsorption configurations of reaction intermediates, CH^*^, CH_2_^*^, CHCH^*^, and CHCH_2_^*^ are considered on the surface of DACs. 15

Figure S4. Diverse adsorption configurations of reaction intermediates, COCO^*^, CO^*^, CH_2_O^*^, CHOCO^*^, and CHO^*^are considered on the surface of DAC. 16

Figure S5. Heat map of the Pearson correlation coefficient matrix between selected features and output values (ΔΔ*G*_selectivity_) in the input feature sets Feature 1 and Feature 2.. 17

Figure S6. Input feature sets screening flowchart. 18

Figure S7. Comparison of the RMSE and the R^2^ score for each model on the train set and the test set. 19

Figure S8. The performance of each chosen model.. 20

Figure S9. GBR Model performance evaluation with 20 distinct partitioning configurations of (a) R^2^, and (b) RMSE score. 21

Figure S10. Heatmap for the prediction results by the GBR model, where the horizontal and vertical axes represent the transition metals TM_1_ and TM_2_, respectively. 22

Figure S11. Feature importance ranking based on (a) GBR model and (b) SHAP analysis. 23

Figure S12. The relationship between ΔΔ*G* selectivity and descriptors composed of different weights, namely, (a) *θ_d_*, (b) min*χ*, (c) Σ*θ_d_* + min*χ* (d) 0.01Σ*θ_d_*+min*χ*. 24

Figure S13. Confusion matrix for the descriptors composed of different weights, namely, (a) *θ_d_*, (b) min*χ*, (c) Σ*θ_d_* + min*χ*, (d) 0.01Σ*θ_d_* + min*χ*.. 25

Figure S14. Relationship between Δ*G*_activity_ and descriptors composed of different weights, namely, (a) *θ_d_*, (b) min*χ*, (c) Σ*θ_d_* + min*χ*, (d) 0.01Σ*θ_d_* + min*χ*.. 26

Figure S15. Calculated kinetic barriers for the CH-CH coupling of (a) NbY@NC, (b) NbSc@NC, (c) TaSc@NC, (d) MnHf@NC, (e) FeZr@NC, (f) VCu@NC, (g) FeCu@NC, (h) PtMo@NC, and (i) CoCu@NC. IS, TS, and FS denote the initial state, transition state, and final state, respectively. 27

Figure S16. Relationship between Δ*G*_activity_ = - ΔE_a_ and *φ*. 28

Figure S17. The Charge difference for (a) C–C coupling and (b) hydrogenation. Charge accumulation is in blue and depletion is in yellow.. 29

Figure S18. Schematic diagram illustrating the selection of C-C coupling or hydrogenation. 30

Figure S19. Charge transfer from CH^*^ versus Δ*G*_activity_. 31

Figure S20. Schematic diagram of the classification and evaluation for descriptors. 32

Figure S21. (a) Zr 3d and (b) Fe 2p XPS spectra of FeZr@NC. 33

Figure S22. The corresponding FT-EXAFS fitting curves of (a) Fe foil and (b) Fe_2_O_3_. 34

Figure S23. The corresponding FT-EXAFS fitting curves of (a) Zr foil and (b) ZrO_2_. 35

Figure S24. Stability test for FeZr@NC catalyst. Reaction conditions: 370 ^o^C, 3 MPa, 12000 mL/g_cat_/h. 36

Figure S25. XRD patterns of the spent FeZr@NC. 37

Figure S26. (a) C 1s, (b) N 1s, (c) Zr 3d, and (d) Fe 2p XPS spectra of the spent FeZr@NC. 38

Figure S27. (a)TEM images and (b) EDS mapping of the spent FeZr@NC. 39

Figure S28. XRD patterns of FeZr@NC of reproducibility experiments. 40

Figure S29. (a) TEM images and (b) EDS mapping for FeZr@NC of reproducibility experiments. 41

Figure S30. CO_2_ conversion, C_2+_ selectivity, and C_2+_ yield of FeZr@NC. Reaction conditions: 370 ^o^C, 3 MPa, 12000 mL/g_cat_/h. Error bars correspond to the standard deviation of three independent measurements. 42

Table S1. The 12 features used in the ML model 43

Table S2. Atomic features obtained from the literature.^[15-18]^ 44

Table S3. Features of different input feature sets. The subscript 1 represents the TM_1_ atom of DACs, while those with subscript 2 represent the TM_2_ atom of DACs 45

Table S4. The specific values of ΔΔ*G*_selectivity_ 46

Table S5. The value of Δ*G*_activity_ (Δ*G*_activity_ = -Δ*G*_coupling_). 49

Table S6. GBR Model performance evaluation with 20 distinct partitioning configurations of R^2^, and RMSE score. 50

Table S7. Bader charge for the coupling and hydrogenation process. 51

Table S8. The values for the difference between transferring electrons to CH^*^ and acquiring electrons from H^*^ of DACs, electron transfer to CH^*^, and the d-band center of active metal sites 52

Table S9. The value of -Δ*G*_coupling_ and ΔΔ*G*_selectivity_ for CO^*^–CO^*^, CHO^*^–CO^*^, and CH^*^–CH_2_^*^ processes 53

Table S10. Detailed hyperparameters for each ML model 54

Table S11. The difference between the descriptors in previous papers and this work. ‘−’ represents that this item cannot be evaluated. 55

Table S12. Chemical composition of the FeZr@CN recorded by XPS 56

Table S13. EXAFS fitting results for different materials at Fe K-edge 57

Table S14. EXAFS fitting results for different materials at Zr K-edge 58

Table S15. Comparison studies on the catalytic performance of various catalysts for CO_2_ into light olefins 59

References 60

# Computational Methods

## Density Functional Theory (DFT) Calculation

Our computational investigations were conducted using the Density Functional Theory (DFT) calculations, facilitated by the Vienna Ab Initio Simulation Package (VASP).^[1]^ The core electrons were described using the Projector Augmented Wave (PAW) method, in conjunction with the Perdew-Burke-Ernzerhof (PBE) exchange-correlation function. The wavefunctions were expanded using a plane-wave basis set, with a kinetic energy cutoff of 400 eV. A gamma-centered k-point mesh was employed, utilizing a 2 × 2 × 1 grid. The Grimme’s D3 method was employed to account for the van der Waals interactions.^[2]^ To account for the strong Coulomb interactions among the electrons of transition metals, we implemented the DFT + U method.^[3]^ The geometric optimization was deemed convergent when the energy change was less than 0.02 eV Å^-1^. Furthermore, the electron energy was considered self-consistent only when the energy change was less than 10^-5^ eV. The energy of the optimized structures was obtained when both the energy and force simultaneously reached the expected outcome. The thickness of the vacuum layer, perpendicular to the surface of the structure, was set at 15 Å. To comprehend the charge transfer between the active site and the adsorbed intermediates, we calculated the Bader charge and visualized it using the charge density difference. The Gibbs free energies of each intermediate were computed at 573.15 K, as per the following equation:^[4-5]^

$G=E_{DFT}+E_{ZPE}-TS$ (1)

In this equation, *E_DFT_* represents the electronic energy, while *E_ZPE_* and *TS* denote the zero-point energy and the entropy contribution, respectively. These values were derived from the vibrational frequencies computed using the VASPKIT code.^[6]^ The reaction energies, denoted as *G*, are defined by the equation Δ*G = G _final_* - *G _initial_*, where *G _final_* and *G _initial_* represent the total energy of the adsorbed intermediates after and before the reaction, respectively. This provides a comprehensive understanding of the energy changes during the reaction process. Further, the CI-NEB method was utilized to explore the transition state characteristics of the catalysts.^[7]^

As for the selectivity prediction toward C_2+_, we utilized the thermodynamic descriptor ΔΔ*G*_selectivity_. The ΔΔ*G*_selectivity_ value is calculated as the difference between G_1_ and G_2_, which represent the free energy differences between the C-C coupling step and the hydrogenation step, according to the following equation:

$G_{1}=\Delta G_{coupling}$ (2)

$G_{2}=\Delta G_{hydrogenation}$ (3)

$\Delta\Delta G_{selectivity}=\Delta G_{coupling}-\Delta G_{hydrogenation}$ (4)

# Experimental Procedures

## Synthesis of ZIF-8 and NC

To begin with, 2.38 g of Zn(NO_3_)_2_•6H_2_O and 2.63 g of 2-methylimidazole were dissolved in 75 mL and 45 mL of methanol, respectively, and then mixed at room temperature while stirring continuously. After stirring at 350 rpm for 12 h, the ZIF-8 was obtained through centrifugation. After obtaining the precipitates, they were centrifuged and thoroughly washed with methanol multiple times, then dried in a vacuum at 60℃ overnight. Finally, the sample was placed in a tube furnace and heated to 1000℃ for 2 h in an argon stream to produce NC.

## Synthesis of dual-atom catalysts

40 mg NC was added to 25 mL of ethanol and dispersed by ultrasound to obtain a black solution. Then, the mixed ethanol solution of two metal salts, both having the same concentration, was added dropwise into the black solution under stirring at 80 °C for 6 h. After the product cooled to room temperature, the product was subjected to centrifugation and subsequently dried in a vacuum at 60 °C overnight. The as-obtained black powder was heated at 800 °C for 1 h with a heating rate of 5 °C min^-1^ in an Ar atmosphere, yielding different combinations of catalysts.

## Catalyst Testing

CO₂ hydrogenation catalytic reaction was systematically evaluated in a high-pressure tubular fixed-bed reactor. Typically, 0.1 g of catalyst was first pretreated under a 30 mL/min argon flow at 360°C and atmospheric pressure for 1 hour. Subsequently, a reactant mixture with CO₂/H₂/Ar volume ratio of 1:3:6 (total flow rate 20 mL/min) was introduced into the reactor at 360°C, and 3.0 MPa pressure, achieving a gas hourly space velocity (GHSV) of 12,000 mL/g_cat_/h. The gaseous products were analyzed online using a gas chromatography system equipped with both a thermal conductivity detector (TCD) and a flame ionization detector (FID) for real-time compositional analysis. CO_2_ conversion (denoted as X (CO_2_)) was calculated based on the carbon atom (carbon balances were all better than 95%) according to the following equation

$X_{CO_{2}}=\frac{CO_{2in}-CO_{2out}}{CO_{2in}}\times100\%$ (5)

where CO_2in_ and CO_2out_ represent CO_2_ at the inlet and outlet amounts (moles), respectively.

The selectivity of the hydrocarbon product (denoted as S(C_n_H_m_)) was obtained according to the following equation:

$S_{C_{n}\text{H}_{m}}=\frac{n\times C_{n}H_{m}}{CO_{2in}-CO_{2out}-CO_{out}}\times100\%$ (6)

where C_n_H_m_ represents the individual hydrocarbon product amounts (moles).

The selectivity of CO was calculated according to the following equation:

$S_{CO}=\frac{CO_{out}}{CO_{2in}-CO_{2out}}\times100\%$ (7)

where CO_out_ represents the CO at the outlet amount (mole).

## Materials Characterization

X-ray diffraction (XRD) analysis was executed with Rigaku MiniFlex 600, employing Cu *Kα* monochromatic radiation (λ = 0.15418 nm). The atomic metal ratio was analyzed via Inductively coupled plasma atomic emission spectrometry (ICP-AES) (Agilent 5110). Electronic structures were investigated via X-ray photoelectron spectroscopy (XPS) (Thermo Scientific ESCALAB 250Xi equipped with a monochromatic Al K_α_ source, allowing for high-resolution scans with minimal step sizes (0.1 eV) and extended dwell intervals). Utilizing the JEOL JEM-ARM200F, we conducted high-angle annular dark-field scanning transmission electron microscopy (HAADF-STEM) along with energy-dispersive spectroscopy (EDS) mapping analyses. The X-ray absorption fine structure spectroscopy (XAFS) measurements were carried out at the Shanghai Synchrotron Radiation Center at beamline BL14W and beamline BL11B, with X-ray absorption near-edge structure (XANES) and extended X-ray absorption fine structure (EXAFS) recorded in fluorescence mode for the Fe and Zr K edges. The radiation was refined to a single wavelength using a Si (111) double-crystal monochromator, and the data analysis was conducted following established protocols with the Athena software package. Calibrations and structural refinements were carried out using the corresponding pure metal as a reference. The XANES and EXAFS data obtained were analyzed using Athena (version 0.9.26) to perform calibrations for background, pre-edge line, and post-edge line. Subsequently, Fourier transformation fitting was executed in Artemis (version 0.9.26).

# Supplementary Note 1. Input Feature Screening

During the feature selection process, adherence to these strategies is imperative: Firstly, the features ought to be inherent and directly retrievable from the database, circumventing the necessity for DFT computations. For features necessitating DFT calculations, such as the adsorption energy of intermediates, distances, and angles in optimized structures, selecting these features would inhibit the direct prediction of uncalculated results using data from the database via the ML process. This would render large-scale direct prediction processes unfeasible, leading to a forfeiture of practicality and universality. Furthermore, the selection of inherent features as input features also augments the construction of subsequent intrinsic descriptors, serving as integral components of the descriptors. Secondly, certain features should be capable of reflecting the interactions between metals or between metal and ligand atoms. For instance, electronegativity, which represents the attraction of metal or ligand atoms to electrons, influences the redistribution of electrons, thereby modulating the adsorption status of intermediates and either restricting or promoting the progress of the reaction. Analogously, the number of electrons can also wield a potent control over the reaction. Different quantities of electrons imply varying possibilities of gaining or losing, which is also a crucial aspect of influencing the reaction. Thirdly, for disparate catalysts, the chosen features should ideally exhibit a sufficiently large difference, such as the different masses possessed by distinct atoms. If the difference is absent or if the difference is minuscule, it will impinge on the accuracy of the ML process. Ultimately, the features should be physically intuitive. If the input features are excessively complex, such as multifarious mathematical combinations of multiple properties, it will ultimately render the elucidation of catalyst performance through structure-performance relationships arduous, which is not conducive to rationally unveiling the origin of catalytic performance.

# Supplementary Note 2. Machine learning

The machine learning (ML) methodology was employed, underpinned by DFT computational data, and carried out utilizing the scikit-learn package in the Python 3.8.8 environment.^[8]^ The dataset was split into a training set and a test set at a ratio of 3:1. The evaluation criteria for the model adopted the R^2^ value and root-mean-squared error (RMSE).^[9]^ RMSE represents the average error of the model, while R^2^ represents the accuracy of the model. R^2^ and RMSE are calculated as follows:

$R^{2}=1-\frac{\sum_{\text{i}} (Y_{i}-y_{i})^{2}}{\sum_{\text{i}} (Y_{i}-\mathop{Y}\limits^{\_} )^{2}}$ (8)

$RMSE=\sqrt{\frac{1}{N}\sum_{i}^{n} (Y_{i}-y_{i})^{2}}$ (9)

where Y_i_ and y_i_ indicate, respectively, the values obtained from DFT computations and predicted via ML. $\bar{Y}$ represents the average value obtained from DFT data. Each model repeatedly adjusts appropriate hyperparameters using the Grid Search CV function in the scikit-learn package to achieve the highest accuracy (lowest RMSE and highest R^2^ score), thereby enhancing the model’s generalization ability. Based on the reported selection ratio,^[10-15]^ we randomly selected 68 samples, accounting for approximately 17% of the total, which is sufficient to predict over 400 catalysts. Furthermore, we evaluated 68 samples using ten-fold cross-validation with varying partitioning configurations of the training set and test set to ensure the quality of the input dataset. The results, derived from 20 distinct partitioning configurations, demonstrated stable performance metrics (mean R^2^ > 0.98, RMSE <0.09), indicating insensitivity to data splitting and confirming the high quality of the input dataset (Figure S9 and Table S6). Primary hyperparameters for each model are shown in Table S10. During this process, 10-fold cross-validation is used to test the model on the pre-split validation set to ensure that no severe overfitting is observed. After reasonably selecting the hyperparameters of each model, corresponding regression models are constructed for them. Given that the test set was not previously trained, it is more capable of showcasing the model's quality compared with the training set. Based on these principles, we carried out the model screening process. The GBR model performed exceptionally well, with an RMSE of only 0.065 eV in the training set and an R^2^ score of 0.991. The R^2^ and RMSE of other models in the training and test sets were significantly different from the GBR model. Although the ETR model performed well in the training set (R^2^ = 0.963, RMSE = 0.177), its performance in the test set was quite poor (R^2^ = 0.203, RMSE = 0.846), indicating the occurrence of overfitting, and the DTR model exhibited similar behavior.

According to Figure 2e, minχ plays a crucial role in determining ΔΔ*G*_selectivity_; lowering minχ leads to a decrease in ΔΔ*G*_selectivity_, while raising minχ results in an increase. As for maxrM, irrespective of being large or small, it has a beneficial impact on the final output value ΔΔ*G*_selectivity_, indicating that its absolute SHAP value is significant, which reflects a high level of feature importance. Nevertheless, owing to its lack of ability to show differences in output values, maxrM was likely to be seen as irrelevant for selectivity. In terms of *θ_d_.1* + *θ_d_.2* (Σ*θ_d_*), its influence on the output value aligns with minχ. Consequently, Σ*θ_d_* and min*χ* were considered as potential foundational elements of the descriptor, offering crucial insights for future descriptor development. Besides, the classification of ΔΔ*G*_selectivity_ as favorable or unfavorable depended on whether its values were negative or positive, respectively. Therefore, we only predict the quality of selectivity when the descriptor exceeds or falls below a certain threshold, rather than focusing solely on the actual ΔΔ*G*_selectivity_ value.

# Supplementary Note 3. The limitations of the model in terms of scalability or transferability

The ML model we constructed is a regression model based on Gradient Boosting Regression (GBR), and the ML model is capable of accurately assessing key properties such as electronic structure, thermodynamic stability, and adsorption energy, providing crucial physical and chemical interpretability for identifying promising catalyst candidates. Nevertheless, the limitations of our model in terms of scalability and transferability are primarily reflected in two aspects. On the one hand, the ML models are highly dependent on features. If transferred to other systems with shared key physical mechanisms, new models can be quickly created through feature importance transfer. However, if extended to catalytic systems without shared physical mechanisms, such as those where the distribution of key features exceeds the training range of the dual-atom catalysts (DACs) system, the predictive performance of the model will decline. On the other hand, as our current ML model is based on the DACs system, when extending it to other catalytic material systems, such as high-entropy alloy catalysts with complex synergistic properties, the vast number of possible feature combinations may lead to a “combinatorial explosion,” making the method computationally intractable.

To address the limitations of both aspects and further improve the scalability and transferability of our ML models, we can combine GBR models with deep learning (DL). Specifically, the GBR model trained on a small number of high-fidelity DFT data points achieved robust adsorption energy predictions and maintained interpretability through physics-based features (e.g., electronegativity, d electron number). Then, the GBR model was pseudo-labeled for additional structures to create a substantially expanded dataset, mitigating data scarcity without compromising accuracy. Next, DL models (e.g., VAEs) utilize this dataset for inverse design, enabling scalable exploration of materials space. The combination ensures model transferability across various complex systems through a pseudo-labeling method that leverages simple features, avoiding the combination explosion while maintaining DFT-level reliability. Additionally, the physics-based GBR provides mechanistic insights that pure DL methods lack for catalyst design. This synergistic strategy thus achieves a balance between interpretability, scalability, and cross-domain applicability, thereby establishing a scalable artificial intelligence workflow for catalyst design.

# Supplementary Note 4. Reproducibility of catalyst synthesis

To verify the reproducibility of the FeZr@NC with outstanding catalytic performance, we have carried out reproducibility experiments. **On the one hand,** we performed characterization verification. The X-ray diffraction pattern of FeZr@NC presented two broad peaks located at 25^o^ and 44^o^ (Figure S28), which were associated with the characteristic carbon (0 0 2) and (1 0 0) diffractions, respectively, with no identifiable diffraction peaks for metal particles, consistent with the results obtained from the previously synthesized FeZr@NC samples. Moreover, the morphology of the reproducibility FeZr@NC exhibited a slightly rhombic dodecahedron shape, and no nanoparticles were detected, as displayed in Figure S29, indicating that the morphology is also in line with previously synthesized FeZr@NC. The energy dispersive X-ray spectroscopic (EDS) elemental mapping (Figure S29) indicates that Fe, Zr, and N elements are uniformly distributed on the carbon matrix. **On the other hand,** we conducted repeatability experiments on three different batches of FeZr@NC samples for the CO_2_ hydrogenation reaction. We found that the activity, selectivity, and yield of each catalyst sample exhibited minimal standard deviation across the three different tests, indicating that the activity data in this study exhibit good reproducibility (Figure S30). The above results effectively demonstrate the reproducibility of catalyst synthesis.


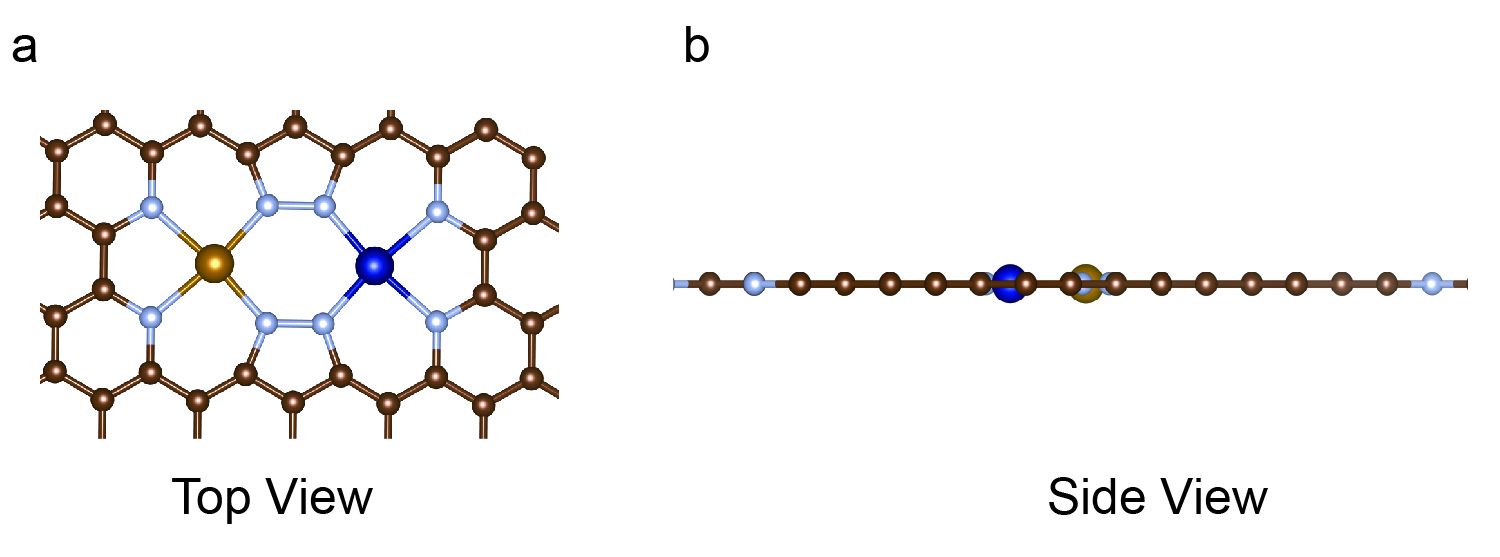


## Figure S1. Optimized structure of DACs through (a) top view, and (b) side view. The PtMo@DAC is a representative. Blue, Pt; orange, Mo; silver, N; brown, C; white, H.


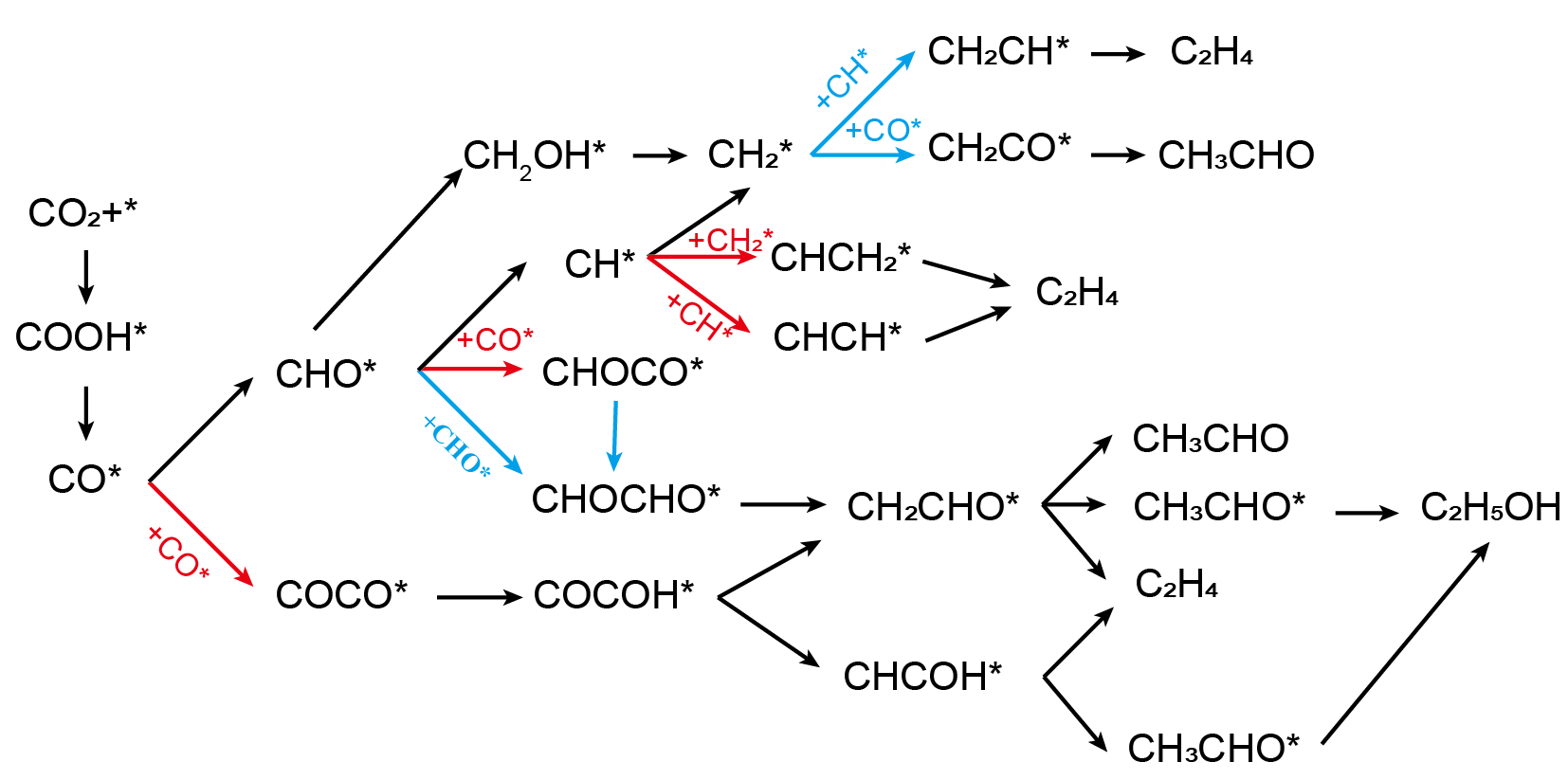


## Figure S2. Reaction pathways for several C–C coupling processes in this work.


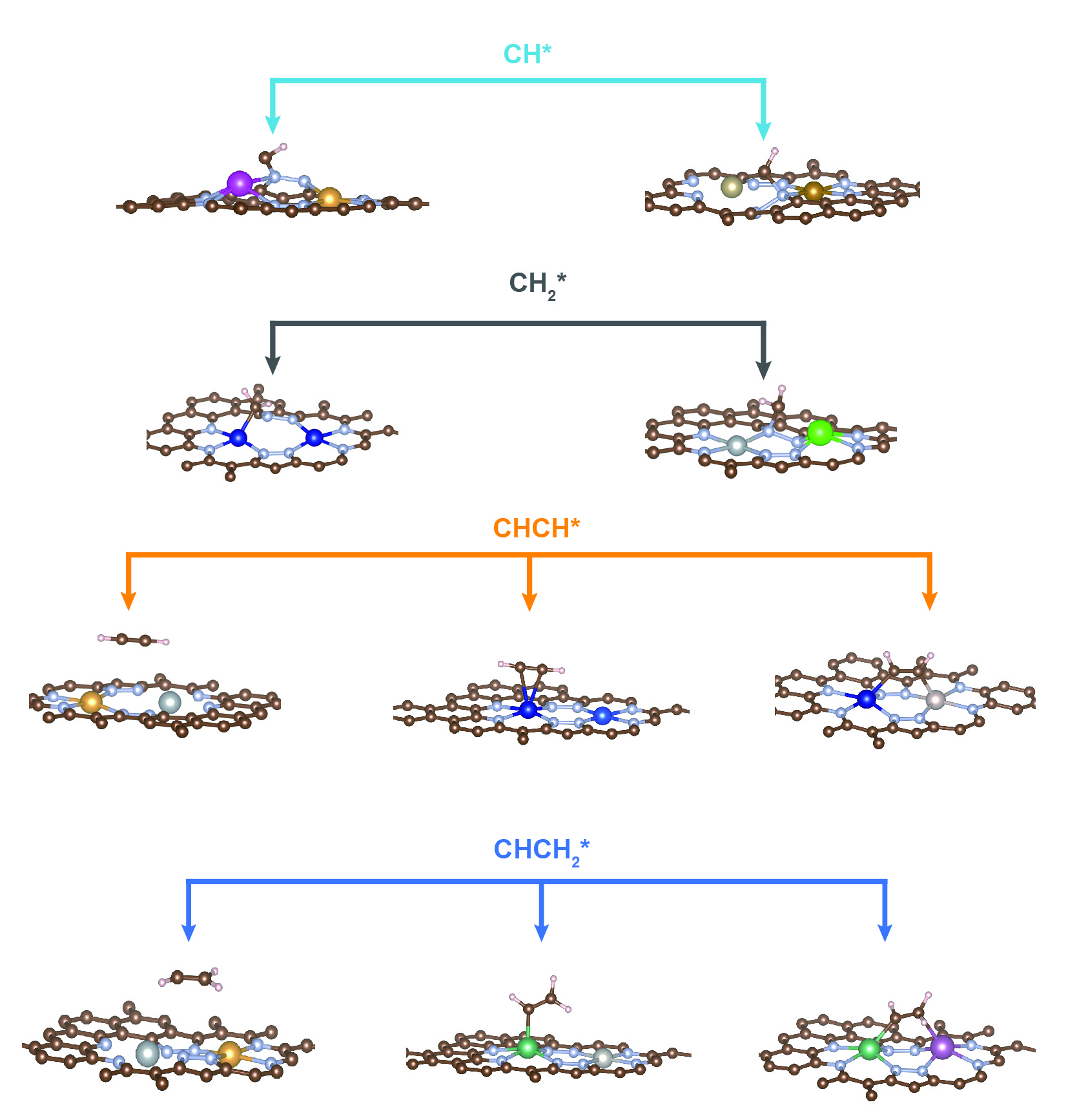


## Figure S3. Diverse adsorption configurations of reaction intermediates, CH^*^, CH_2_^*^, CHCH^*^, and CHCH_2_^*^ are considered on the surface of DACs.


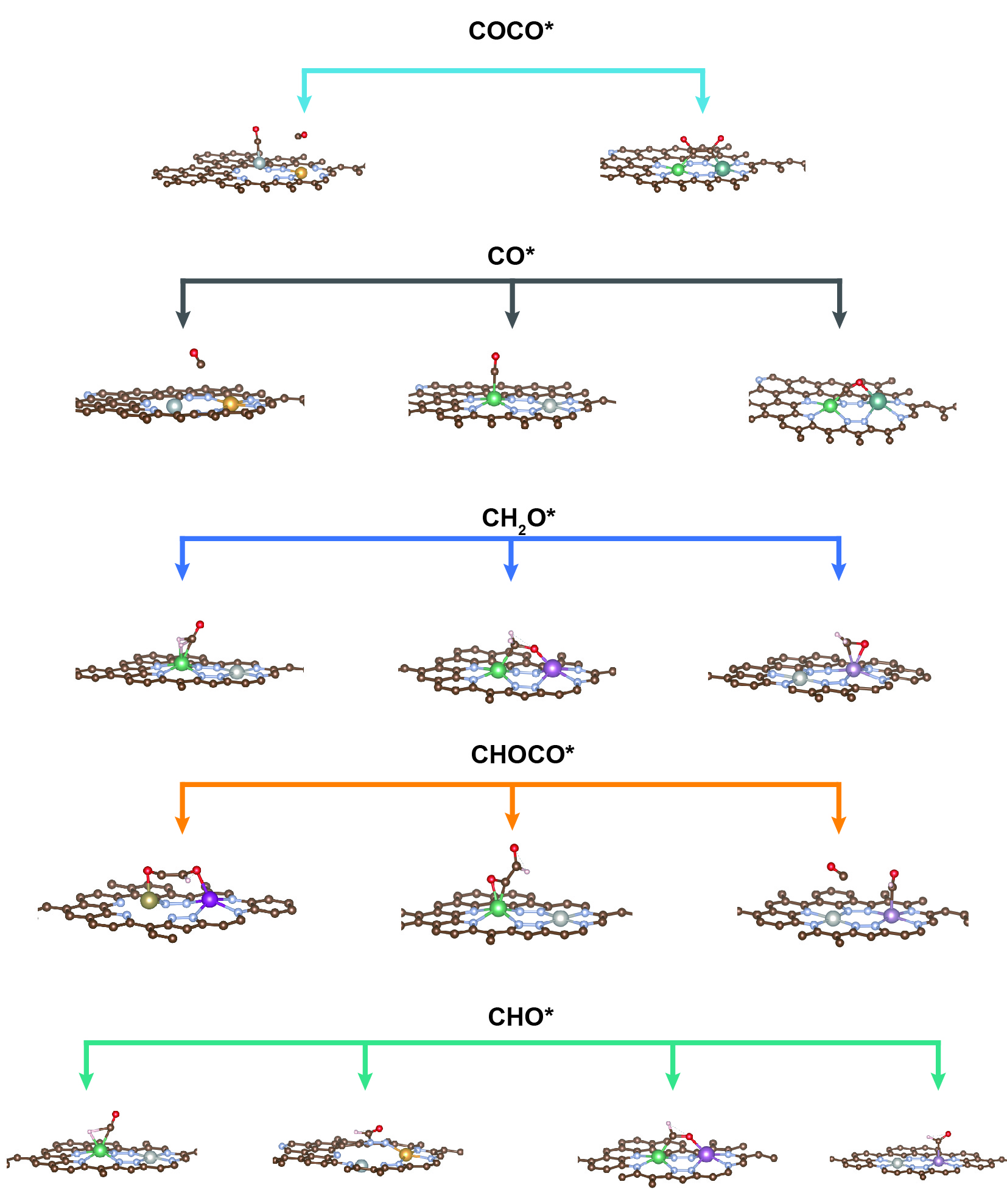


## **Fig**ure **S4.** Diverse adsorption configurations of reaction intermediates, COCO^*^, CO^*^, CH_2_O^*^, CHOCO^*^, and CHO^*^are considered on the surface of DAC.


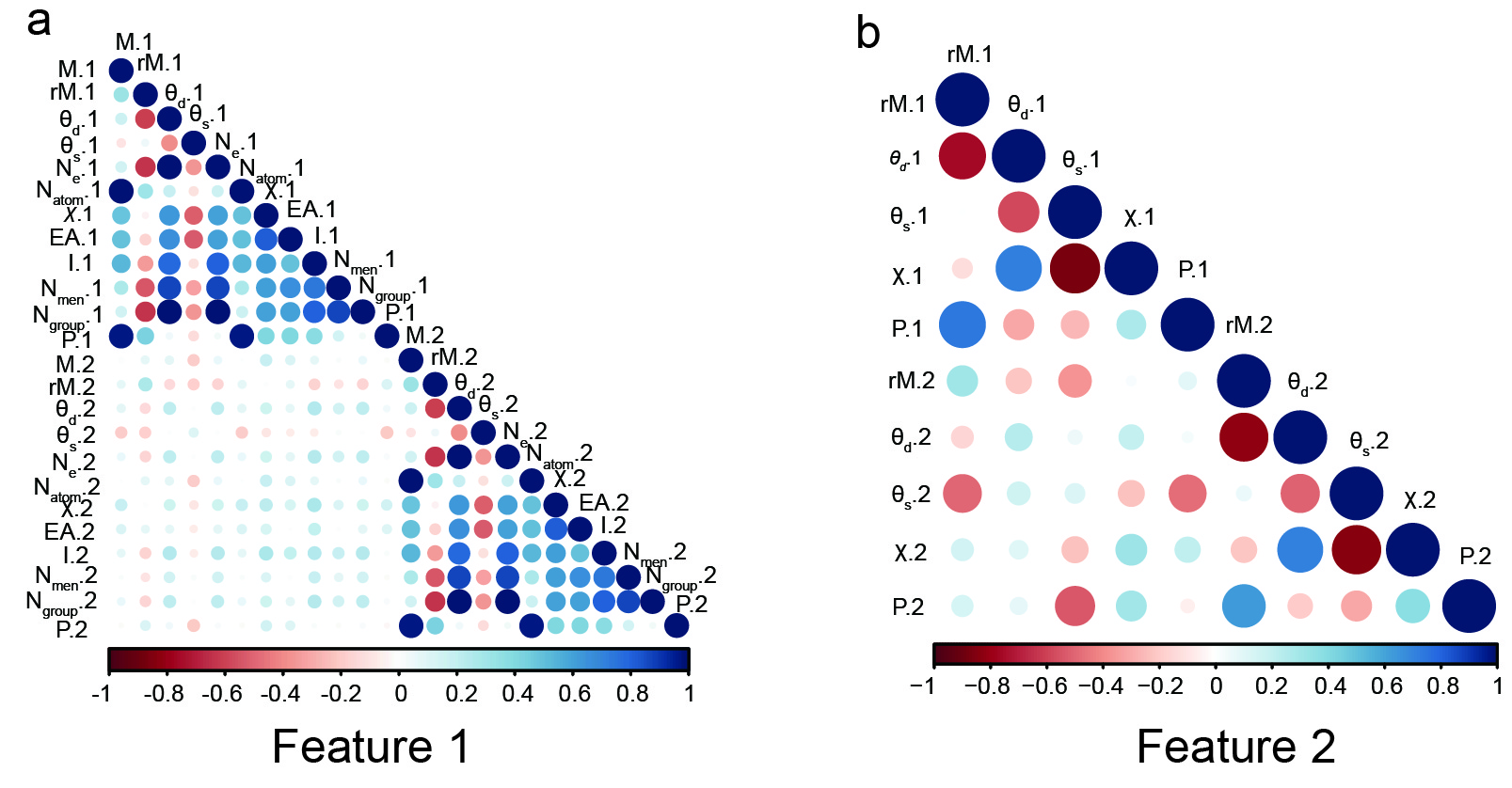


## **Fig**ure **S5.** Heat map of the Pearson correlation coefficient matrix between selected features and output values (ΔΔ*G*_selectivity_) in the input feature sets Feature 1 and Feature 2. The subscript 1 represents the M_1_ atom of DACs, while those with subscript 2 represent the M_2_ atom of DACs.


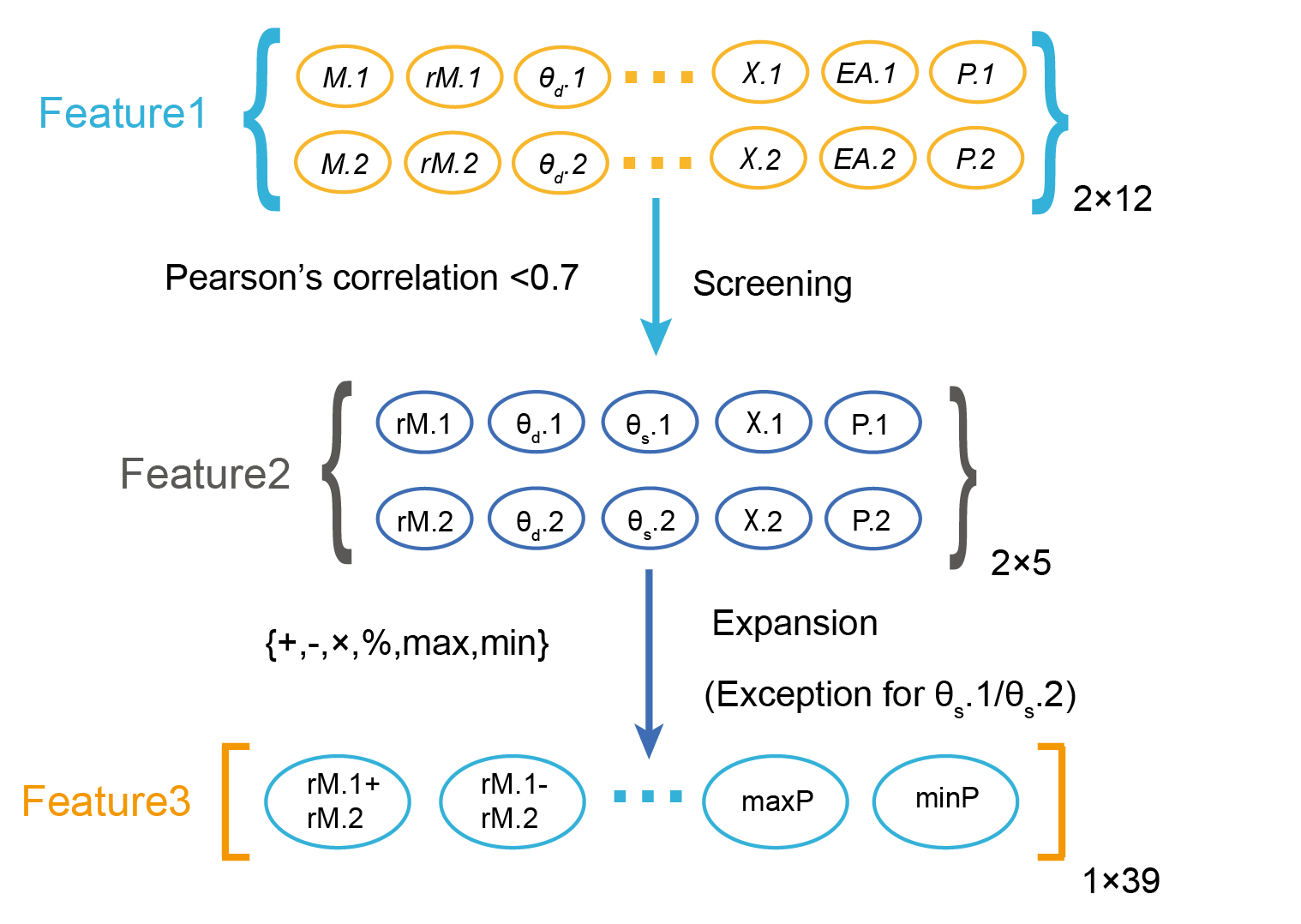


## **Fig**ure **S6.** Input feature sets screening flowchart.


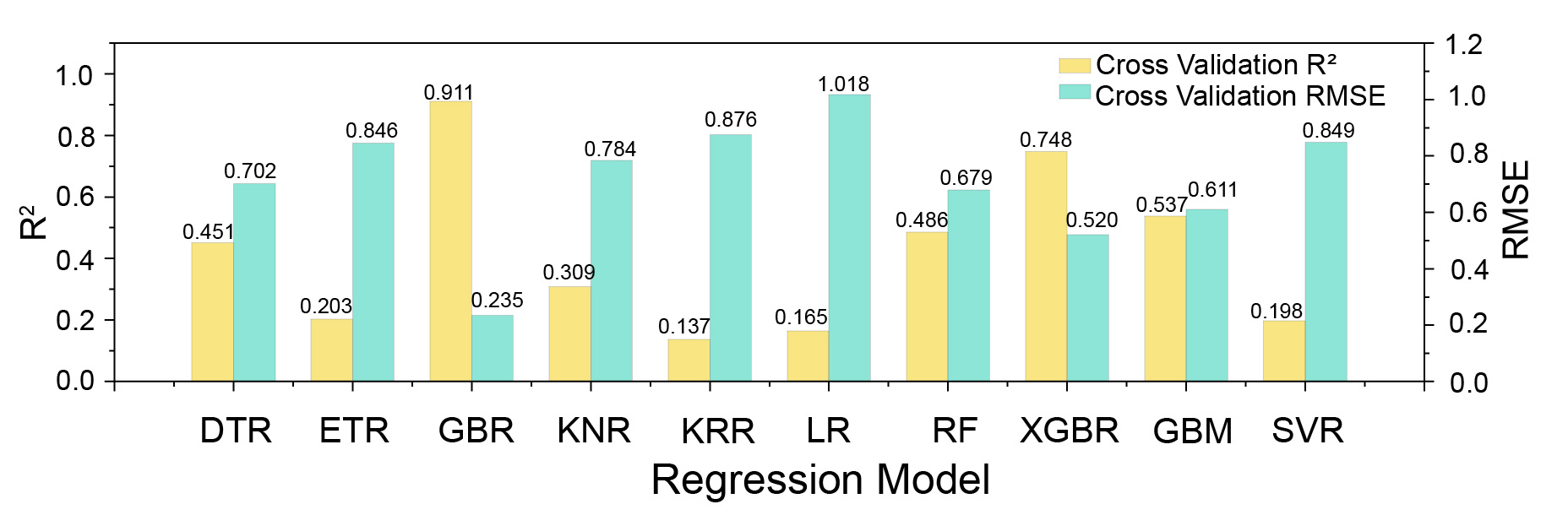


## **Fig**ure **S7.** Comparison of the RMSE and the R^2^ score for each model on the train set and the test set.


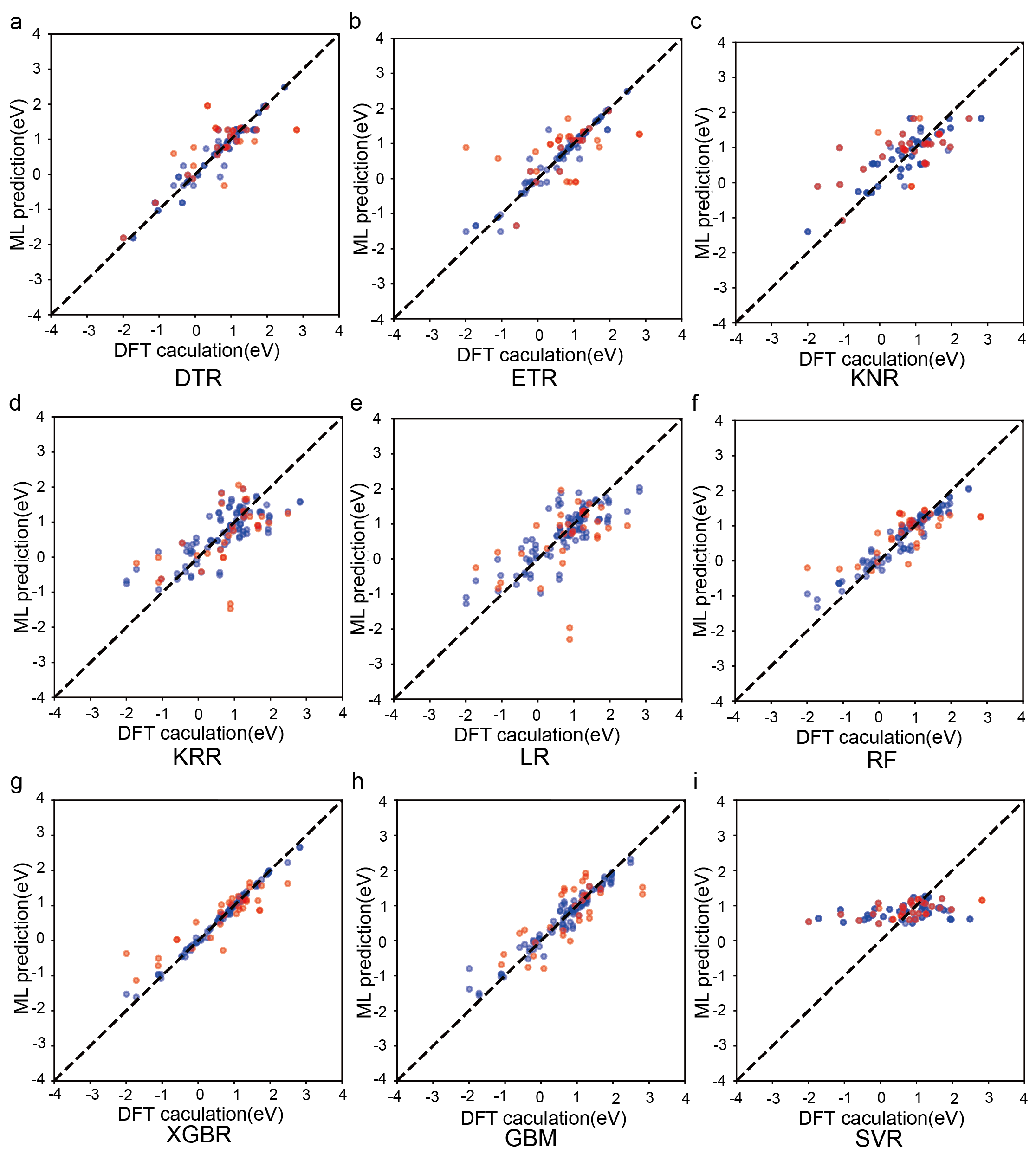


## **Fig**ure **S8.** The performance of each chosen model. The R^2^ and the RMSE score of the training and test sets are shown in the top-left corner. The data points aligned along the diagonal line, which signified a strong linear connection and validated the model's reliability and accuracy of the model.


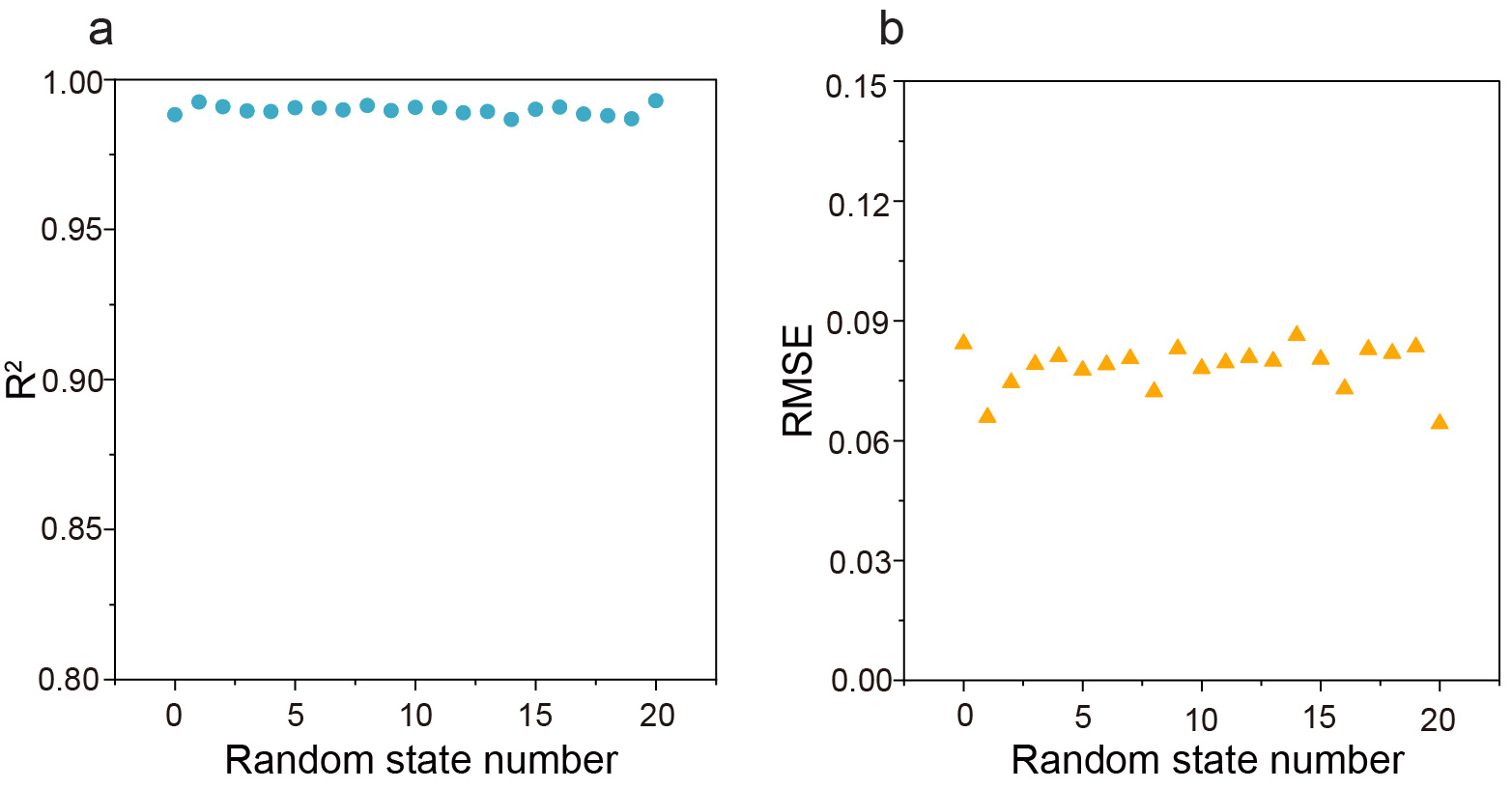


## **Fig**ure **S9.** GBR Model performance evaluation with 20 distinct partitioning configurations of (a) R^2^, and (b) RMSE score.


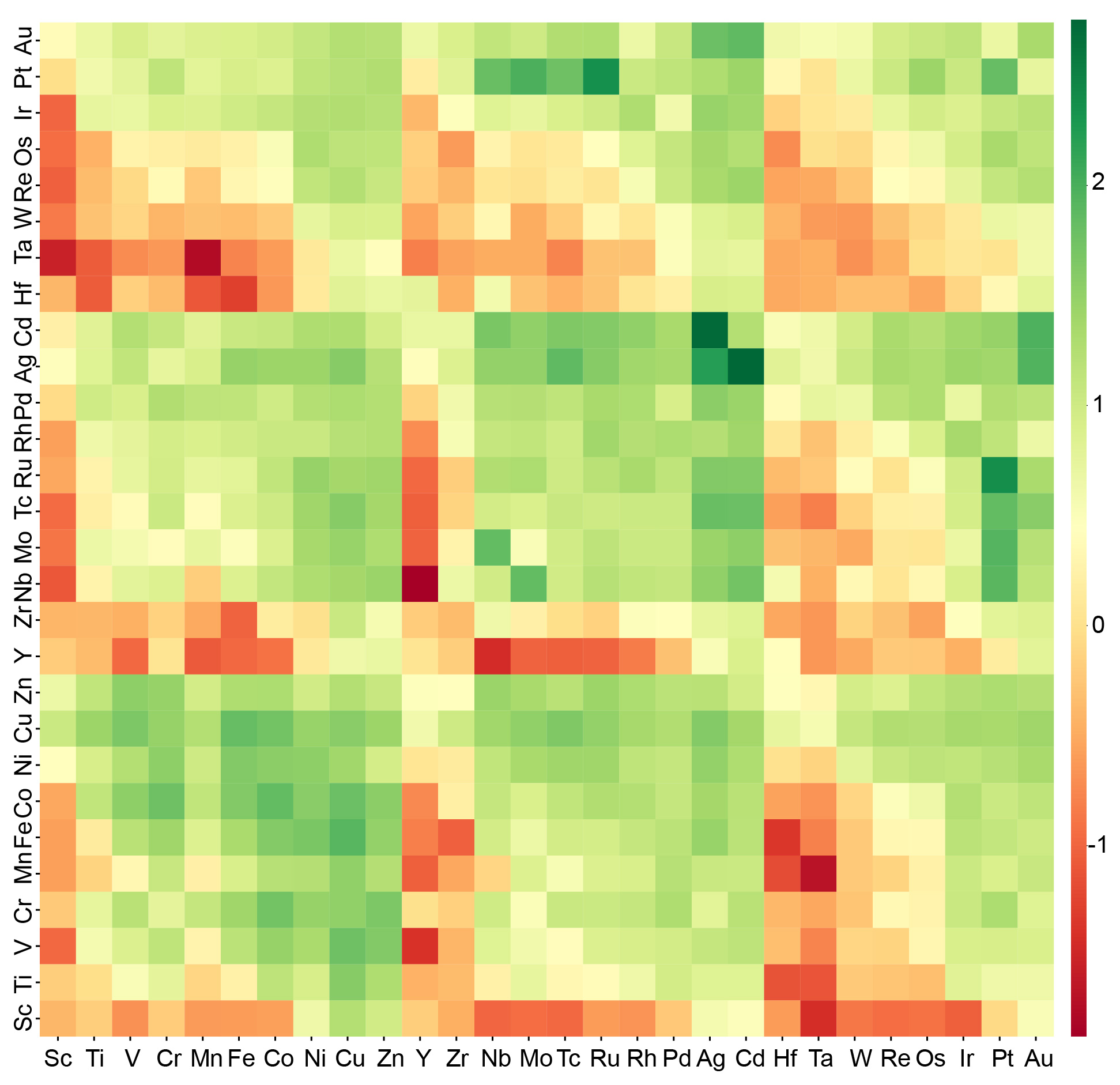


## **Fig**ure **S10.** Heatmap for the prediction results by the GBR model, where the horizontal and vertical axes represent the transition metals TM1 and TM2, respectively.


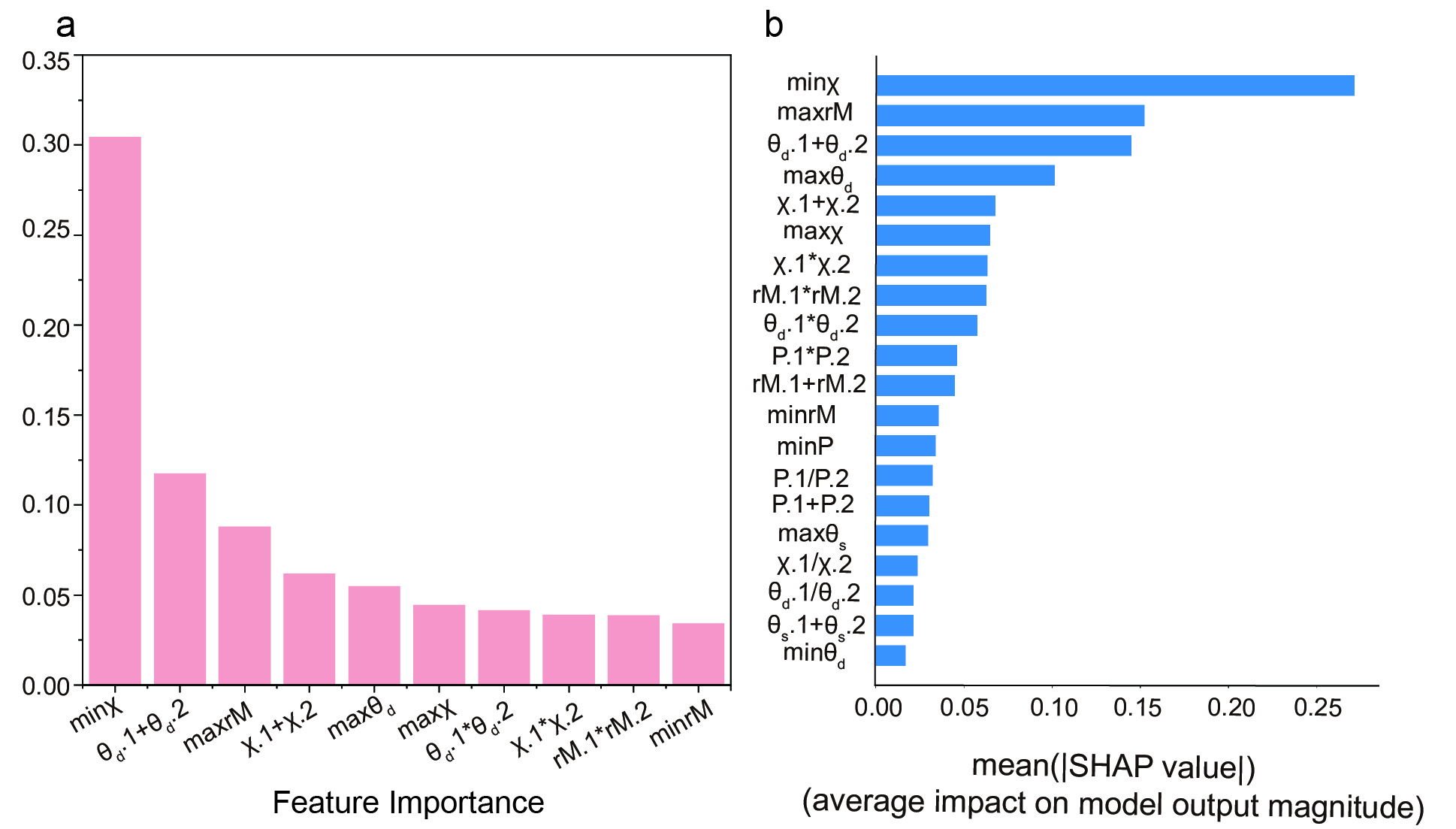


## **Fig**ure **S11.** Feature importance ranking based on (a) GBR model and (b) SHAP analysis.


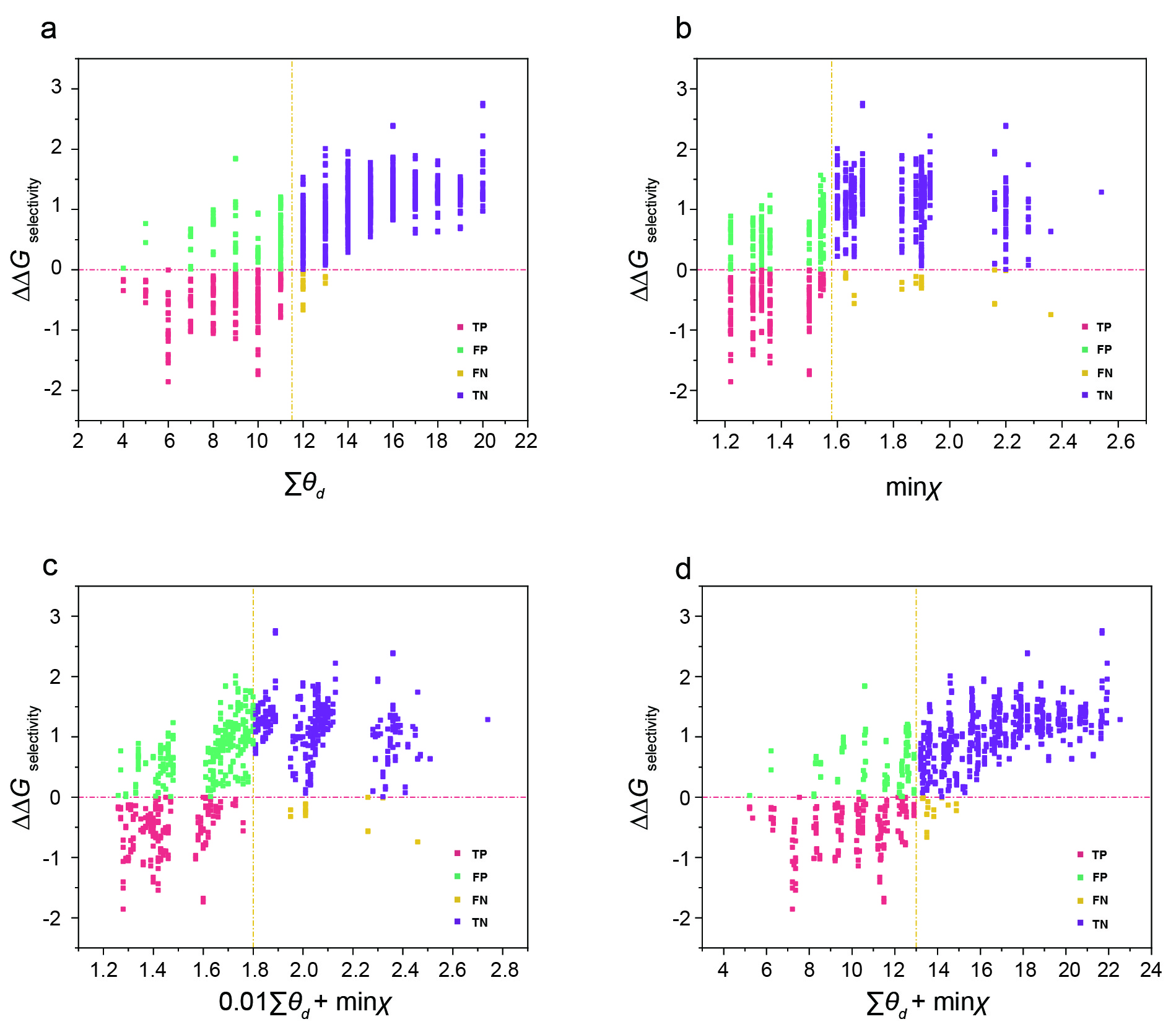


## **Fig**ure **S12.** The relationship between ΔΔ*G* selectivity and descriptors composed of different weights, namely, (a) *θ_d_*, (b) min*χ*, (c) Σ*θ_d_* + min*χ* (d) 0.01Σ*θ_d_*+min*χ*. The red dotted line is the dividing line between high and low selectivity, while the yellow one is predicted by the descriptor *φ*. True positive (TP) was when a high forecast corresponded with a high reality; false positive (FP) was when a high forecast was contradicted by a low reality; false negative (FN) taken place when a low forecast was followed by a high reality; and true negative (TN) is when both the forecast and the reality are low.


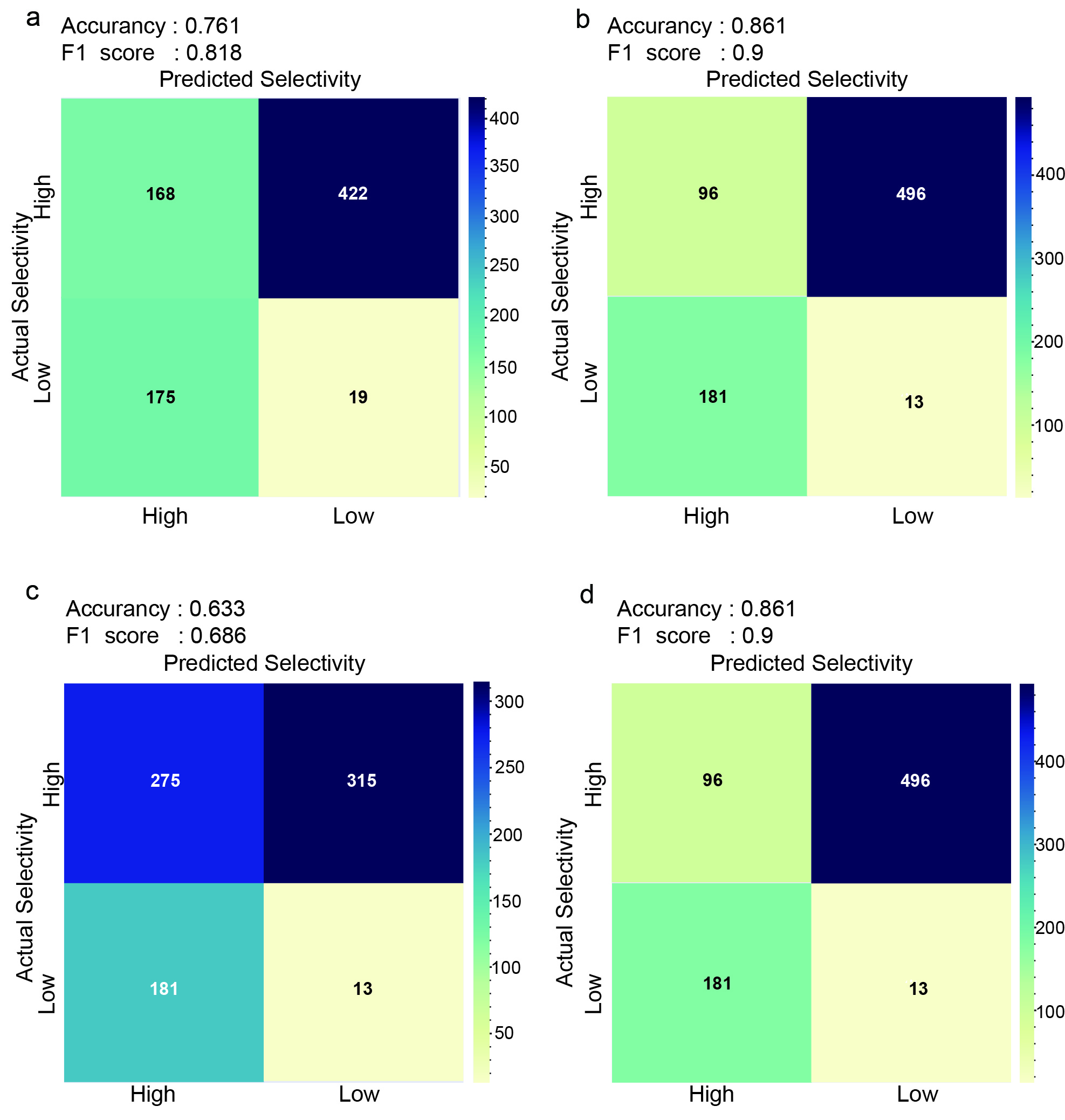


## **Fig**ure **S13.** Confusion matrix for the descriptors composed of different weights, namely, (a) *θ_d_*, (b) min*χ*, (c) Σ*θ_d_* + min*χ*, (d) 0.01Σ*θ_d_* + min*χ*. The matrix is organized with rows that reflect the actual category, columns that represent the predicted category, and each cell holds the number of samples that correspond to the respective actual and predicted category.


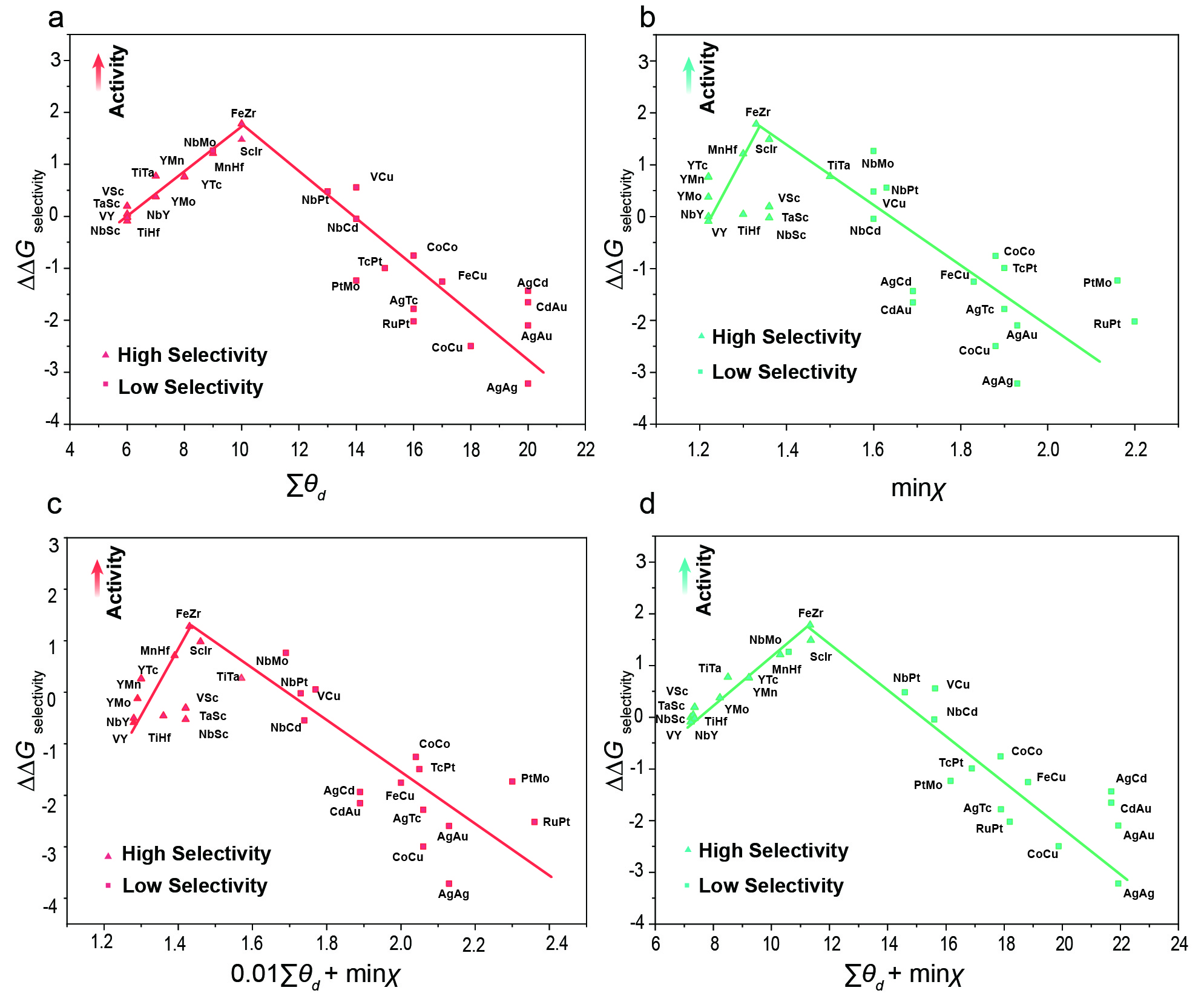


## **Fig**ure **S14.** Relationship between Δ*G*_activity_ and descriptors composed of different weights, namely, (a) *θ_d_*, (b) min*χ*, (c) Σ*θ_d_* + min*χ*, (d) 0.01Σ*θ_d_* + min*χ*. The straight line is formed by the linear fitting of scattered points. Different shapes represent different selectivity. Triangle, high selectivity; square, low selectivity.


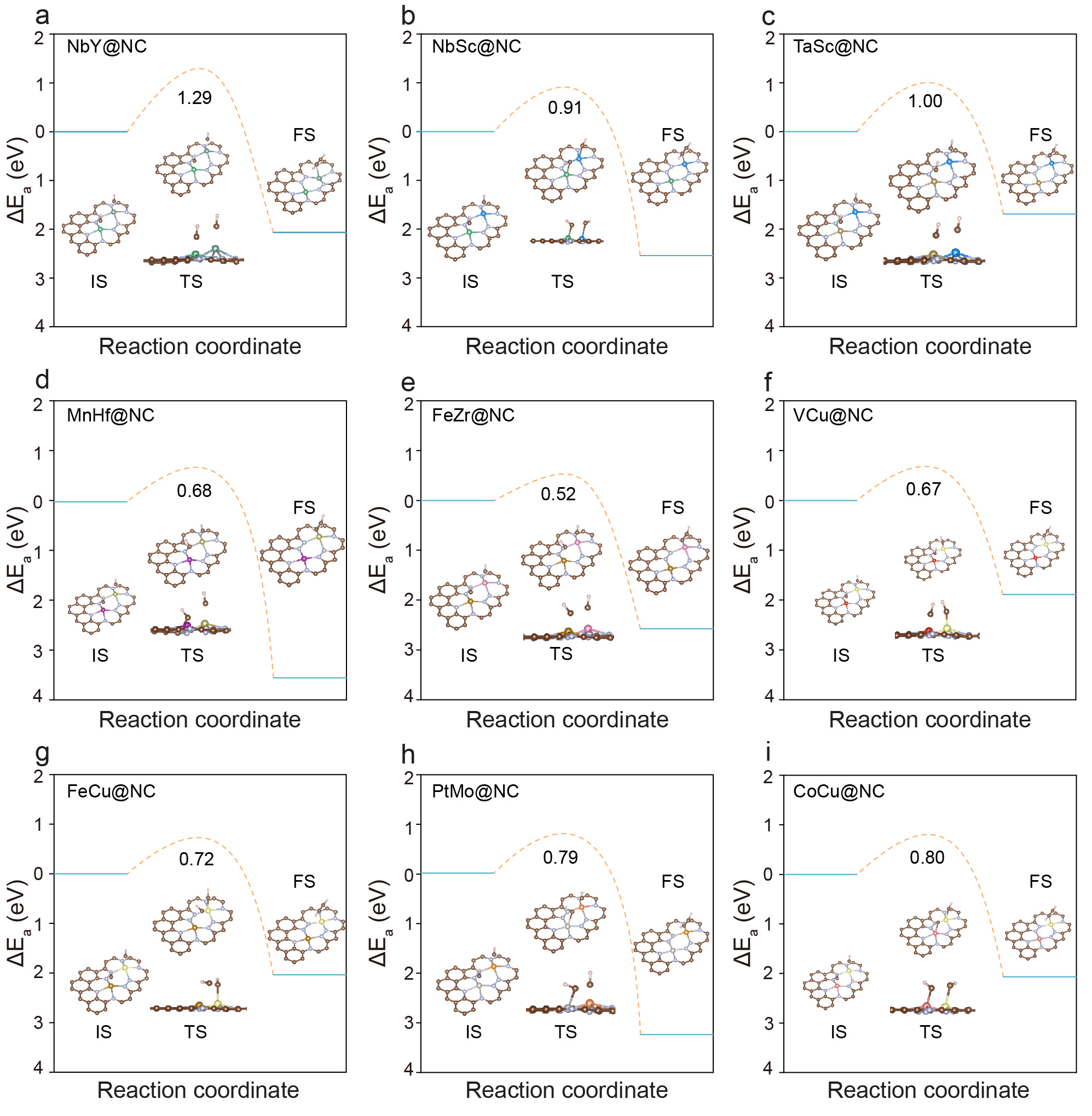


## **Figure S15.** Calculated kinetic barriers for the CH-CH coupling of (a) NbY@NC, (b) NbSc@NC, (c) TaSc@NC, (d) MnHf@NC, (e) FeZr@NC, (f) VCu@NC, (g) FeCu@NC, (h) PtMo@NC, and (i) CoCu@NC. IS, TS, and FS denote the initial state, transition state, and final state, respectively.


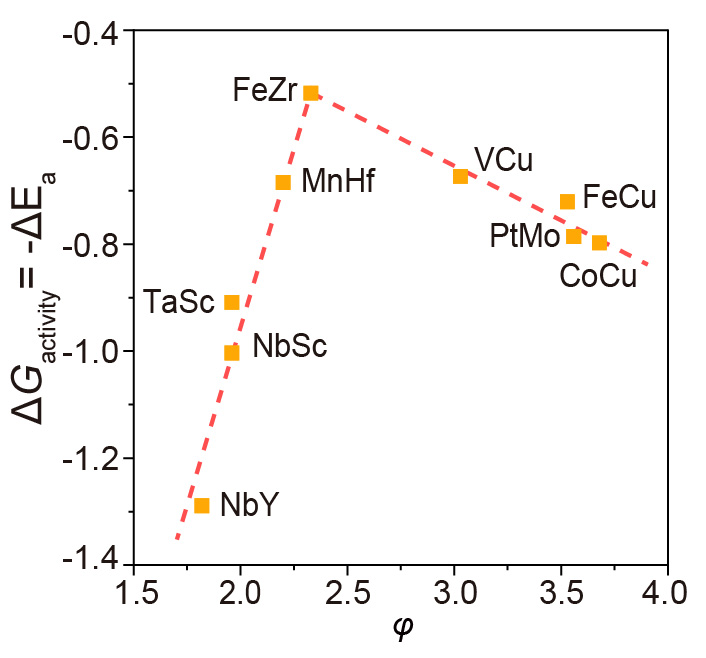


## **Figure S16.** Relationship between Δ*G*_activity_ = - ΔE_a_ and *φ*.


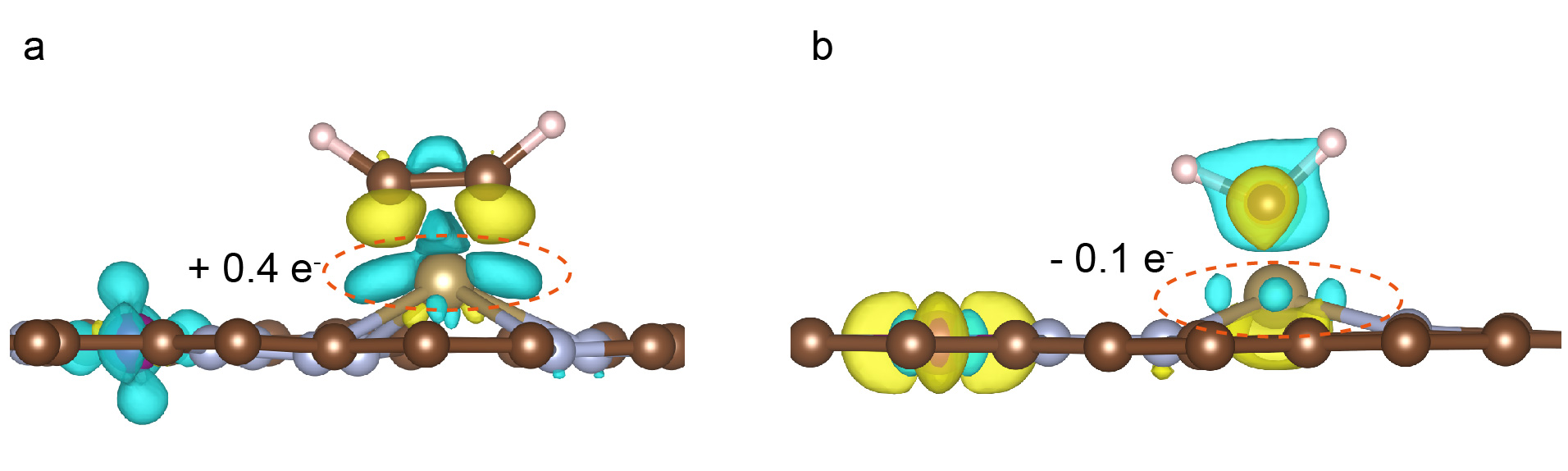


## **Fig**ure **S17.** The Charge difference for (a) C–C coupling and (b) hydrogenation. Charge accumulation is in blue and depletion is in yellow. The specific amount of charge transfer shown in the figure is known from the Bader charge analysis. FeZr@NC is a representative. Purple, Fe; dark yellow, Zr; silver, N; brown, C; white, H.


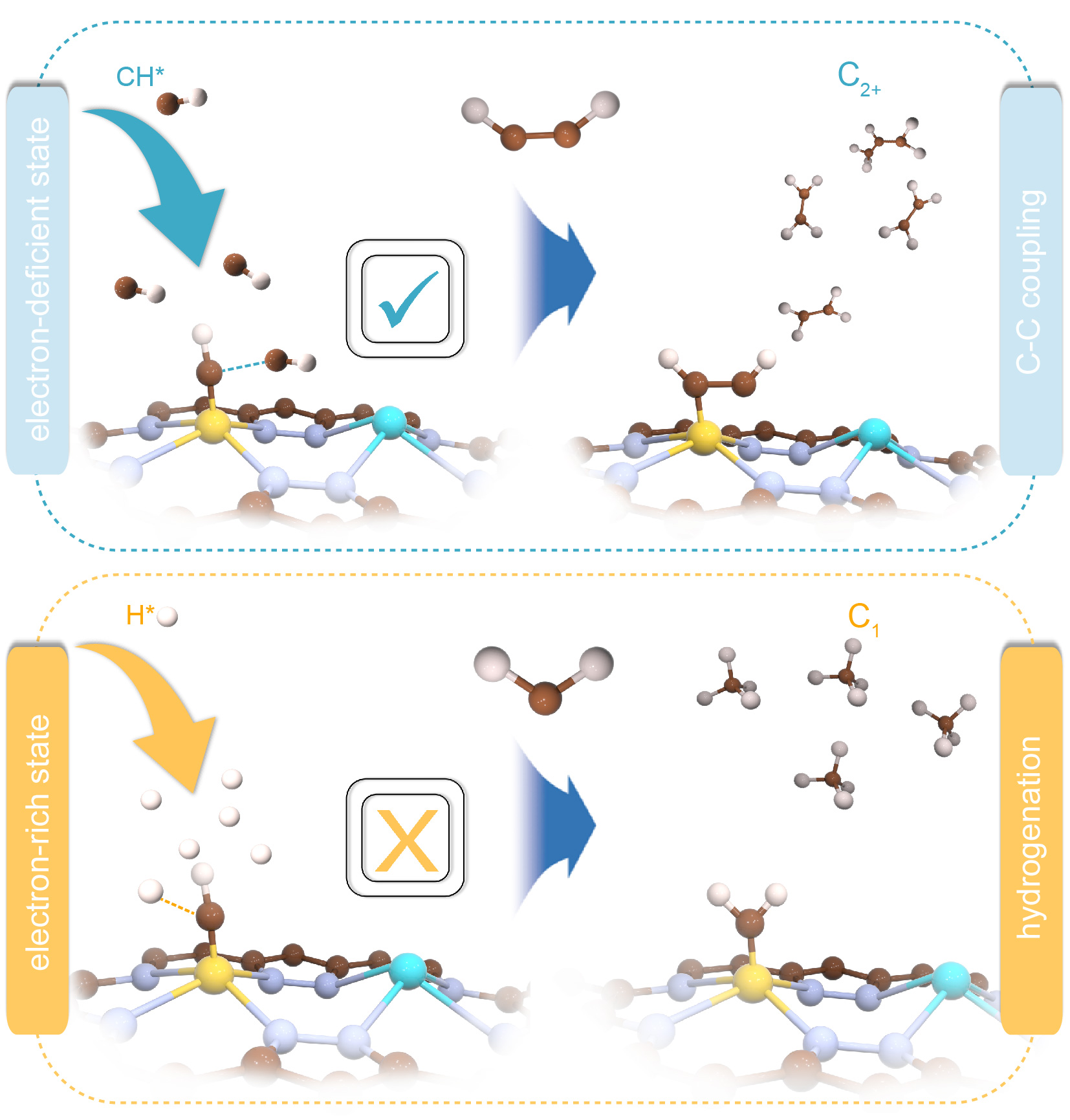


## **Fig**ure **S18.** Schematic diagram illustrating the selection of C-C coupling or hydrogenation.


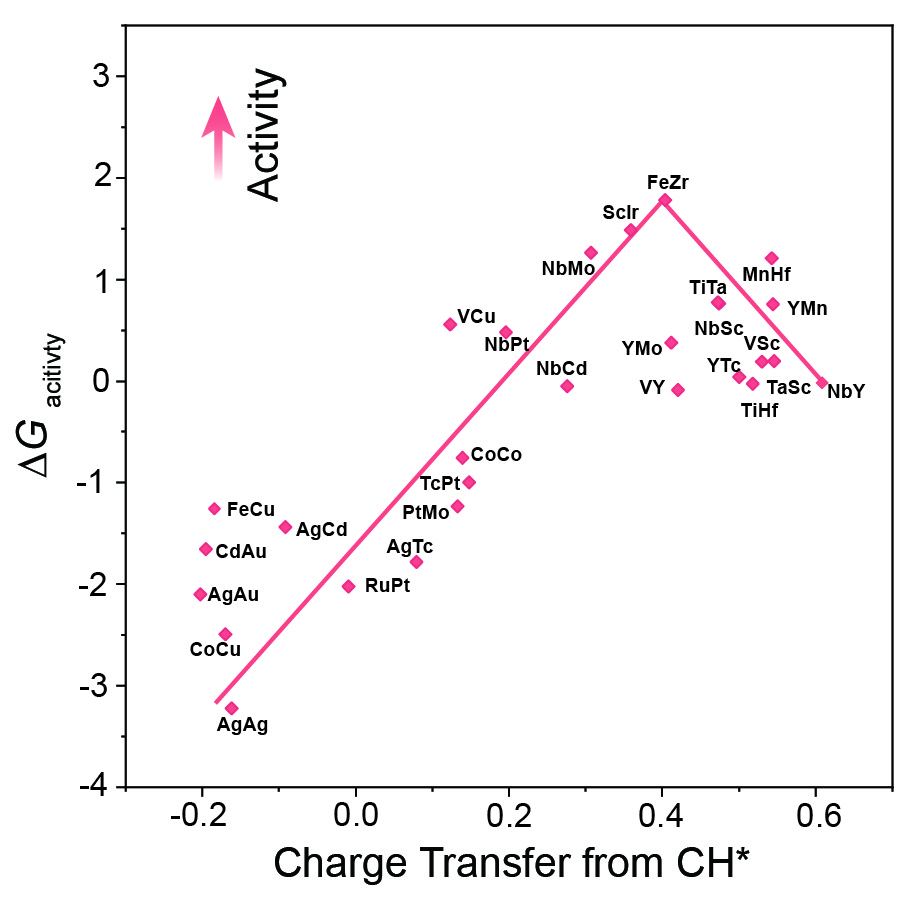


## **Fig**ure **S19.** Charge transfer from CH^*^ versus Δ*G*_activity_.


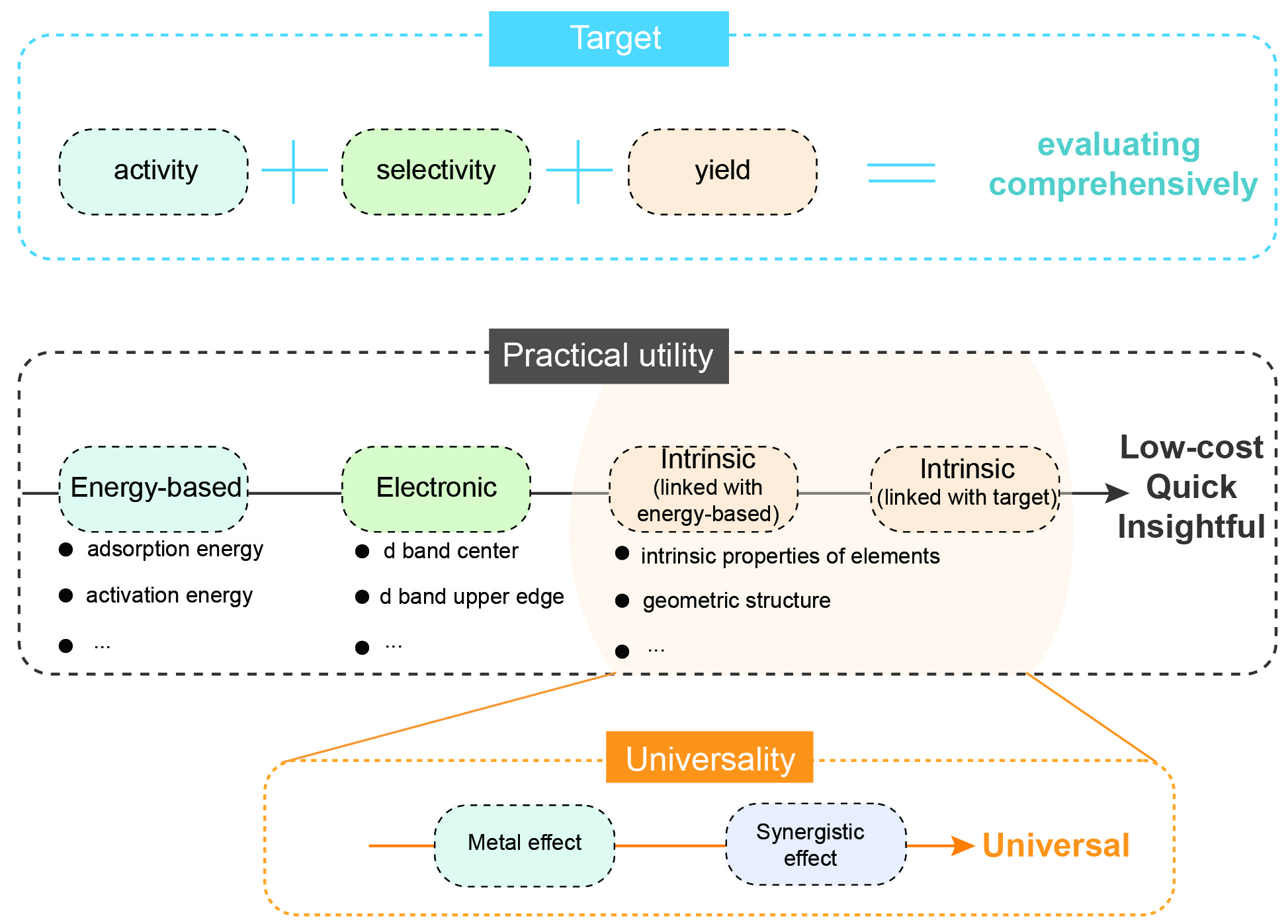


## Figure S20. Schematic diagram of the classification and evaluation for descriptors.


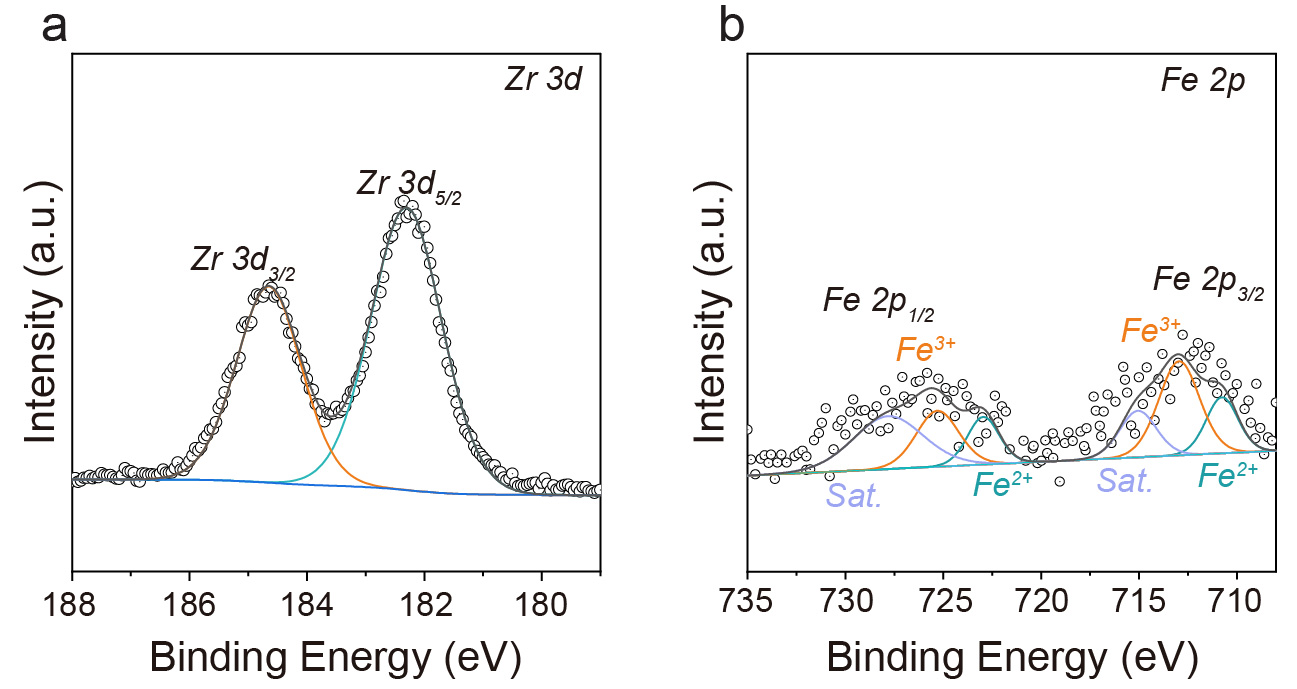


## **Fig**ure **S21.** (a) Zr 3d and (b) Fe 2p XPS spectra of FeZr@NC.


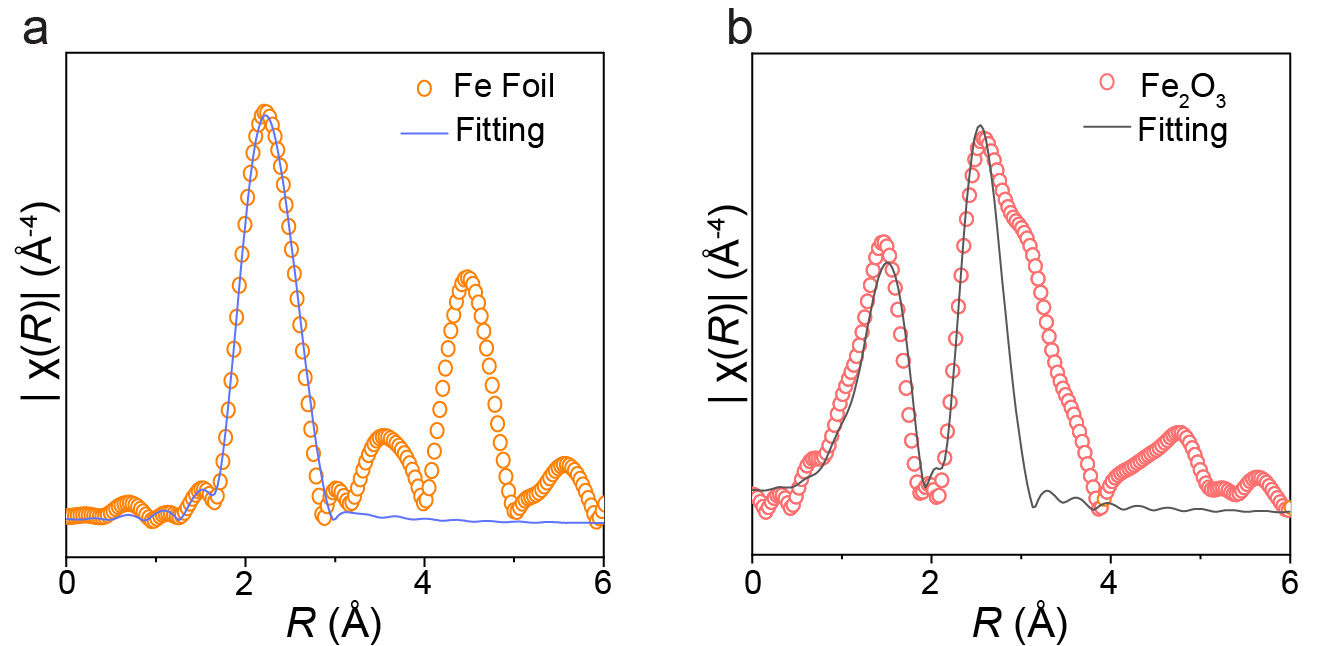


## **Fig**ure **S22.** The corresponding FT-EXAFS fitting curves of (a) Fe foil and (b) Fe_2_O_3_.


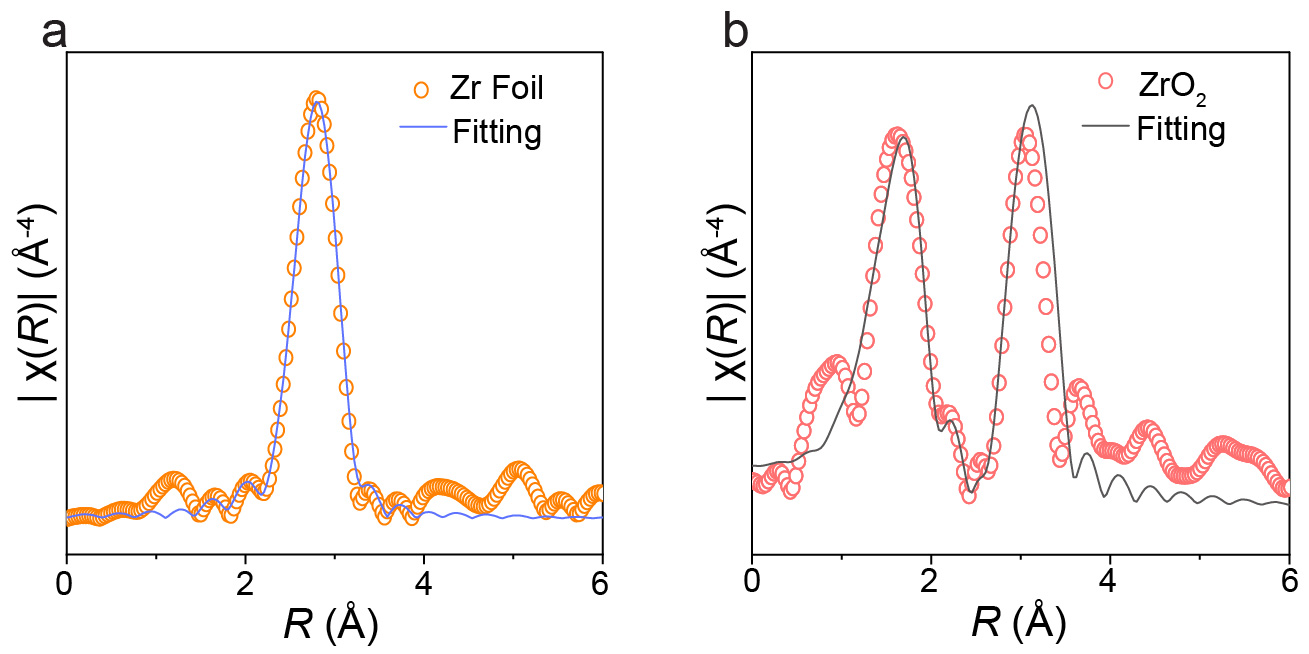


## **Fig**ure **S23.** The corresponding FT-EXAFS fitting curves of (a) Zr foil and (b) ZrO_2_.


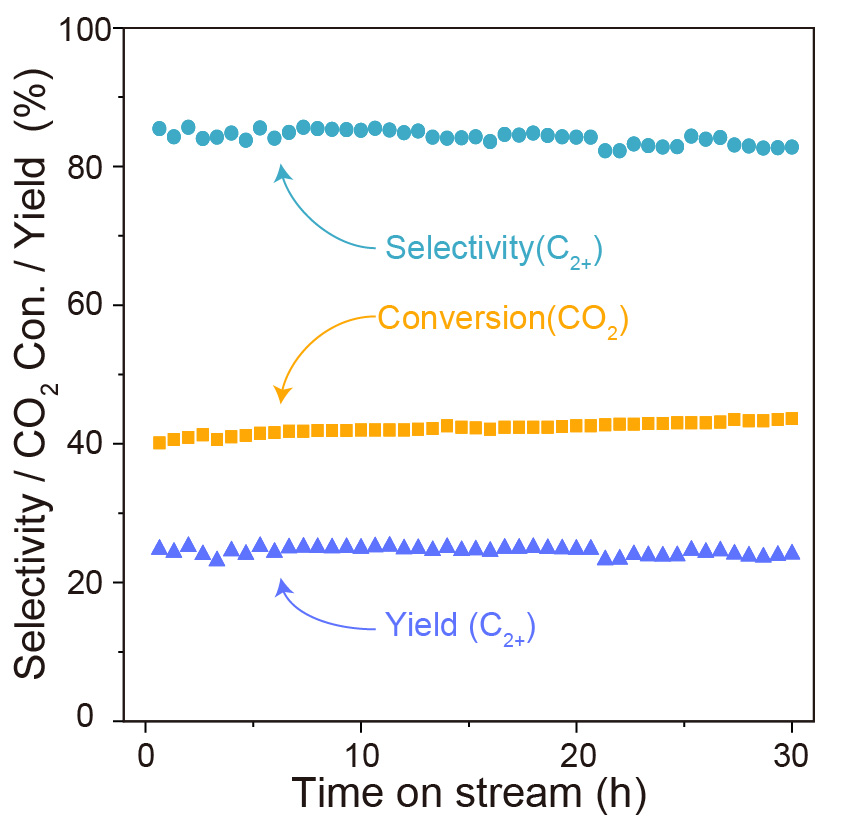


## **Fig**ure **S24.** Stability test for FeZr@NC catalyst. Reaction conditions: 370 ^o^C, 3 MPa, 12000 mL/g_cat_/h.


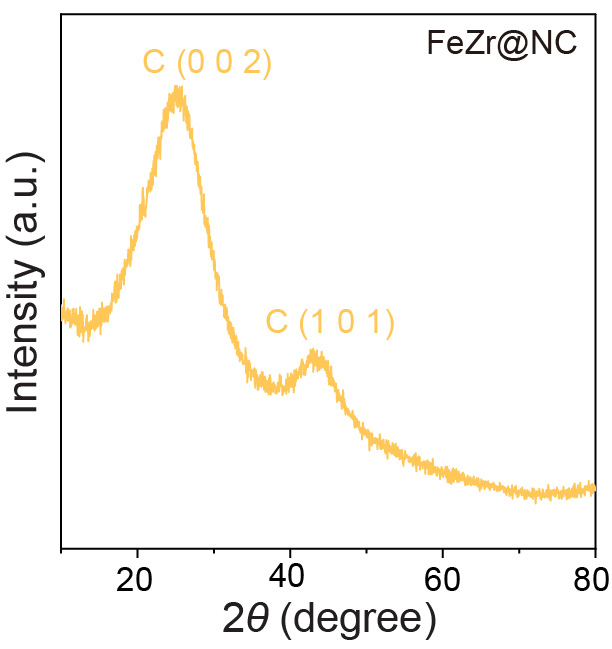


## **Fig**ure **S25.** XRD patterns of the spent FeZr@NC.


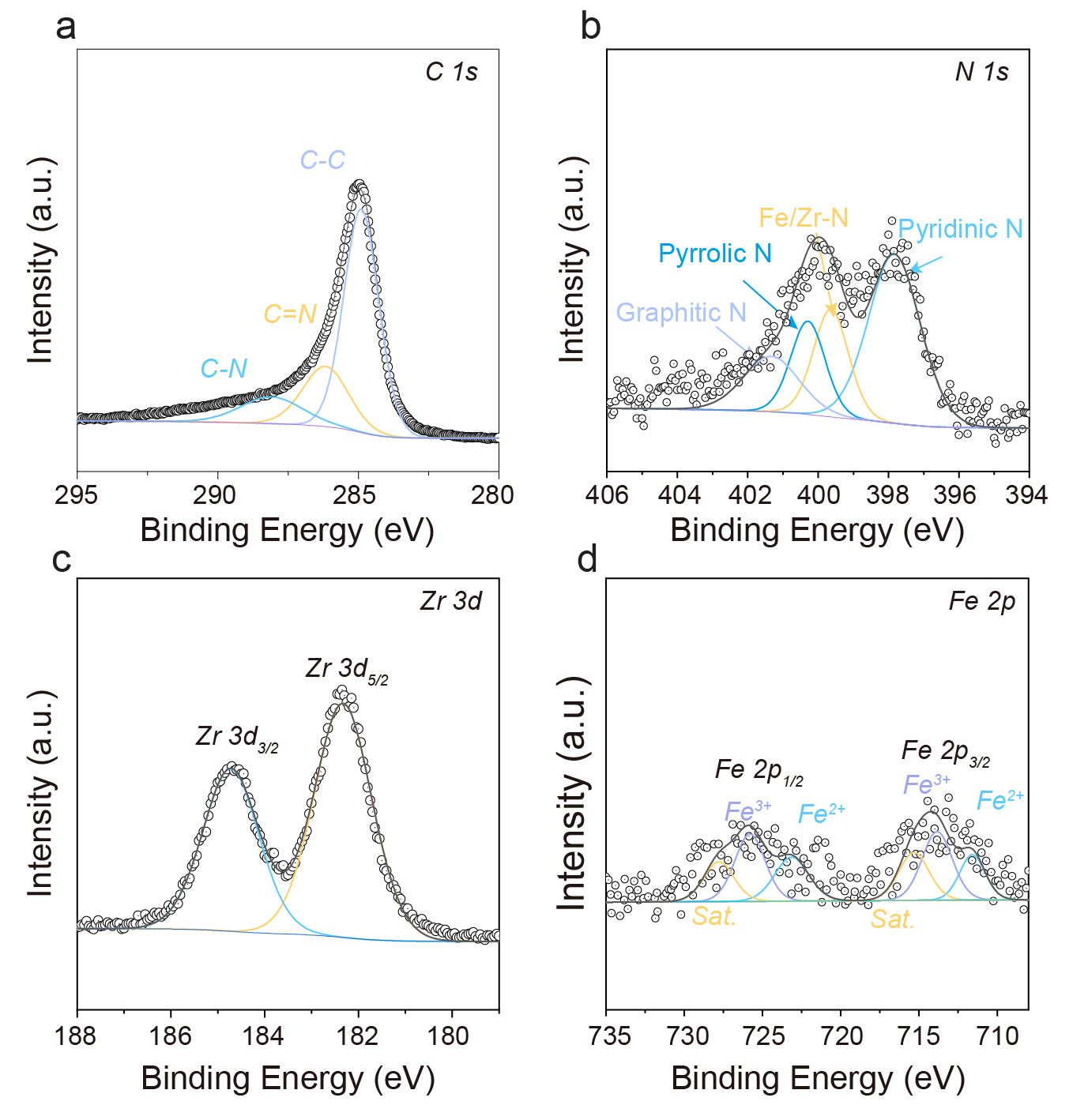


## **Figure S26.** (a) C 1s, (b) N 1s, (c) Zr 3d, and (d) Fe 2p XPS spectra of the spent FeZr@NC.


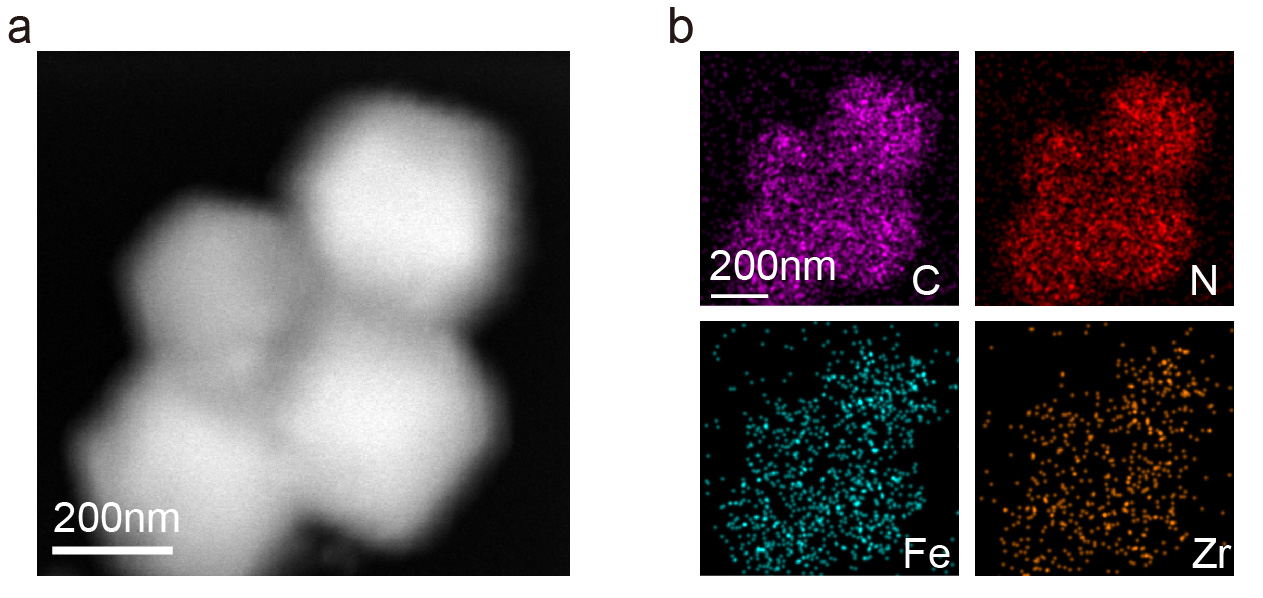


## **Figure S27.** (a)TEM images and (b) EDS mapping of the spent FeZr@NC.


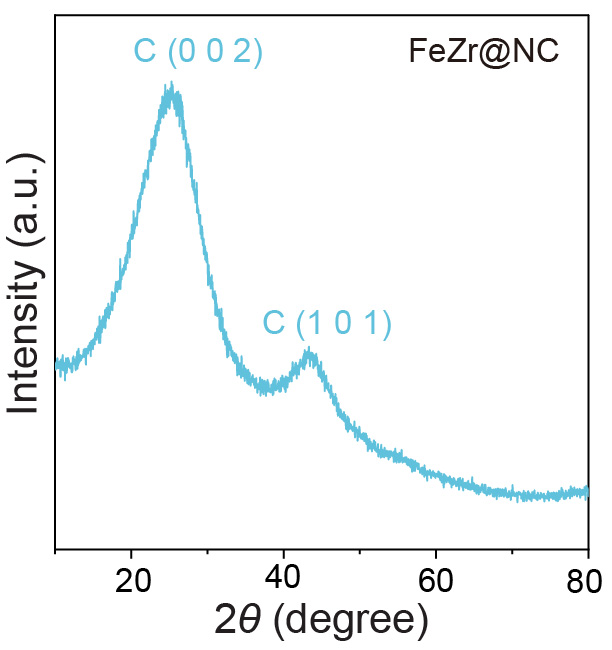


## **Fig**ure **S28.** XRD patterns of FeZr@NC of reproducibility experiments.


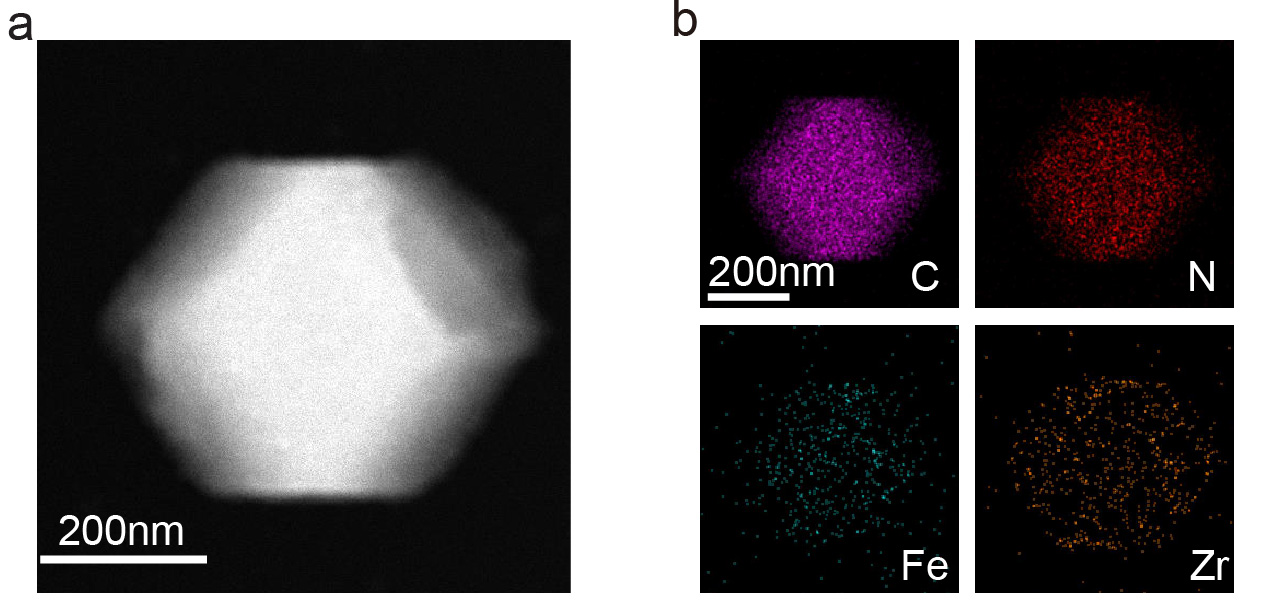


## **Figure S29.** (a) TEM images and (b) EDS mapping for FeZr@NC of reproducibility experiments.


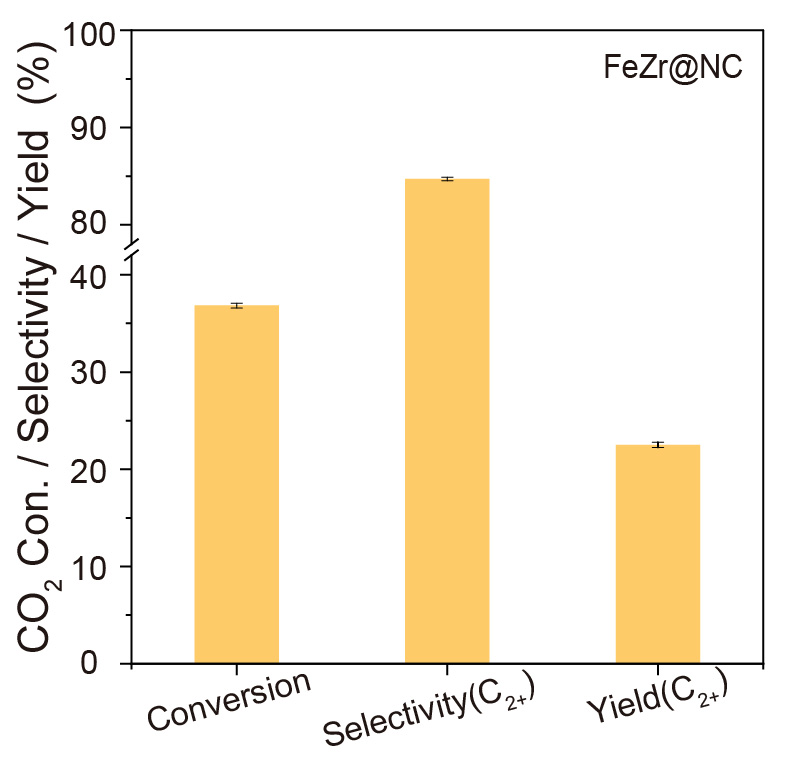


Figure S30. CO_2_ conversion, C_2+_ selectivity, and C_2+_ yield of FeZr@NC. Reaction conditions: 360 ^o^C, 3 MPa, 12000 mL/g_cat_/h. Error bars correspond to the standard deviation of three independent measurements.

## **Table S1.** The 12 features used in the ML model

| **Features** |  |
| --- | --- |
| Atomic mass | *M* |
| Atomic radius | *rM* |
| d electron number | *θ_d_* |
| s electron number | *θ_s_* |
| Outermost electron number | *N_e_* |
| Atomic number in the periodic table of elements | *N_atom_* |
| Electronegativity | *χ* |
| Electron affinity | *EA* |
| First ionization energy | *I* |
| Mendelev number | *N_men_* |
| Group number | *N_group_* |
| Period number | *P* |

## **Table S2.** Atomic features obtained from the literature.^[16-19]^

| **Metal** | ***M*** | ***rM*** | ***θ_d_*** | ***θ_s_*** | ***N_e_*** | ***N_atom_*** | ***χ*** | ***EA*** | ***I*** | ***N_men_*** | ***N_group_*** | ***P*** |
| --- | --- | --- | --- | --- | --- | --- | --- | --- | --- | --- | --- | --- |
| Sc | 44.96 | 211 | 2 | 2 | 3 | 21 | 1.36 | -0.73 | 633.1 | 11 | 3 | 3 |
| Ti | 47.88 | 187 | 3 | 2 | 4 | 22 | 1.54 | -0.02 | 658.8 | 43 | 4 | 3 |
| V | 50.94 | 179 | 4 | 2 | 5 | 23 | 1.63 | 0.63 | 650.9 | 46 | 5 | 3 |
| Cr | 52 | 189 | 5 | 1 | 6 | 24 | 1.66 | 0.97 | 652.9 | 49 | 6 | 3 |
| Mn | 54.94 | 197 | 6 | 2 | 7 | 25 | 1.55 | 0.97 | 717.3 | 52 | 7 | 3 |
| Fe | 55.85 | 194 | 7 | 2 | 8 | 26 | 1.83 | 0.46 | 762.5 | 55 | 8 | 3 |
| Co | 58.93 | 192 | 8 | 2 | 9 | 27 | 1.88 | 1.06 | 760.4 | 58 | 9 | 3 |
| Ni | 58.69 | 163 | 9 | 2 | 10 | 28 | 1.91 | 1.62 | 737.1 | 61 | 10 | 3 |
| Cu | 63.55 | 140 | 10 | 1 | 11 | 29 | 1.90 | 1.80 | 745.5 | 64 | 11 | 3 |
| Zn | 65.39 | 139 | 10 | 2 | 12 | 30 | 1.65 | 0.09 | 906.4 | 69 | 12 | 3 |
| Y | 88.91 | 219 | 2 | 2 | 3 | 39 | 1.22 | -0.40 | 600.0 | 12 | 3 | 4 |
| Zr | 91.22 | 186 | 3 | 2 | 4 | 40 | 1.33 | 0.45 | 640.1 | 44 | 4 | 4 |
| Nb | 92.91 | 207 | 4 | 1 | 5 | 41 | 1.60 | 1.13 | 652.1 | 47 | 5 | 4 |
| Mo | 95.96 | 209 | 5 | 1 | 6 | 42 | 2.16 | 1.18 | 684.3 | 50 | 6 | 4 |
| Tc | 98 | 209 | 6 | 2 | 7 | 43 | 1.90 | 0.99 | 702.0 | 53 | 7 | 4 |
| Ru | 101.1 | 207 | 7 | 1 | 8 | 44 | 2.20 | 1.51 | 710.2 | 56 | 8 | 4 |
| Rh | 102.9 | 195 | 8 | 1 | 9 | 45 | 2.28 | 1.68 | 719.7 | 59 | 9 | 4 |
| Pd | 106.4 | 202 | 9 | 0 | 10 | 46 | 2.2 | 1.02 | 804.4 | 62 | 10 | 4 |
| Ag | 107.9 | 172 | 10 | 1 | 11 | 47 | 1.93 | 2.00 | 731.0 | 65 | 11 | 4 |
| Cd | 112.4 | 158 | 10 | 2 | 12 | 48 | 1.69 | 0.27 | 867.8 | 70 | 12 | 4 |
| Hf | 178.5 | 212 | 3 | 2 | 4 | 72 | 1.30 | 0.63 | 658.5 | 45 | 4 | 5 |
| Ta | 180.9 | 217 | 4 | 2 | 5 | 73 | 1.50 | 0.15 | 761.0 | 48 | 5 | 5 |
| W | 183.9 | 210 | 5 | 2 | 6 | 74 | 2.36 | 1.23 | 770.0 | 51 | 6 | 5 |
| Re | 186.2 | 217 | 6 | 2 | 7 | 75 | 1.90 | 0.38 | 760.0 | 54 | 7 | 5 |
| Os | 190.2 | 216 | 7 | 2 | 8 | 76 | 2.20 | 1.44 | 840.0 | 57 | 8 | 5 |
| Ir | 192.2 | 202 | 8 | 2 | 9 | 77 | 2.20 | 1.97 | 880.0 | 60 | 9 | 5 |
| Pt | 195.1 | 209 | 9 | 1 | 10 | 78 | 2.28 | 2.56 | 870.0 | 63 | 10 | 5 |
| Au | 197 | 166 | 10 | 1 | 11 | 79 | 2.54 | 2.80 | 890.1 | 66 | 11 | 5 |

## **Table S3.** Features of different input feature sets. The subscript 1 represents the TM_1_ atom of DACs, while those with subscript 2 represent the TM_2_ atom of DACs

| **Input Feature Sets** | **Features** |
| --- | --- |
| Feature 1 | *M*.1, *rM*.1, *θ_d_*.1, *θ_s_*.1, *N_e_*.1, *N_atom_*.1, *χ*.1, *EA*.1, *I*.1, *N_men_*.1, *N_gruop_*.1, *P*.1, *M*.2, *rM*.2, *θ_d_*.2, *θ_s_*.2, *N_e_*.2, *N_atom_*.2, *χ*.2, *EA*.1, *I*.1, *N_men_*.2, *N_gruop_*.2, *P*.2 |
| Feature 2 | *rM*.1, *θ_d_.1*, *θ_s_.1*, *χ.1*, *P.1*, *rM.2*, *θ_d_.2*, *θ_s_*.2, *χ*.2, *P.2* |
| Feature 3 | *rM.1*, *θ_d_.1*, *θ_s_.1*, *χ.1*, *P.1*, *rM.2*, *θ_d_.2*, *θ_s_.2*, *χ.2*, *P.2*, *rM.1*+*rM.2*, *rM.1*-*rM.2*, *θ_d.1_*+*θ_d.2_*, *θ_d.1_*-*θ_d.2_*, *θ_.1s_*+*θ_s.2_*,  *θ_s.1_*-*θ_s.2_*, *χ_.1_*+*χ.2*, *χ_.1_*-*χ.2*, *P_.1_*+*P.2*, *P_.1_*-*P.2*, max*rM*, min*rM*, max*θ_d_*, min*θ_d_*, max*θ_s_*, min*θ_s_*, max*χ*, min*χ*, max*P*, min*P*, *rM_.1_***rM.2*, *rM_.1_*/*rM.2*, *θ_d.1_***θ_d.2_*, *θ_d.1_*/*θ_d.2_*, *θ_s.1_***θ_s.2_*, *χ_.1_***χ.2*, *χ_.1_*/*χ.2*, *P_.1_***P.2*, *P.1*/*P.2* |

## **Table S4.** The specific values of ΔΔ*G*_selectivity_

| **M_1_M_2_** | **ΔΔ*G*_selectivity_** |
| --- | --- |
| ScTi | -0.20 |
| ScCu | 1.25 |
| TiV | 0.61 |
| TiCo | 1.14 |
| TiCd | 0.84 |
| TiHf | -1.10 |
| VCo | 1.66 |
| VZn | 1.71 |
| VMo | 0.53 |
| VPt | 0.72 |
| CrCo | 1.76 |
| CrY | 0.08 |
| CrRu | 1.06 |
| CrAg | 0.65 |
| CrHf | -0.36 |
| MnFe | 0.62 |
| MnZn | 0.851 |
| MnRh | 0.90 |
| FeZn | -1.11 |
| FeAg | 1.43 |
| FeAu | 0.90 |
| CoZr | 0.25 |
| CoW | -0.22 |
| CoIr | 1.35 |
| NiZn | 0.92 |
| NiZr | -0.06 |
| NiCd | 1.28 |
| NiTa | -0.16 |
| NiOs | 1.25 |
| CuZn | 1.24 |
| CuZr | 1.05 |
| CuCd | 1.34 |
| CuW | 1.18 |
| ZnOs | 1.13 |
| YNb | -1.20 |
| YMo | -1.03 |
| ZrNb | 0.69 |
| NbMo | 1.94 |
| MoW | -0.45 |
| MoPt | 1.96 |
| TcRu | 0.96 |
| TcAg | 1.97 |
| TcHf | -0.59 |
| TcAu | 1.65 |
| RuPd | 1.15 |
| RuW | 0.35 |
| RuPt | 2.49 |
| RhPd | 1.30 |
| RhCd | 1.25 |
| RhTa | -0.33 |
| RhIr | 1.26 |
| PdAg | 1.61 |
| PdTa | 0.81 |
| PdIr | 0.57 |
| AgCd | 2.82 |
| HfNb | 0.59 |
| HfY | 0.89 |
| TaOs | -0.05 |
| OsIr | 0.97 |
| IrAu | 1.19 |
| PtAu | 0.64 |
| TiTi | -0.04 |
| CoCo | 1.90 |
| MoMo | 0.31 |
| CdCd | 1.14 |
| OsOs | 0.69 |
| AuAu | 1.12 |

## Table S5. The value of Δ*G*_activity_ (Δ*G*_activity_ = -Δ*G*_coupling_).

| **M_1_M_2_** | **Δ*G*_activity_** |
| --- | --- |
| NbY | -0.006 |
| VY | -0.089 |
| TiHf | 0.042 |
| YMo | 0.376 |
| TaSc | 0.189 |
| NbSc | -0.027 |
| VSc | 0.196 |
| Ymn | 0.758 |
| YTc | 0.765 |
| MnHf | 1.211 |
| TiTa | 0.772 |
| FeZr | 1.780 |
| ScIr | 1.484 |
| NbMo | 1.263 |
| NbPt | 0.479 |
| NbCd | -0.048 |
| Vcu | 0.555 |
| TcPt | -0.995 |
| CoCo | -0.758 |
| AgTc | -1.782 |
| FeCu | -1.257 |
| PtMo | -1.235 |
| CoCu | -2.495 |
| CdAu | -1.655 |
| AgCd | -1.436 |
| RuPt | -2.020 |
| AgAu | -2.099 |
| AgAg | -3.220 |

## **Table S6.** GBR Model performance evaluation with 20 distinct partitioning configurations of R^2^, and RMSE score.

| **Random state number** | **R^2^** | **RMSE** |
| --- | --- | --- |
| 0 | 0.98825 | 0.08425 |
| 1 | 0.99254 | 0.06586 |
| 2 | 0.99090 | 0.07455 |
| 3 | 0.98950 | 0.07912 |
| 4 | 0.98933 | 0.08109 |
| 5 | 0.99054 | 0.07761 |
| 6 | 0.99049 | 0.07902 |
| 7 | 0.98982 | 0.08055 |
| 8 | 0.99137 | 0.07234 |
| 9 | 0.98968 | 0.08303 |
| 10 | 0.99072 | 0.07811 |
| 11 | 0.99058 | 0.07953 |
| 12 | 0.98894 | 0.08084 |
| 13 | 0.98931 | 0.07989 |
| 14 | 0.98669 | 0.08639 |
| 15 | 0.99008 | 0.08040 |
| 16 | 0.99081 | 0.07300 |
| 17 | 0.98842 | 0.08286 |
| 18 | 0.98798 | 0.08188 |
| 19 | 0.98685 | 0.08352 |
| 20 | 0.99289 | 0.06430 |

## Table S7. Bader charge for the coupling and hydrogenation process.

| **Reaction Pathway** | **Absorption Intermediate** | **Electron Transferred Numbers** |
| --- | --- | --- |
| Coupling | CH^*^ | + 0.4 e^-^ |
| Hydrogenation | H^*^ | - 0.1 e^-^ |

## Table S8. The values for the difference between transferring electrons to CH^*^ and acquiring electrons from H^*^ of DACs, electron transfer to CH^*^, and the d-band center of active metal sites

| **M_1_M_2_** | **Difference of Charge Transfer** | **Electron Transfer to CH^*^** | **d-band center** |
| --- | --- | --- | --- |
| NbY | 0.556 | 0.608 | 0.239 |
| VY | 0.352 | 0.420 | 0.452 |
| TiHf | 0.478 | 0.500 | 0.562 |
| YMo | 0.266 | 0.412 | -0.194 |
| ScTa | 0.472 | 0.518 | 0.355 |
| NbSc | 0.487 | 0.546 | 0.391 |
| VSc | 0.487 | 0.530 | 0.594 |
| YMn | 0.461 | 0.474 | 0.302 |
| YTc | 0.449 | 0.545 | -0.128 |
| MnHf | 0.388 | 0.472 | 0.317 |
| TiTa | 0.470 | 0.543 | -0.212 |
| FeZr | 0.305 | 0.404 | -0.287 |
| ScIr | 0.262 | 0.359 | -0.649 |
| NbMo | 0.248 | 0.307 | -0.483 |
| NbPt | 0.103 | 0.196 | -1.931 |
| NbCd | 0.231 | 0.276 | -2.375 |
| Vcu | 0.027 | 0.123 | -1.551 |
| TcPt | 0.029 | 0.148 | -2.297 |
| CoCo | 0.054 | 0.139 | -1.678 |
| AgTc | 0.002 | 0.079 | -2.972 |
| FeCu | -0.251 | -0.184 | -1.848 |
| PtMo | 0.034 | 0.133 | -2.074 |
| CoCu | -0.289 | -0.170 | -1.990 |
| CdAu | -0.286 | -0.195 | -3.167 |
| AgCd | -0.137 | -0.091 | -3.756 |
| RuPt | -0.095 | -0.010 | -2.268 |
| AgAu | -0.345 | -0.202 | -4.736 |
| AgAg | -0.283 | -0.161 | -4.514 |

## Table S9. The value of -Δ*G*_coupling_ and ΔΔ*G*_selectivity_ for CO^*^–CO^*^, CHO^*^–CO^*^, and CH^*^–CH_2_^*^ processes

| **M_1_M_2_** | **-Δ*G*_coupling CO*_**_–_**_CO*_** | **ΔΔ*G*_selectivity CO*_**_–_**_CO*_** | **-Δ*G*_coupling CHO*_**_–_**_CO*_** | **ΔΔ*G*_selectivity CHO*_**_–_**_CO*_** | **-Δ*G*_coupling CH*_**_–_**_CH2*_** | **ΔΔ*G*_selectivity CH*_**_–_**_CH2*_** |
| --- | --- | --- | --- | --- | --- | --- |
| NbY | -0.291 | 0.413 | 0.352 | 0.816 | 0.043 | 0.516 |
| TiHf | -0.298 | 0.557 | 0.294 | 0.680 | 0.488 | 0.916 |
| NbSc | 0.086 | 1.222 | 0.482 | 0.596 | 0.282 | 0.771 |
| YTc | 0.028 | 0.165 | 0.561 | 0.262 | 0.838 | 0.346 |
| TiTa | 0.373 | 0.216 | 0.591 | 0.398 | 1.018 | 0.142 |
| TaMn | 0.347 | -0.508 | 0.667 | -0.906 | 1.007 | -0.128 |
| NbPt | 0.086 | -0.331 | -0.053 | -0.179 | 0.258 | -0.625 |
| TcPt | -0.270 | -0.634 | -0.456 | -0.435 | -0.390 | -0.385 |
| PtMo | -0.550 | -0.147 | -0.582 | -0.632 | -0.567 | -1.262 |
| AgAu | -0.579 | -0.442 | -1.241 | -0.815 | -0.665 | -0.648 |

## Table S10. Detailed hyperparameters for each ML model

|  | **Hyperparameters** |
| --- | --- |
| **DTR** | max_depth: 7, max_features: "auto",  min_samples_leaf: 2, min_samples_split:2, |
| **ETR** | criterion: 'mse', splitter: 'random', max_depth: 15,  min_samples_split: 4, min_samples_leaf: 1,  min_weight_fraction_leaf:0.0 |
| **GBR** | learning_rate: 0.01, n_estimators:2000, max_depth: 3,  max_features: None, min_samples_leaf: 2,  min_samples_split:25, subsample: 0.9, |
| **KNR** | n_neighbors: 4 |
| **KRR** | alpha: 1.0, kernel: "linear" |
| **LR** | Max_iter: 100, penalty: “l2”, solver = “lbfgs” |
| **RF** | max_depth: 7, n_estimators: 3000, max_features: 'auto',  min_samples_split: 2, min_samples_leaf:2 |
| **XGBR** | learning_rate: 0.01, n_estimators :1500, max_depth:7, min_child_weight :5, gamma: 0.01, subsample: 1, colsample_bytree: 0.9, reg_alpha: 0, reg_lambda: 0 |
| **GBM** | learning_rate: 0.05, n_estimators: 500, max_depth: 5, num_leaves: 10, subsample: 0.9, colsample_bytree: 0.9, reg_alpha: 0.1, reg_lambda: 0.1, min_child_sample: 20 |
| **SVR** | kernel: 'rbf', C: 5, epsilon: 0.1, degree: 3, cache_size: 2000 |

## **Table S11.** The difference between the descriptors in previous papers and this work. ‘−’ represents that this item cannot be evaluated.

| **Paper** | **Target** | | | **Practical utility** | | **Universality** | |
| --- | --- | --- | --- | --- | --- | --- | --- |
|  | activity | selectivity | yield | Intrinsic  (linked with the target) | | metal effect | synergistic effect |
| This work | √ | √ | √ | | √ | √ | √ |
| A^[15]^ | × | √ | × | | √ | √ | √ |
| B^[14]^ | × | √ | × | | √ | √ | √ |
| C^[20]^ | √ | × | × | | × | - | - |
| D^[21]^ | × | √ | × | | × | - | - |
| E^[22]^ | √ | × | × | | √ | √ | × |
| F^[23]^ | √ | × | × | | √ | √ | √ |
| G^[24]^ | √ | × | × | | × | √ | × |

## Table S12. Chemical composition of the FeZr@CN recorded by XPS

| **Elements** | **Assignment** | **Position (eV)** | **Content (at. %)** |
| --- | --- | --- | --- |
| C | C–C | 284.8 | 74.4 |
|  | C=N | 286.1 | 16.9 |
|  | C–N | 288.0 | 8.7 |
| N | Pyridinic N | 398.2 | 40.1 |
|  | Fe/Zr–N | 399.4 | 19.7 |
|  | Pyrrolic N | 400.2 | 22.8 |
|  | Graphitic N | 401.3 | 17.4 |
| Zr | 3d_5/2_ | 182.4 | 61.6 |
|  | 3d_3/2_ | 184.7 | 38.4 |

## Table S13. EXAFS fitting results for different materials at Fe K-edge

| **Materials** | **Model** | **Shell** | ***CN*** | **R (Å)** | **σ^2^(Å^2^)** | **ΔE_o_(eV)** | **R factor** |
| --- | --- | --- | --- | --- | --- | --- | --- |
| Fe foil |  | Fe–Fe_1_ | 8 | 2.46±0.008 | 0.006 | 4.56±1.18 | 0.002 |
|  |  | Fe–Fe_2_ | 6 | 2.84±0.023 | 0.005 |  |  |
| FeZr@NC | Model 1 | Fe–N | 4.5±0.3 | 1.96±0.005 | 0.011 | -4.57±2.21 | 0.010 |
|  |  | Fe–Zr | 1.0±0.6 | 2.23±0.049 | 0.022 |  |  |
|  | Model 2 | Fe–N | 3.0 | 2.40±0.085 | 0.039 | 56.46±0.13 | 0.216 |
|  |  | Fe–Zr | 1.0 | 1.29±0.030 | 0.026 |  |  |
|  | Model 3 | Fe–N | 2.0 | 2.47±0.115 | 0.018 | 52.15±0.08 | 0.448 |
|  |  | Fe–Zr | 1.0 | 2.06±0.051 | 0.012 |  |  |
|  | Model 4 | Fe-N | 4.0 | 2.17±0.058 | 0.002 | -66.42±13.32 | 0.180 |

CN - Coordination number

R - Interatomic distance

σ^2^ - Debye-Waller factor (thermal and static disorder in absorber-scatterer distances)

ΔE_0_ - Edge energy shift (the difference between the zero kinetic energy value of the sample and that of the standard theoretical model)

R factor - Goodness of fitting

The accuracy of parameters: σ^2^, ±20%.

S_0_^2^ was fixed at 0.80, obtained from the Fe foil measured at the same time, respectively.

## Table S14. EXAFS fitting results for different materials at Zr K-edge

| **Materials** | **Model** | **Shell** | **CN** | **R (Å)** | **σ^2^(Å^2^)** | **ΔE_o_(eV)** | **R factor** |
| --- | --- | --- | --- | --- | --- | --- | --- |
| Zr foil |  | Zr–Zr_1_ | 6 | 3.14±0.047 | 0.003 | -8.83±0.47 | 0.002 |
|  |  | Zr–Zr_2_ | 6 | 3.26±0.025 | 0.002 |  |  |
| FeZr@NC | Model 1 | Zr–N | 4.2±0.4 | 2.10±0.047 | 0.001 | -5.00±3.08 | 0.016 |
|  |  | Zr–Fe | 1.2±0.3 | 2.32±0.016 | 0.007 |  |  |
|  | Model 2 | Zr–N | 3.0 | 1.88±0.029 | 0.027 | 33.07±2.37 | 0.151 |
|  |  | Zr–Fe | 1.0 | 2.42±0.165 | 0.009 |  |  |
|  | Model 3 | Zr–N | 2.0 | 1.81±0.122 | 0.007 | 98.10±7.08 | 0.638 |
|  |  | Zr–Fe | 1.0 | 2.84±0.110 | 0.042 |  |  |
|  | Model 4 | Zr-N | 4.0 | 2.60±0.087 | 0.010 | 75.58±20.41 | 0.608 |

CN - Coordination number

R - Interatomic distance

σ^2^ - Debye-Waller factor (thermal and static disorder in absorber-scatterer distances)

ΔE_0_ - Edge energy shift (the difference between the zero kinetic energy value of the sample and that of the standard theoretical model)

R factor - Goodness of fitting

The accuracy of parameters: σ^2^, ±20%.

S_0_^2^ was fixed at 0.76, obtained from the Zr foil measured at the same time, respectively.

## Table S15. Comparison studies on the catalytic performance of various catalysts for CO_2_ into light olefins

| **GHSV**  **(mL/g_cat_/h)** | **Tem**  **(^o^C)** | **P**  **(MPa)** | **X(CO_2_)**  **(%)** | **S(C_2+_)**  **(%)** | **S(CO)**  **%** | **Y(C_2+_)**  **%** | **catalyst** | **Ref** |
| --- | --- | --- | --- | --- | --- | --- | --- | --- |
| **12000** | **360** | **3** | **36.8** | **84.8** | **13.2** | **22.6** | **FeZr/CN** | **This work** |
| 8000 | 380 | 3 | 13.8 | 98.8 | 40.0 | 8.2 | ZnZrOx&bio-SAPO-34 | ^[25]^ |
| 8000 | 380 | 3 | 9.4 | 97.1 | 43.0 | 5.2 | ZnZrOx&dia-SAPO-34 |  |
| 3600 | 370 | 3 | 15.0 | 97.7 | 49.0 | 7.5 | ZnAl_2_O_4_&SAPO-34 | ^[26]^ |
| 5400 | 370 | 3 | 13.0 | 97.7 | 46.0 | 6.9 | ZnGa_2_O_4_&SAPO-34 | ^[27]^ |
| 3600 | 380 | 2 | 12.6 | 97.0 | 47.0 | 6.5 | ZnO-ZrO_2_&SAPO-34 | ^[28]^ |
| 5750 | 400 | 3 | 29.0 | 93.1 | 78.2 | 5.9 | In–Zr/SAPO-34 |  |
| 2000 | 320 | 2 | 9.1 | 19.2 | 57.4 | 0.7 | ZnAlOx | ^[29]^ |
| 1200 | 320 | 4 | 14.1 | 19.4 | 43.7 | 1.5 | ZnZrO/HZSM-5 | ^[30]^ |
| 4000 | 320 | 3 | 36.2 | 11.0 | 16.4 | 2.9 | Na–Fe_3_O_4_/HZSM-5 | ^[31]^ |
| / | 350 | 3.5 | 22.1 | 72.2 | 34.5 | / | Zn_0.5_Ce_0.2_Zr_1.8_O_4_/H-RUB-13(200) | ^[32]^ |
| 4500 | 360 | 2 | 9.1 | 93.0 | 32.0 | 5.8 | ZnZrO*_x_*/SSZ-13 | ^[33]^ |
| 9000 | 400 | 3 | 30.6 | 93.0 | 87.9 | 3.4 | In Zr/SAPO-34 | ^[34]^ |
| 9000 | 340 | 3 | 13.1 | 20.4 | 44.8 | 1.5 | In_2_O_3_/HZSM-5(2/1) | ^[35]^ |
| 9000 | 380 | 3 | 26.2 | 96.0 | 63.9 | 9.1 | In–Zr/S  APO-34 | ^[36]^ |

# References

[1] G. Kresse, J. Hafner, Ab initio molecular dynamics for liquid metals, *Phys Rev B Condens Matter* **1993**, *47* (1), 558, https://doi.org/10.1103/physrevb.47.558.

[2] S. Grimme, J. Antony, S. Ehrlich, H. Krieg, A consistent and accurate ab initio parametrization of density functional dispersion correction (DFT-D) for the 94 elements H-Pu, *J Chem Phys* **2010**, *132* (15), 154104, https://doi.org/10.1063/1.3382344.

[3] B. Himmetoglu, A. Floris, S. de Gironcoli, M. Cococcioni, Hubbard-corrected DFT energy functionals: The LDA+U description of correlated systems, *International Journal of Quantum Chemistry* **2014**, *114* (1), 14, https://doi.org/10.1002/qua.24521.

[4] G. Di Liberto, L. A. Cipriano, G. Pacchioni, Role of Dihydride and Dihydrogen Complexes in Hydrogen Evolution Reaction on Single-Atom Catalysts, *J Am Chem Soc* **2021**, *143* (48), 20431, https://doi.org/10.1021/jacs.1c10470.

[5] Y. Wang, A. J. Page, Y. Nishimoto, H. J. Qian, K. Morokuma, S. Irle, Template Effect in the Competition between Haeckelite and Graphene Growth on Ni(111): Quantum Chemical Molecular Dynamics Simulations, *Journal of the American Chemical Society* **2011**, *133* (46), 18837, https://doi.org/10.1021/ja2064654.

[6] V. Wang, N. Xu, J.-C. Liu, G. Tang, W.-T. Geng, VASPKIT: A user-friendly interface facilitating high-throughput computing and analysis using VASP code, *Computer Physics Communications* **2021**, *267*, https://doi.org/10.1016/j.cpc.2021.108033.

[7] G. Henkelman, B. P. Uberuaga, H. Jónsson, A climbing image nudged elastic band method for finding saddle points and minimum energy paths, *The Journal of Chemical Physics* **2000**, *113* (22), 9901, https://doi.org/10.1063/1.1329672.

[8] O. Kramer, in (Ed.: O. Kramer), Springer International Publishing, Cham **2016**.

[9] Q. Zhang, K. Zhu, Y. Luo, Z. Bai, Z. Zhang, J. Li, Machine-learning-guided prediction of Cu-based electrocatalysts towards ethylene production in CO_2_ reduction, *Molecular Catalysis* **2023**, *547*, https://doi.org/10.1016/j.mcat.2023.113366.

[10] G. Lin, T. Guo, W. Lin, *et al.*, Machine Learning Accelerated Screening Advanced Single-Atom Anchored MXenes Electrocatalyst for Nitrogen Fixation, *ACS Catalysis* **2025**, 13534, https://doi.org/10.1021/acscatal.4c06914.

[11] X. Duan, Y. Li, J. Zhao, *et al.*, Machine Learning Accelerated Discovery of Entropy-Stabilized Oxide Catalysts for Catalytic Oxidation, *Journal of the American Chemical Society* **2025**, *147* (1), 651, https://doi.org/10.1021/jacs.4c12838.

[12] G. Yu, D. H. Mok, H. Y. Jang, H. D. Jung, S. Siahrostami, S. Back, Leveraging Machine learning and active motifs-based catalyst design for discovery of oxygen reduction electrocatalysts for hydrogen peroxide production, *Journal of Catalysis* **2025**, *442*, https://doi.org/10.1016/j.jcat.2024.115906.

[13] M. Zhong, K. Tran, Y. Min, *et al.*, Accelerated discovery of CO_2_ electrocatalysts using active machine learning, *Nature* **2020**, *581* (7807), 178, https://doi.org/10.1038/s41586-020-2242-8.

[14] X. Chang, Z. Lu, R. Luo, *et al.*, Microenvironment engineering of non-noble metal alloy for selective propane dehydrogenation, *Chem* **2024**, https://doi.org/10.1016/j.chempr.2024.08.017.

[15] X. Chang, Z.-J. Zhao, Z. Lu, *et al.*, Designing single-site alloy catalysts using a degree-of-isolation descriptor, *Nature Nanotechnology* **2023**, *18* (6), 611, https://doi.org/10.1038/s41565-023-01344-z.

[16] L. Wu, T. Guo, T. Li, Data-Driven High-Throughput Rational Design of Double-Atom Catalysts for Oxygen Evolution and Reduction, *Advanced Functional Materials* **2022**, *32* (31), https://doi.org/10.1002/adfm.202203439.

[17] H. Sun, Y. Li, L. Gao, *et al.*, High throughput screening of single atomic catalysts with optimized local structures for the electrochemical oxygen reduction by machine learning, *Journal of Energy Chemistry* **2023**, *81*, 349, https://doi.org/10.1016/j.jechem.2023.02.045.

[18] W. Li, G. Feng, S. Wang, *et al.*, Accelerating high-throughput screening of hydrogen peroxide production via DFT and machine learning, *Journal of Materials Chemistry A* **2023**, *11* (28), 15426, https://doi.org/10.1039/d3ta01859h.

[19] K. Tran, Z. W. Ulissi, Active learning across intermetallics to guide discovery of electrocatalysts for CO_2_ reduction and H_2_ evolution, *Nature Catalysis* **2018**, *1* (9), 696, https://doi.org/10.1038/s41929-018-0142-1.

[20] C. Fang, J. Zhou, L. Zhang, W. Wan, Y. Ding, X. Sun, Synergy of dual-atom catalysts deviated from the scaling relationship for oxygen evolution reaction, *Nature Communications* **2023**, *14* (1), 4449, https://doi.org/10.1038/s41467-023-40177-1.

[21] S. K. Kaiser, E. Fako, I. Surin, *et al.*, Performance descriptors of nanostructured metal catalysts for acetylene hydrochlorination, *Nature Nanotechnology* **2022**, *17* (6), 606, https://doi.org/10.1038/s41565-022-01105-4.

[22] D. Li, H. Xu, J. Zhu, D. Cao, Fast identification of the stability of atomically dispersed bi-atom catalysts using a structure descriptor-based model, *Journal of Materials Chemistry A* **2022**, *10* (3), 1451, https://doi.org/10.1039/d1ta08780k.

[23] Z. K. Han, D. Sarker, R. Ouyang, A. Mazheika, Y. Gao, S. V. Levchenko, Single-atom alloy catalysts designed by first-principles calculations and artificial intelligence, *Nature Communications* **2021**, *12* (1), 1833, https://doi.org/10.1038/s41467-021-22048-9.

[24] H. Yuan, Z. Li, X. C. Zeng, J. Yang, Descriptor-Based Design Principle for Two-Dimensional Single-Atom Catalysts: Carbon Dioxide Electroreduction, *J Phys Chem Lett* **2020**, *11* (9), 3481, https://doi.org/10.1021/acs.jpclett.0c00676.

[25] P. Tian, G. Zhan, J. Tian, *et al.*, Direct CO_2_ hydrogenation to light olefins over ZnZrO*_x_* mixed with hierarchically hollow SAPO-34 with rice husk as green silicon source and template, *Applied Catalysis B: Environmental* **2022**, *315*, https://doi.org/10.1016/j.apcatb.2022.121572.

[26] X. Liu, M. Wang, H. Yin, *et al.*, Tandem Catalysis for Hydrogenation of CO and CO_2_ to Lower Olefins with Bifunctional Catalysts Composed of Spinel Oxide and SAPO-34, *ACS Catalysis* **2020**, *10* (15), 8303, https://doi.org/10.1021/acscatal.0c01579.

[27] S. Dang, S. Li, C. Yang, *et al.*, Selective Transformation of CO_2_ and H_2_ into Lower Olefins over In_2_O_3_-ZnZrO*_x_*/SAPO-34 Bifunctional Catalysts, *ChemSusChem* **2019**, *12* (15), 3582, https://doi.org/10.1002/cssc.201900958.

[28] Z. Li, J. Wang, Y. Qu, *et al.*, Highly Selective Conversion of Carbon Dioxide to Lower Olefins, *ACS Catalysis* **2017**, *7* (12), 8544, https://doi.org/10.1021/acscatal.7b03251.

[29] Y. Ni, Z. Chen, Y. Fu, Y. Liu, W. Zhu, Z. Liu, Selective conversion of CO_2_ and H_2_ into aromatics, *Nature Communications* **2018**, *9* (1), https://doi.org/10.1038/s41467-018-05880-4.

[30] Z. Li, Y. Qu, J. Wang, *et al.*, Highly Selective Conversion of Carbon Dioxide to Aromatics over Tandem Catalysts, *Joule* **2019**, *3* (2), 570, https://doi.org/10.1016/j.joule.2018.10.027.

[31] J. Wei, Q. Ge, R. Yao, *et al.*, Directly converting CO_2_ into a gasoline fuel, *Nature Communications* **2017**, *8* (1), https://doi.org/10.1038/ncomms15174.

[32] S. Wang, L. Zhang, W. Zhang, *et al.*, Selective Conversion of CO_2_ into Propene and Butene, *Chem* **2020**, *6* (12), 3344, https://doi.org/10.1016/j.chempr.2020.09.025.

[33] S. Chen, J. Wang, Z. Feng, *et al.*, Hydrogenation of CO_2_ to Light Olefins over ZnZrO*_x_*/SSZ-13, *Angewandte Chemie International Edition* **2024**, *63* (8), https://doi.org/10.1002/anie.202316874.

[34] P. Gao, S. Dang, S. Li, *et al.*, Direct Production of Lower Olefins from CO_2_ Conversion via Bifunctional Catalysis, *ACS Catalysis* **2017**, *8* (1), 571, https://doi.org/10.1021/acscatal.7b02649.

[35] P. Gao, S. Li, X. Bu, *et al.*, Direct conversion of CO_2_ into liquid fuels with high selectivity over a bifunctional catalyst, *Nature Chemistry* **2017**, *9* (10), 1019, https://doi.org/10.1038/nchem.2794.

[36] S. Dang, P. Gao, Z. Liu, *et al.*, Role of zirconium in direct CO_2_ hydrogenation to lower olefins on oxide/zeolite bifunctional catalysts, *Journal of Catalysis* **2018**, *364*, 382, https://doi.org/10.1016/j.jcat.2018.06.010.
